# Supplementary material for: MicroRNA expression profile in Lampetra morii upon Vibrio anguillarum infection and miR-4561 characterization targeting lip
Source: Commun Biol. 2021 Aug 20;4:995. doi: 10.1038/s42003-021-02525-z (PMC8379177; doi:10.1038/s42003-021-02525-z)
Supplement: Supplementary file 2 — Supplementary information. [file 42003_2021_2525_MOESM2_ESM.pdf]

Description of supplementary files

Supplementary Figure legends

Figure S1 miRNA expression profile in the three libraries.

Top 10 expressed miRNAs are shown in pie charts. The size of each section corresponds to the relative abundance of each miRNAs. (left) uninfected lampreys. (middle) 8 h of infection. (right) 17 d of infection.

Figure S2 Uncropped western blot images.

Figure S3 Other uncropped images in this study.

Supplementary Table legends

Table S1 Primers used in this study.

Table S2 Differentially expressed miRNAs, 0 h vs. 8 h.

Table S3 Differentially expressed miRNAs, 17 d vs. 0 h.

Table S4 Differentially expressed miRNAs, 17 d vs. 8 h.

Table S5 miRNA target gene prediction.

Table S6 Embryo hatching rate statistics.

Table S7 Top 10 expressed miRNAs in each group.

Supplementary Dataset legends

Supplementary Data 1. All source data underlying the graphs and charts in this study.

Supplementary Figure S1

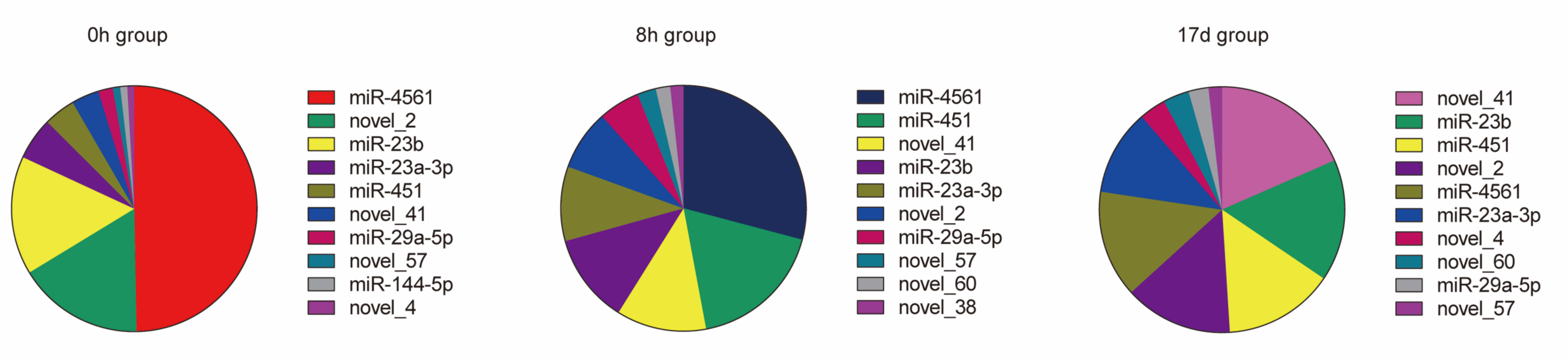

Supplementary Figure S2

Figure3d（β-actin）

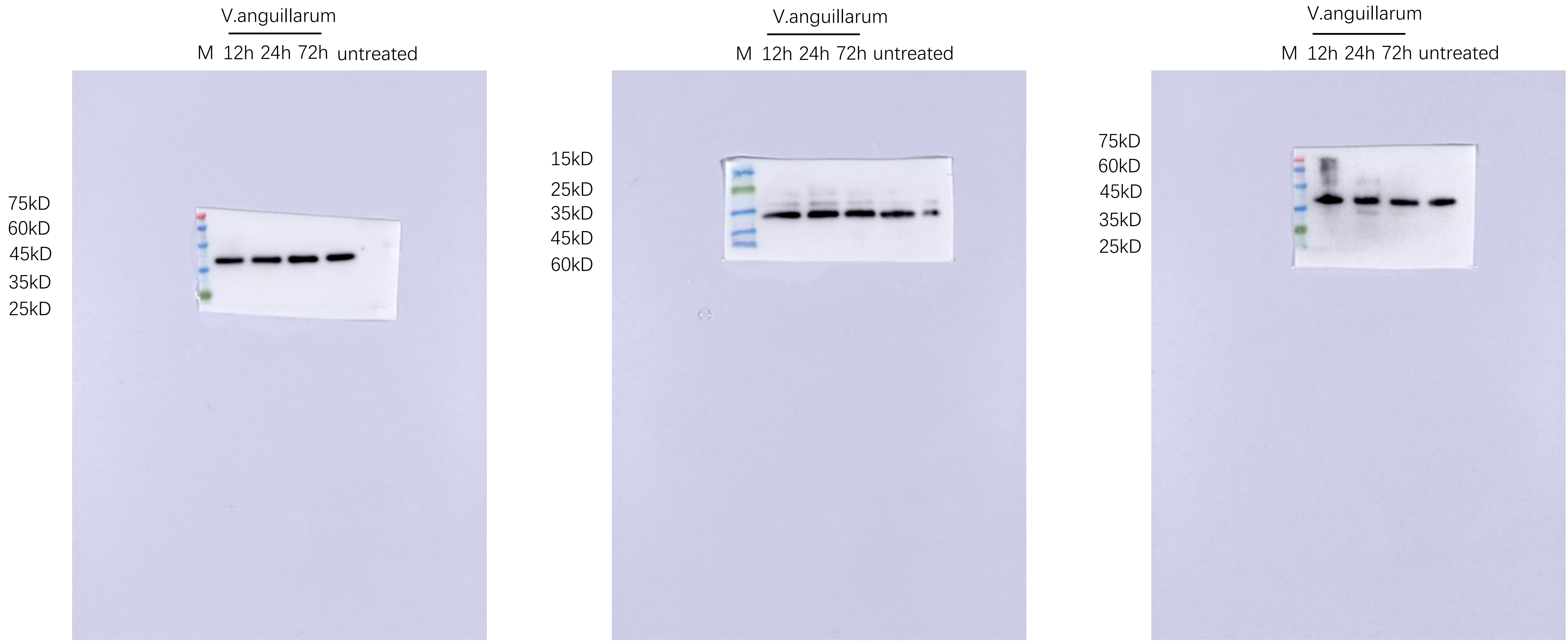

Supplementary Figure S2, continued

Figure3d（LIP）

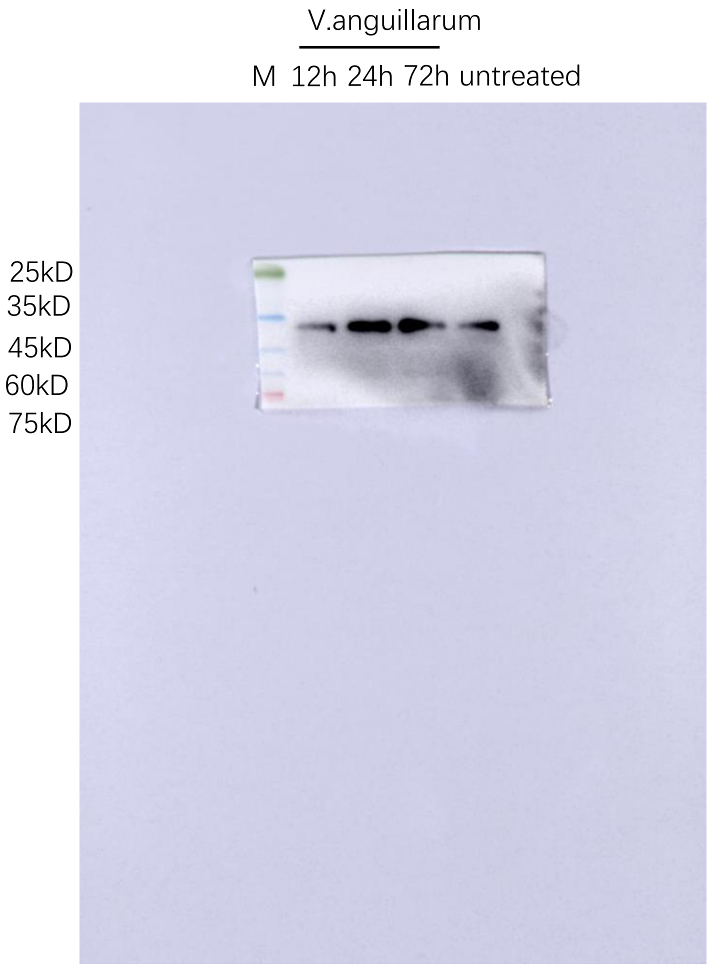

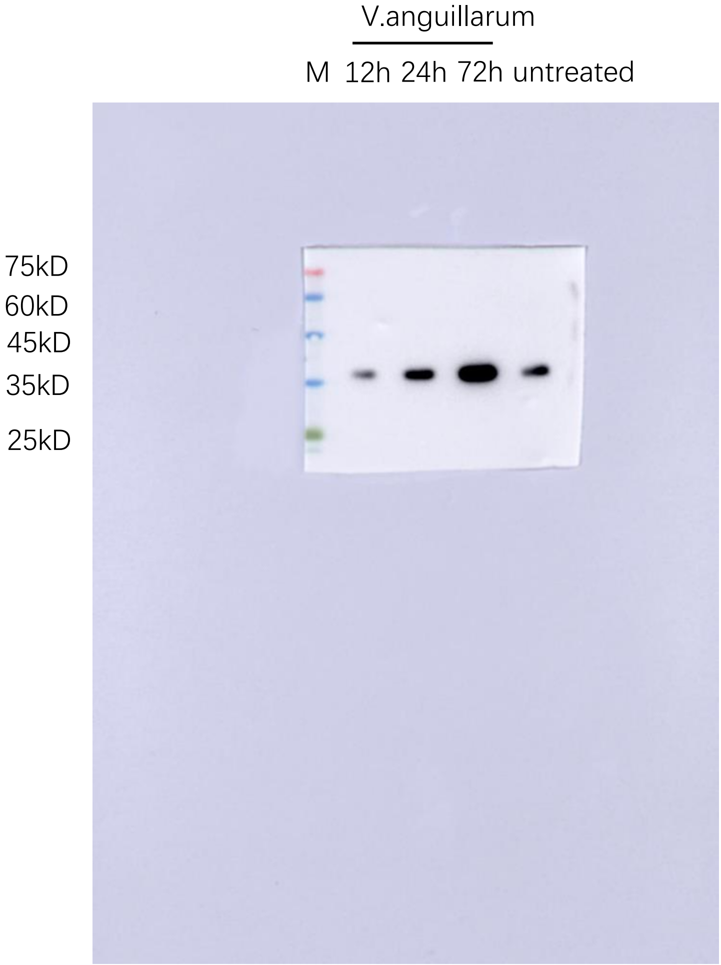

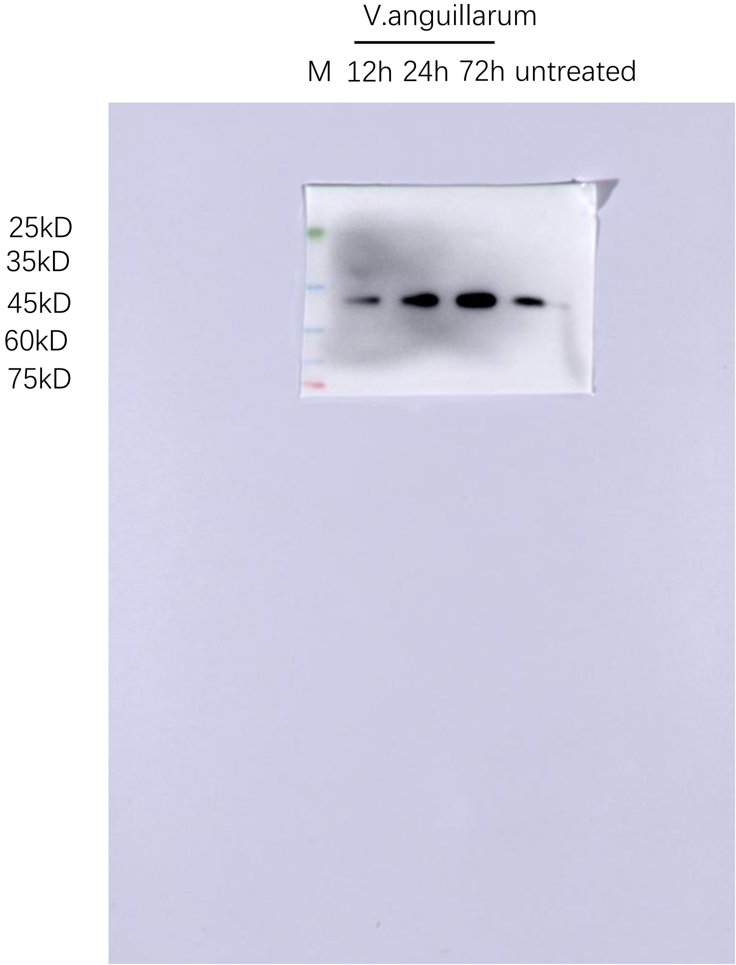

Supplementary Figure S2, continued

Figure4d

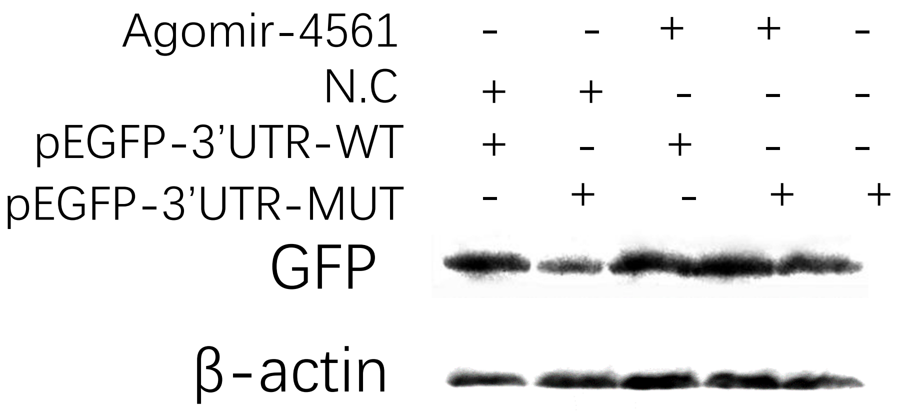

Supplementary Figure S2, continued

Figure5b（inhibitor-β-actin）

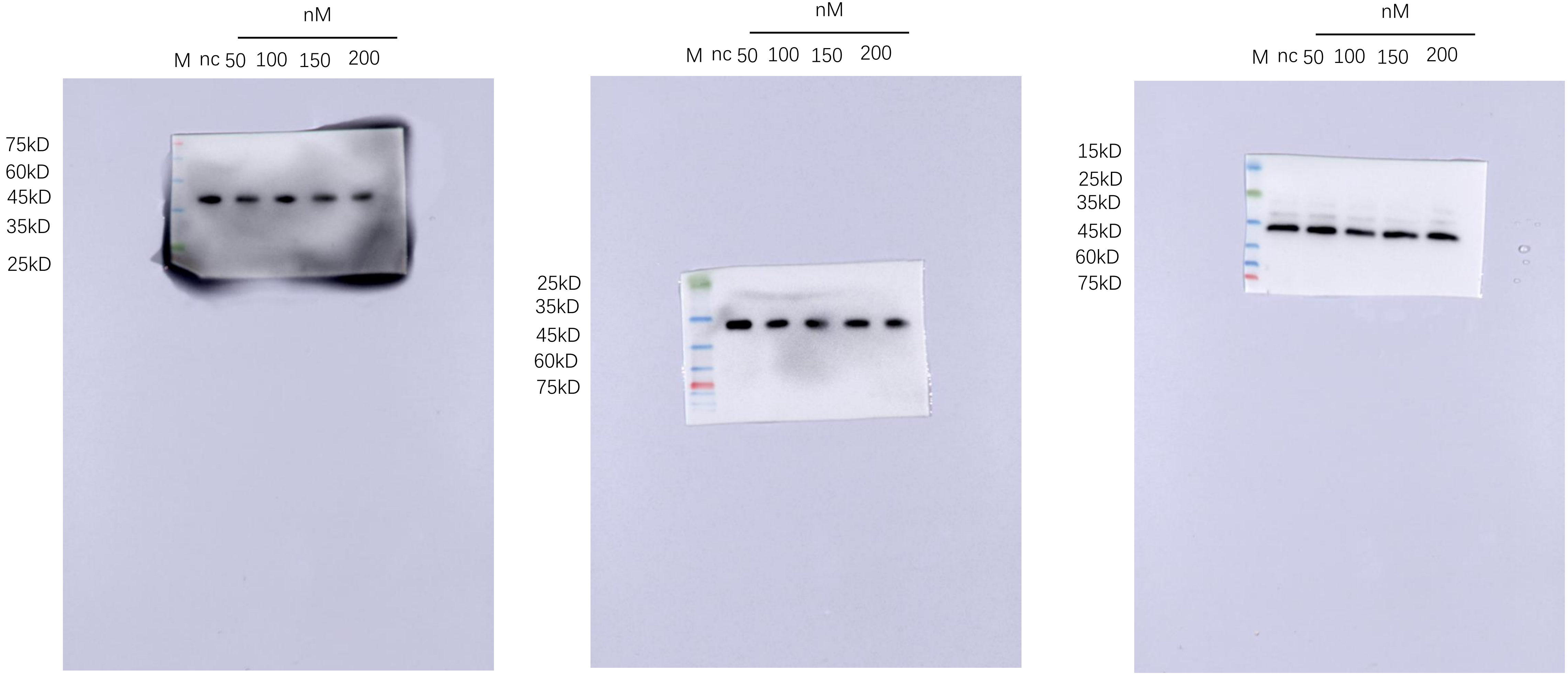

Supplementary Figure S2, continued

Figure5b（inhibitor-LIP）

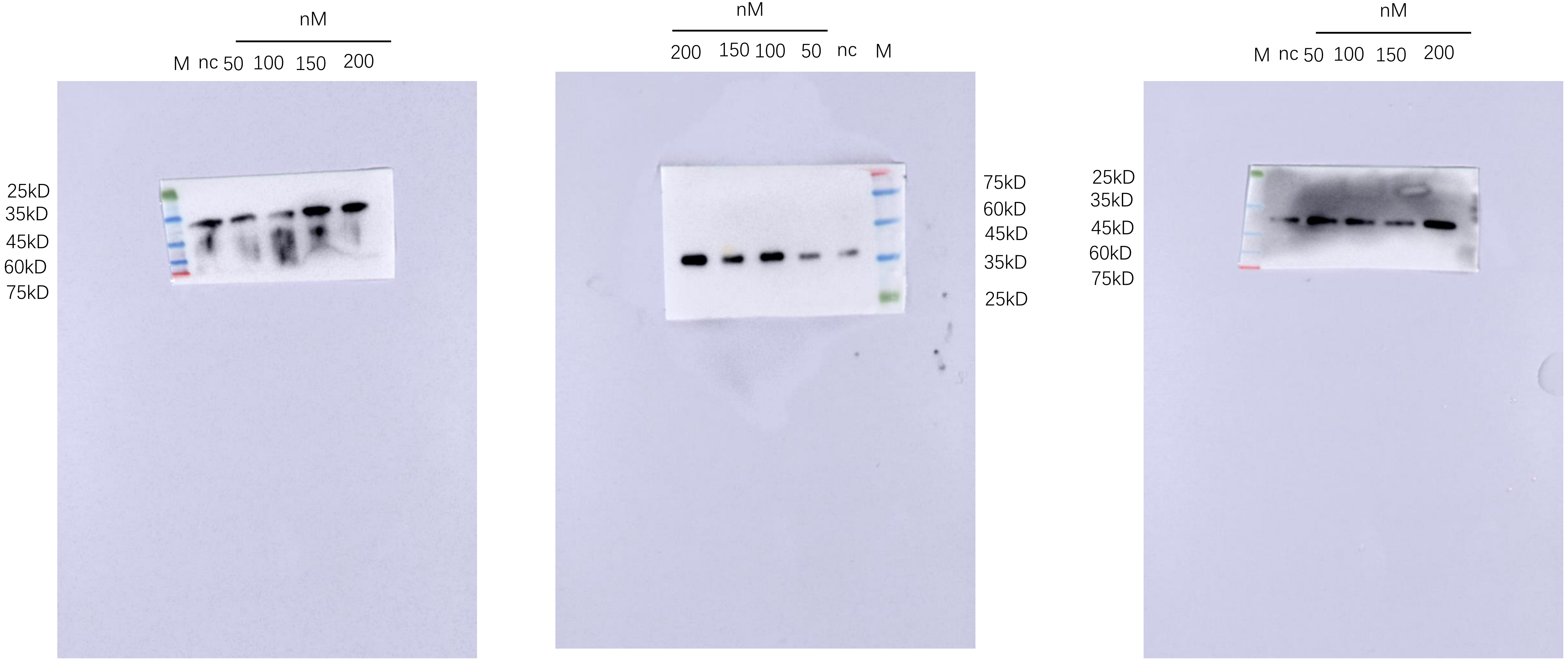

Supplementary Figure S2, continued

Figure5b（mimics-β-actin）

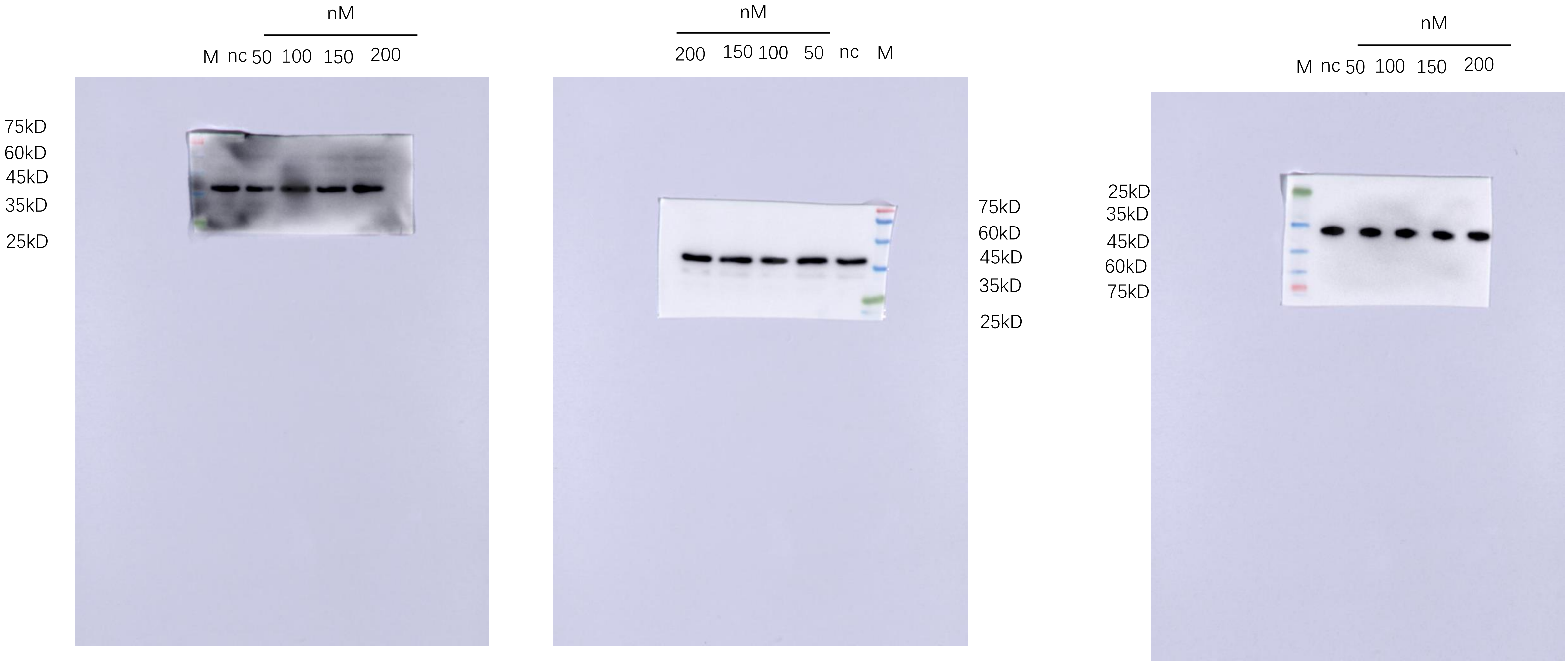

Supplementary Figure S2, continued

Figure5b（mimics-LIP）

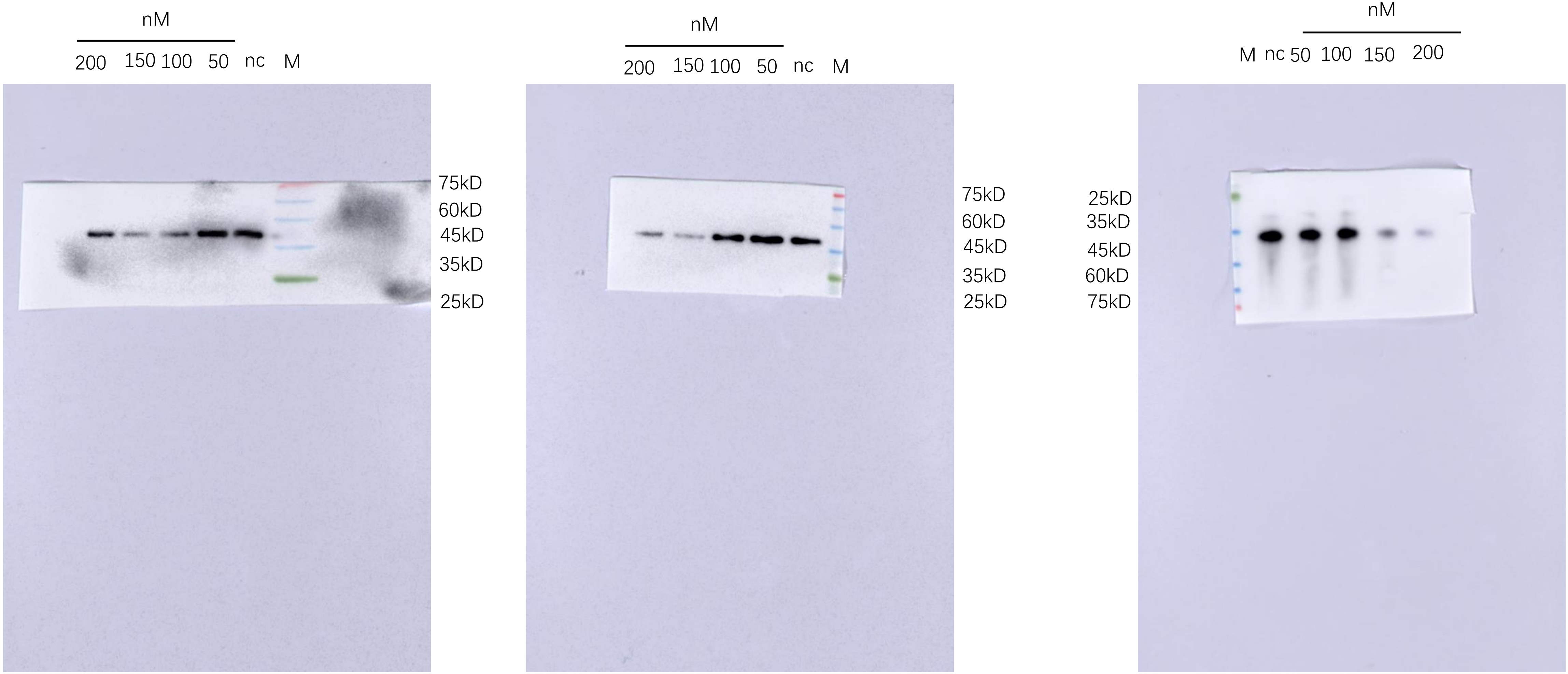

Supplementary Figure S3

Figure4c

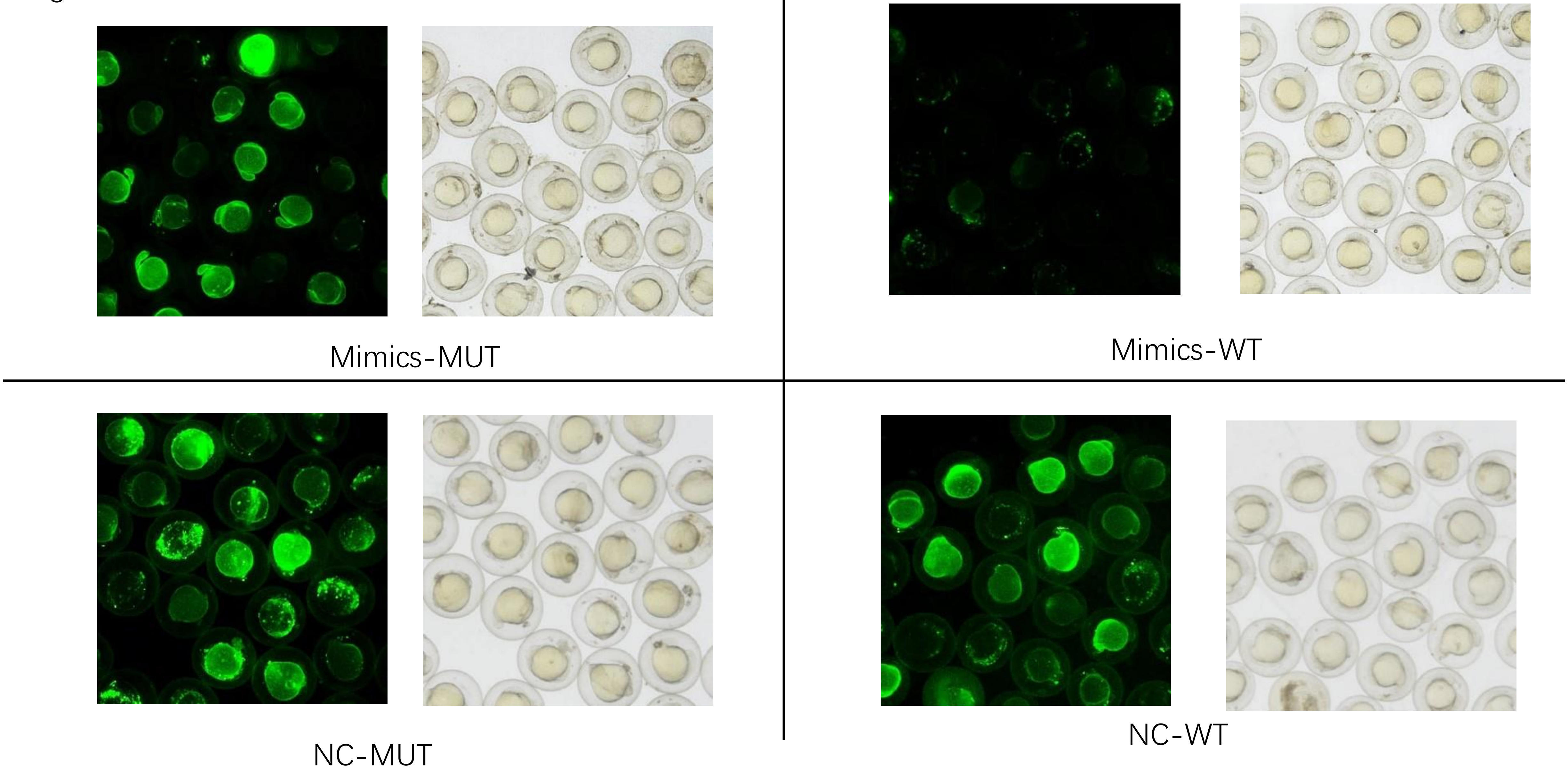

Supplementary Figure S3, continued

Figure4c

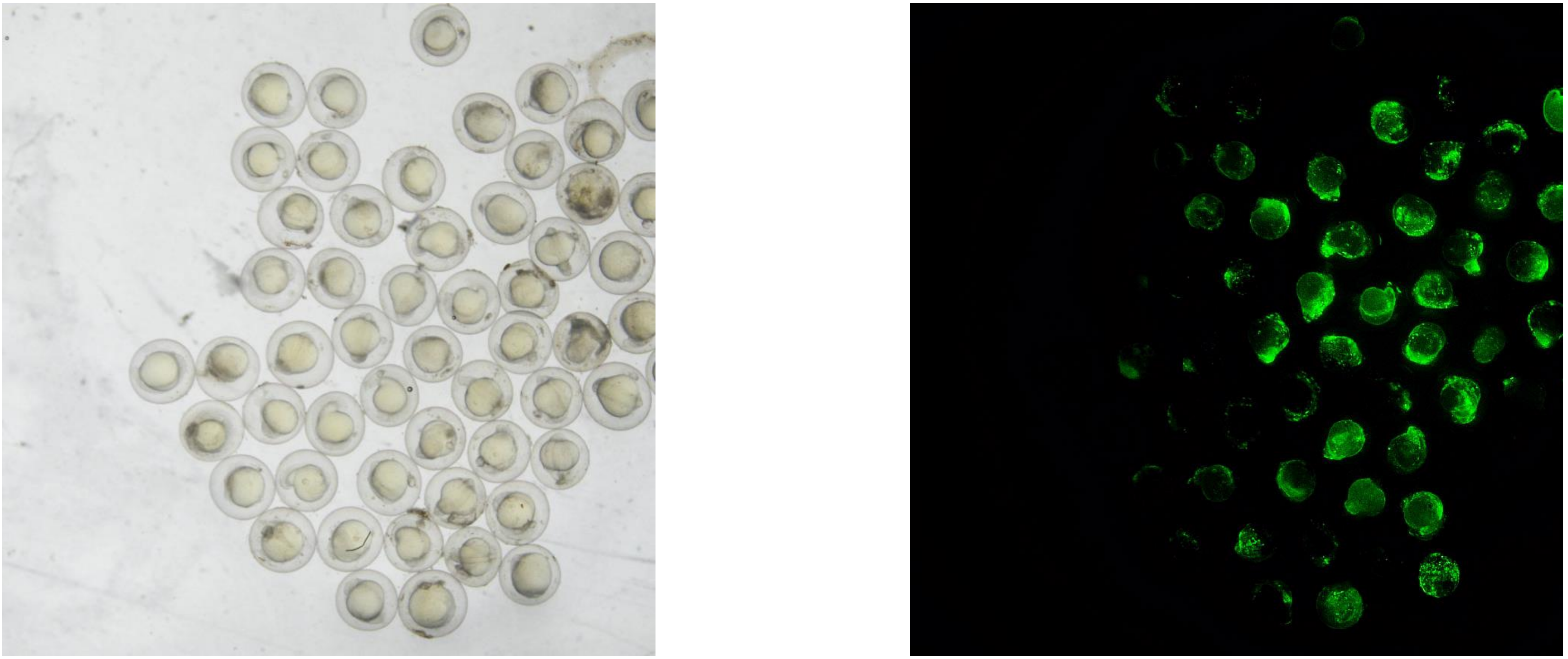

WT

Supplementary Figure S3, continued

Figure5a
（100μM）

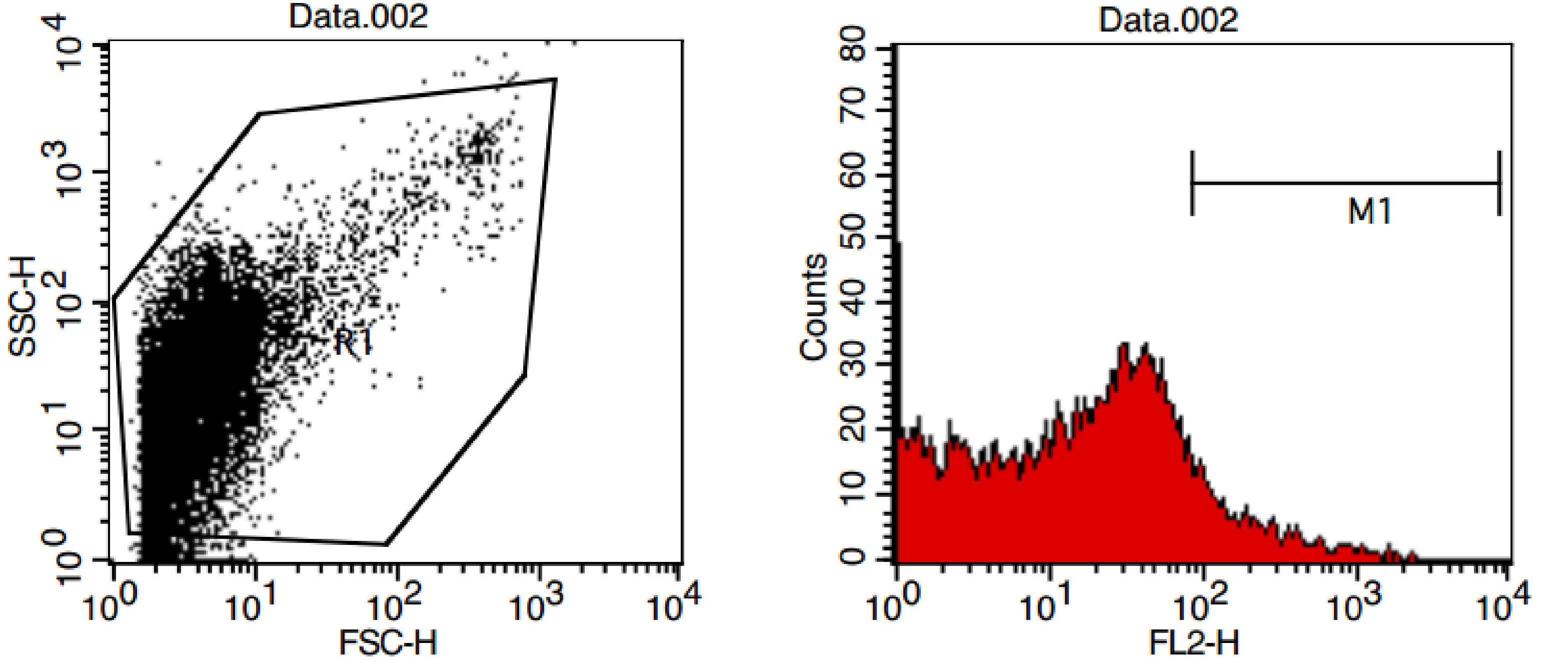

Acquisition Date: 20-Mar-19
Gate: G1
Gated Events: 10000
Total Events: 11510
X Parameter: FL2-H (Log)

Events	% Gated	% Total	Mean	Geo Mean	Peak Ch
10000	100.00	86.88	34.60	10.09	1
795	7.95	6.91	218.20	172.66	91

Supplementary Figure S3, continued

Figure5a
（200μM）

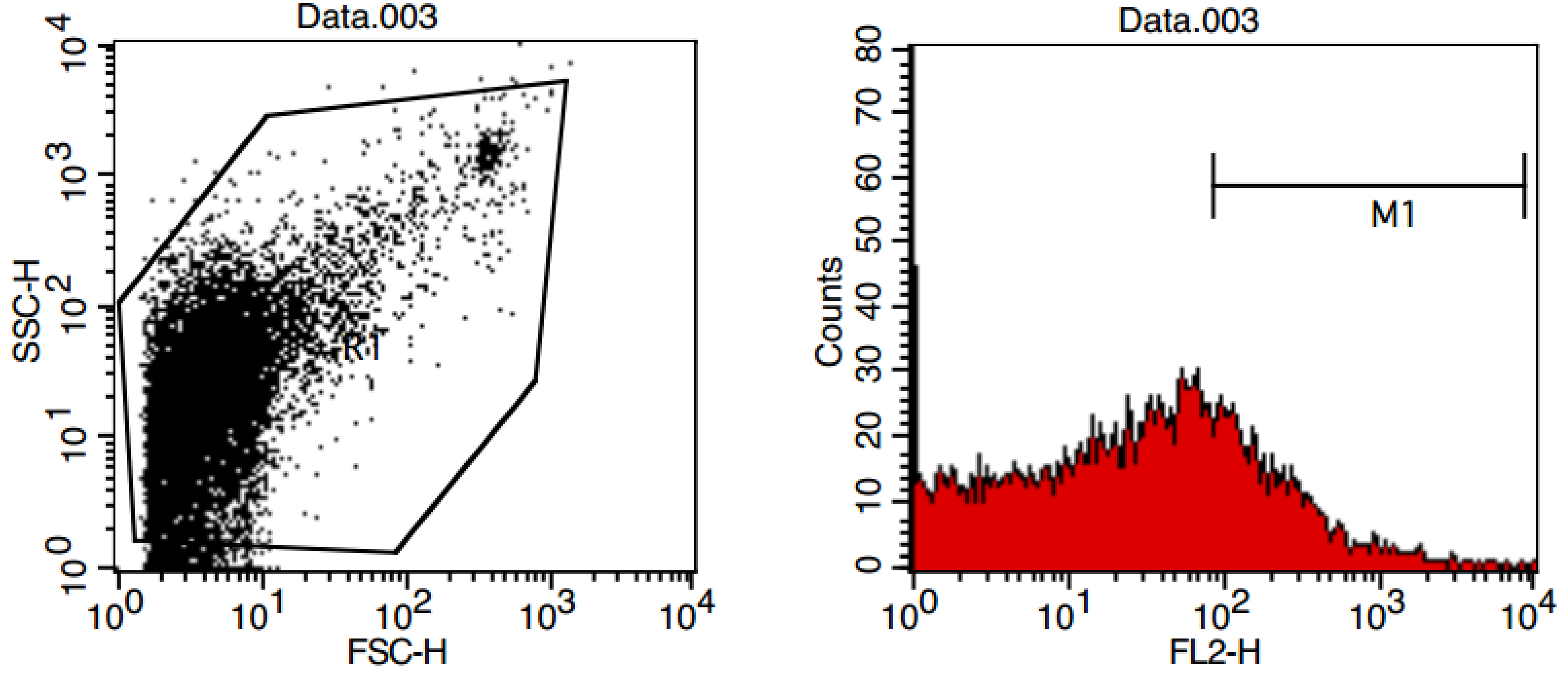

Acquisition Date: 20-Mar-19
Gate: G1
Gated Events: 10000
Total Events: 11439
X Parameter: FL2-H (Log)

Events	% Gated	% Total	Mean	Geo Mean	Peak Ch
10000	100.00	87.42	80.50	17.58	1
2224	22.24	19.44	282.59	197.08	93

Supplementary Figure S3, continued

Figure5a
(300μM)

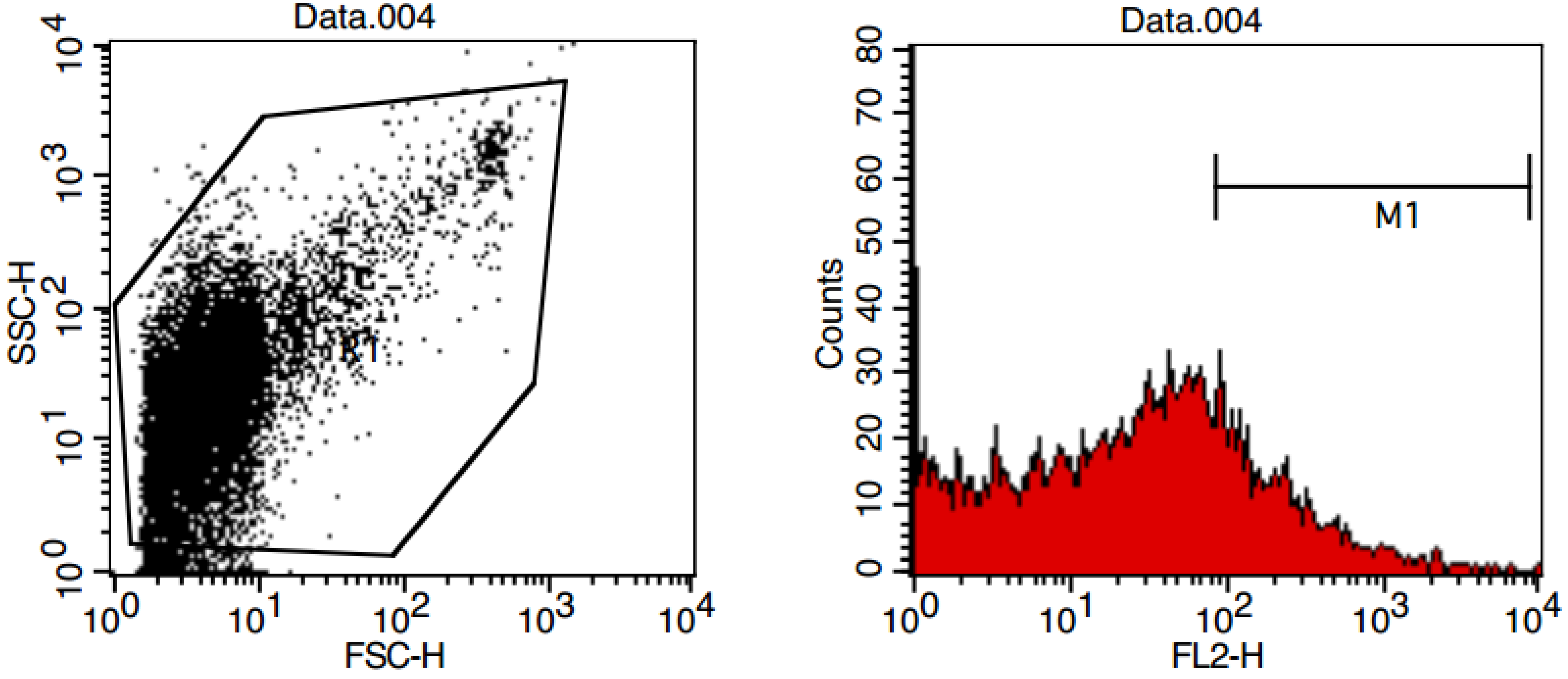

Acquisition Date: 20-Mar-19
Gate: G1
Gated Events: 10000
Total Events: 11418
X Parameter: FL2-H (Log)

Events	% Gated	% Total	Mean	Geo Mean	Peak Ch
10000	100.00	87.58	73.29	17.22	1
2000	20.00	17.52	274.21	197.97	86

Supplementary Figure S3, continued

Figure7a

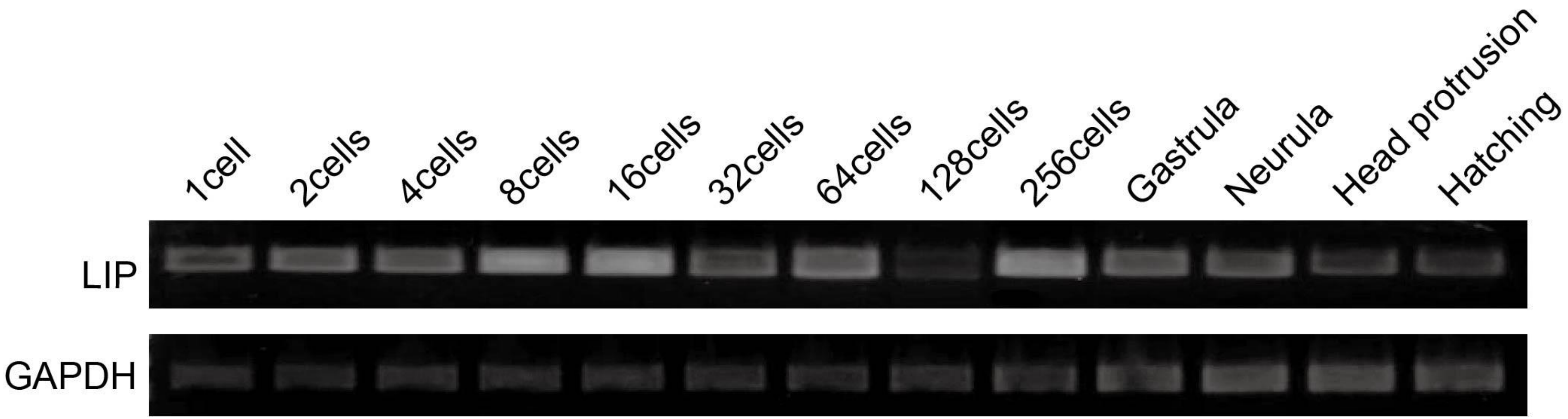

Supplementary Figure S3, continued

Figure7b
Nc
（IgG-20X）

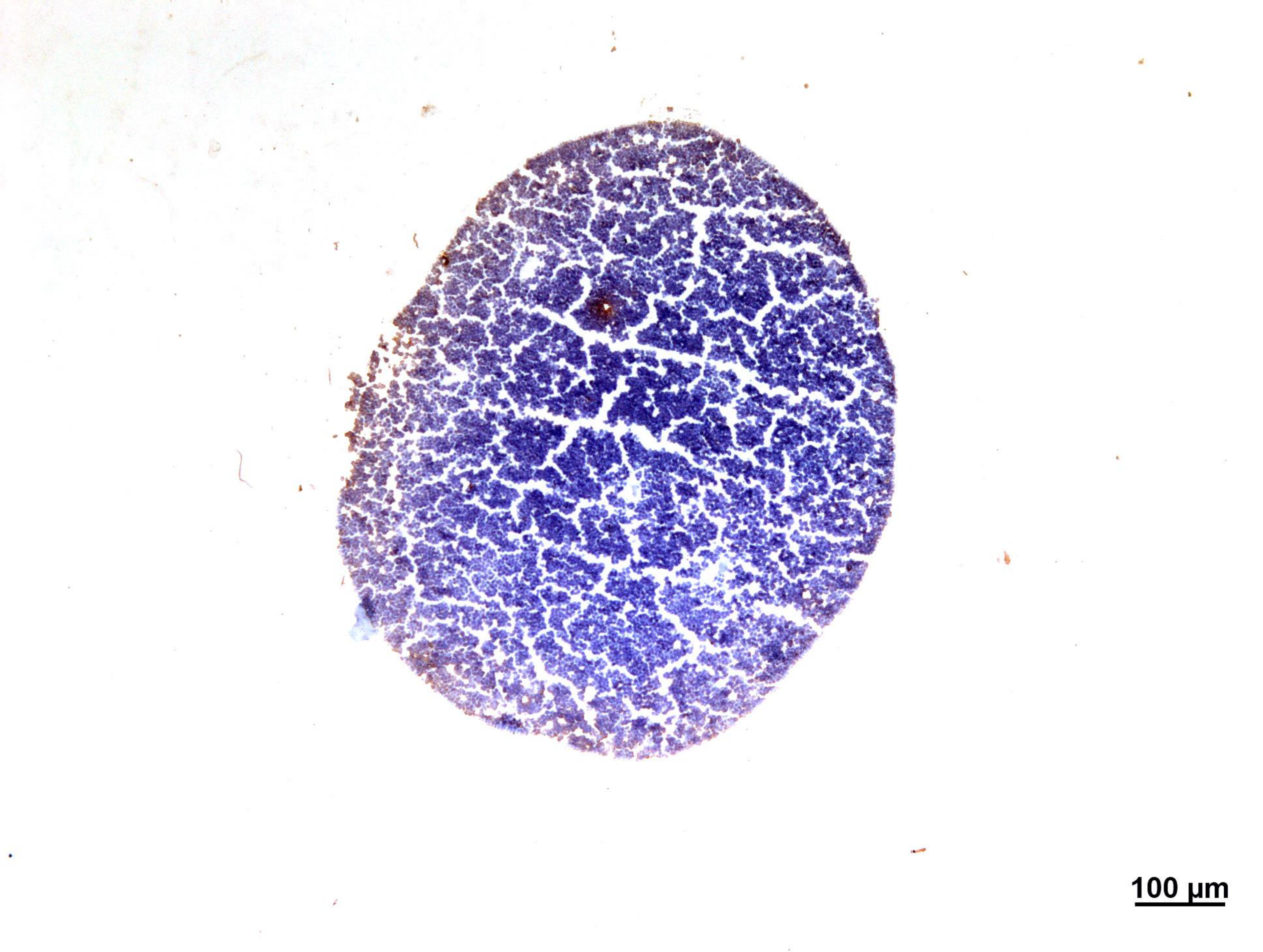

Supplementary Figure S3, continued

Figure7b
Nc
（IgG-63X）

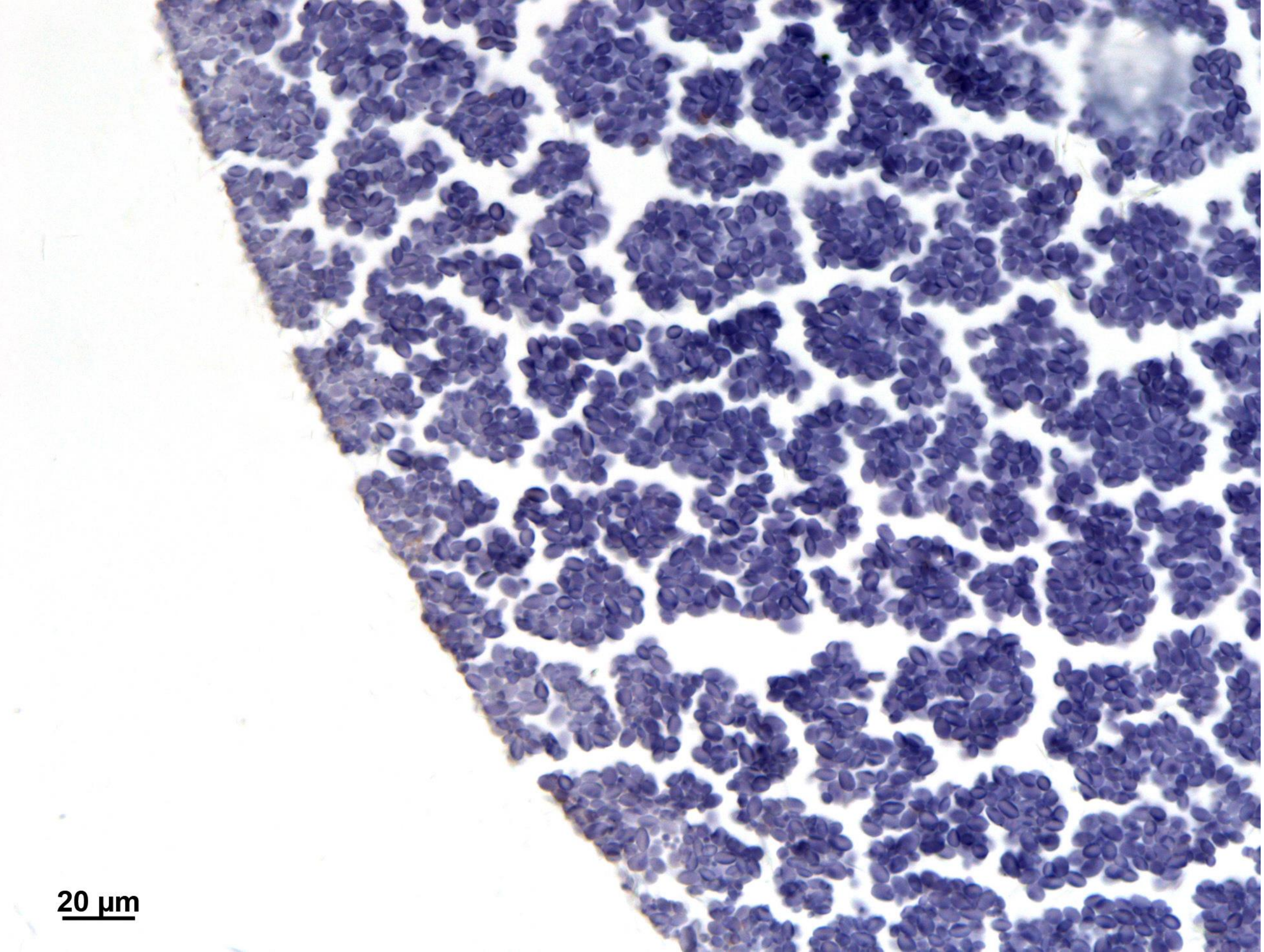

Supplementary Figure S3, continued

Figure7b
Nc
（LIP-20X)

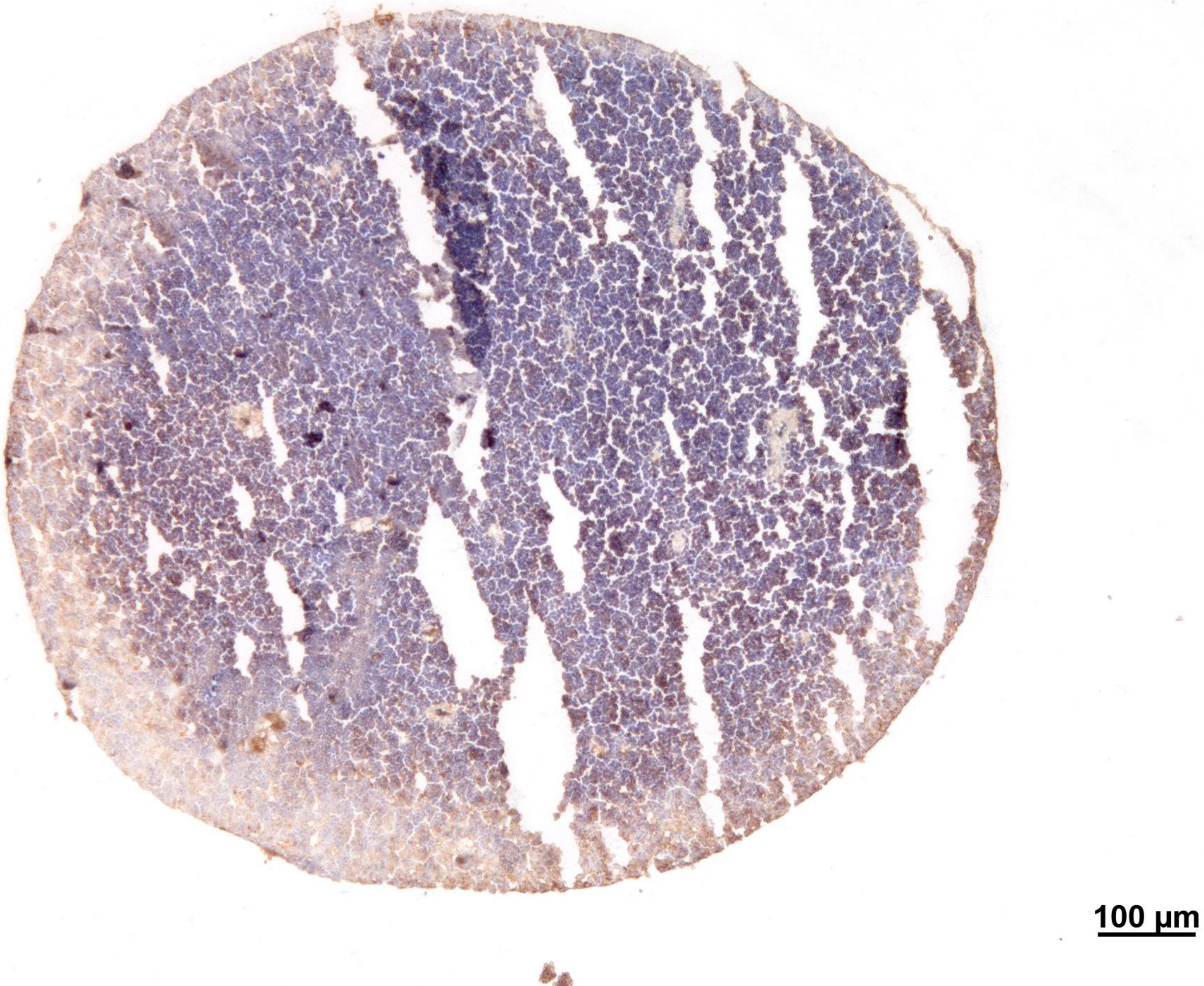

Supplementary Figure S3, continued

Figure7b
Nc
（LIP-63X)

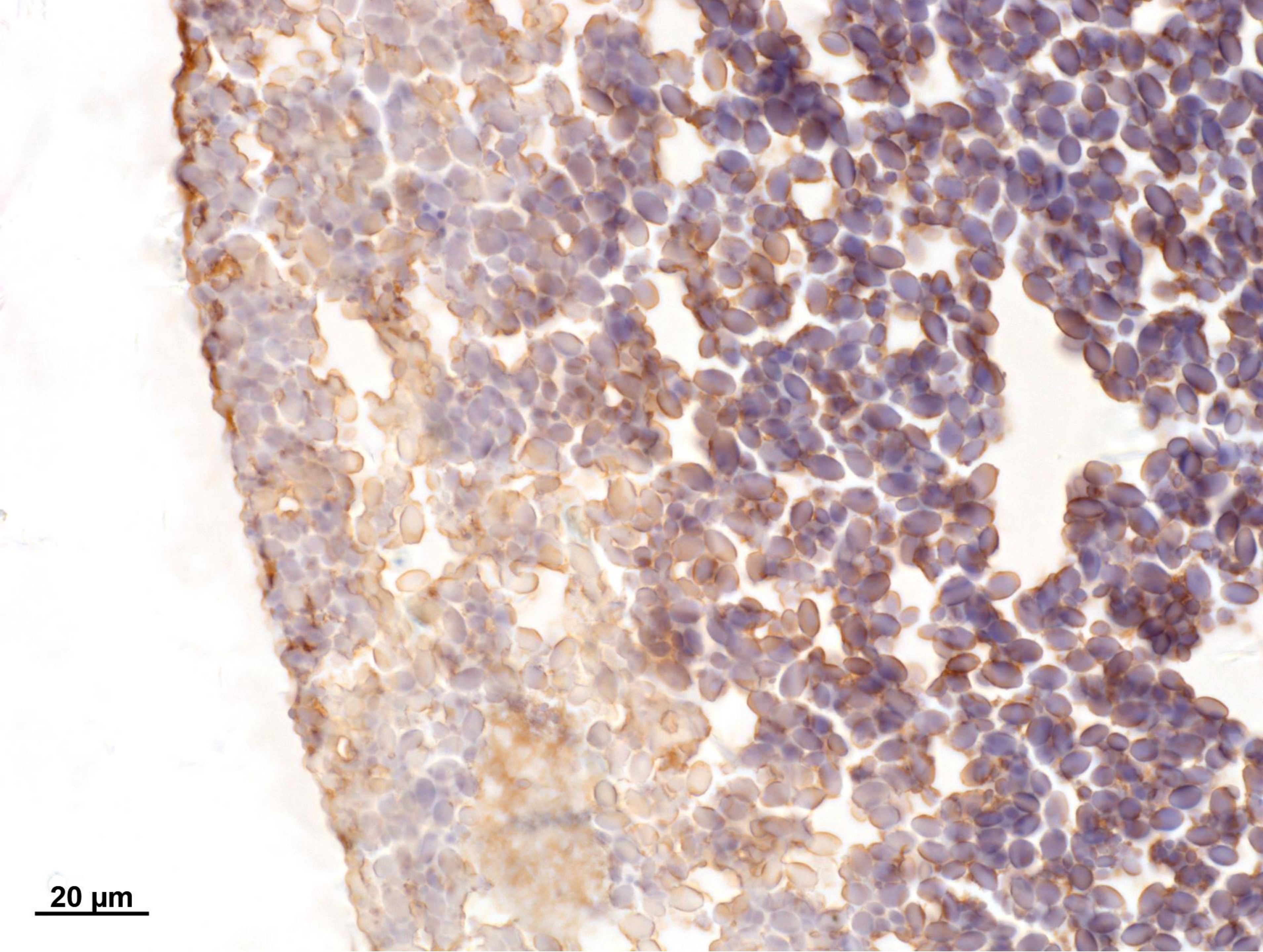

Supplementary Figure S3, continued

Figure7b
Mimics
（IgG-20X）

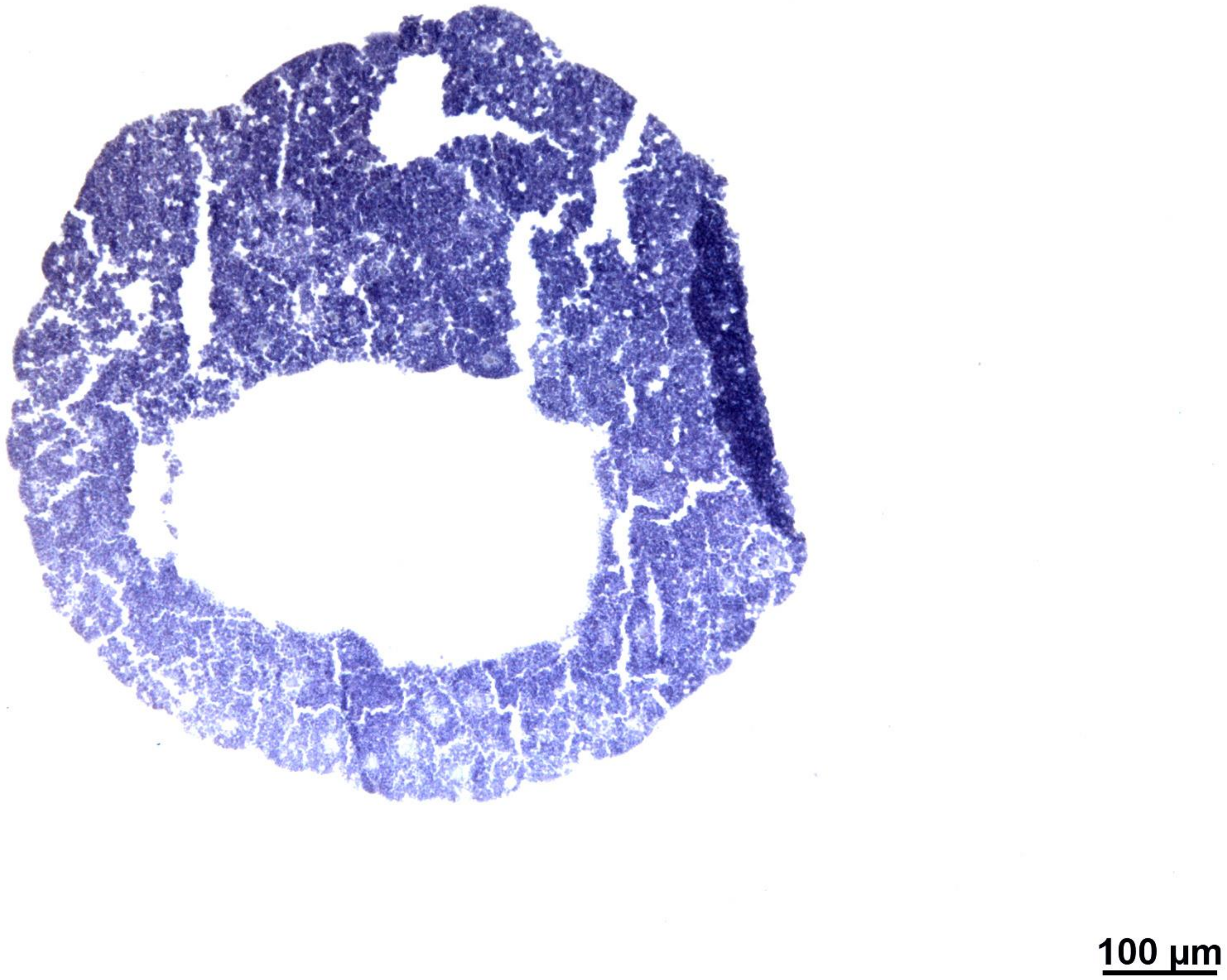

Supplementary Figure S3, continued

Figure7b
Mimics
（IgG-63X）

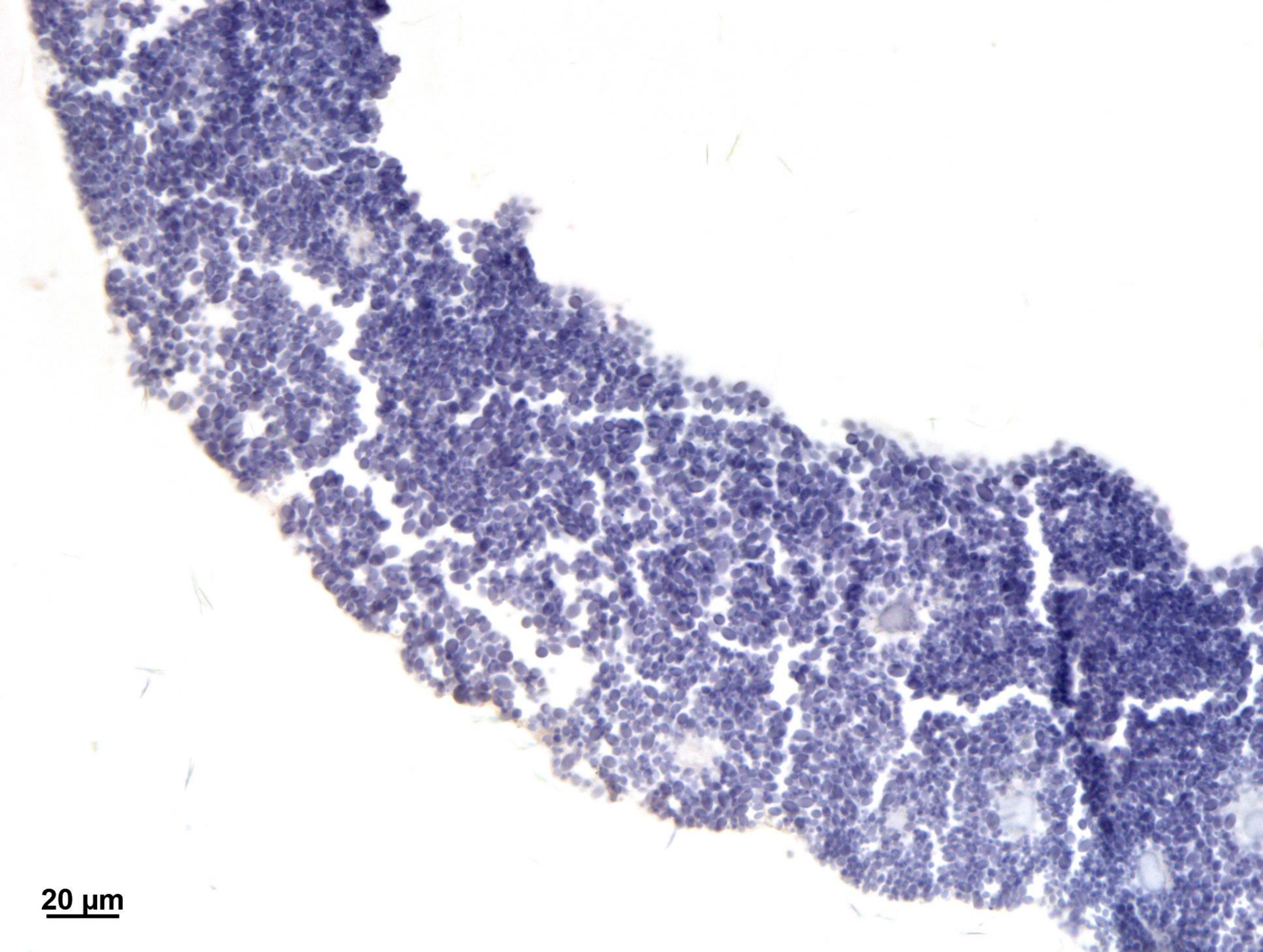

Supplementary Figure S3, continued

Figure7b
mimics
（LIP-20X）

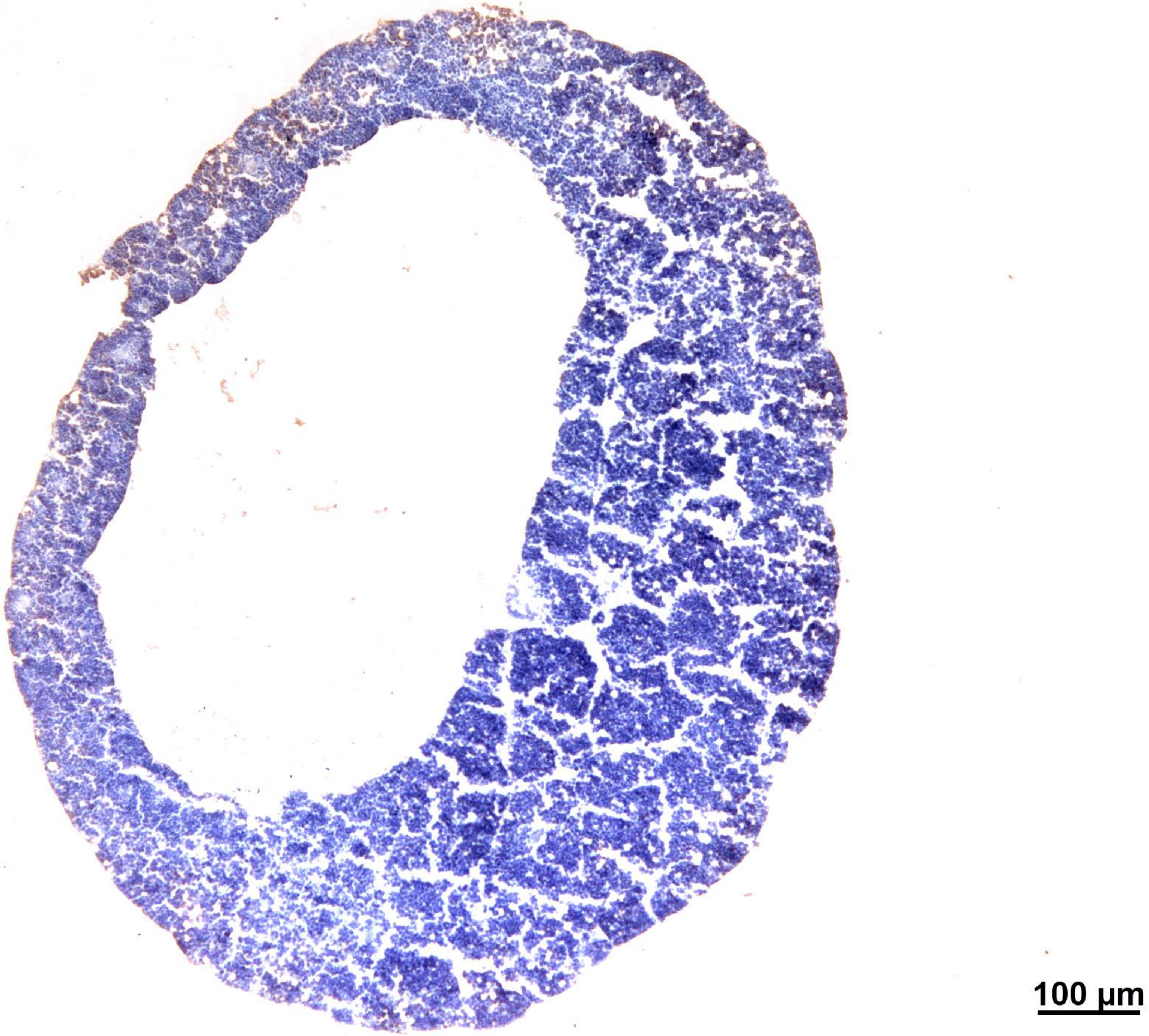

Supplementary Figure S3, continued

Figure7b
mimics
（LIP-63X）

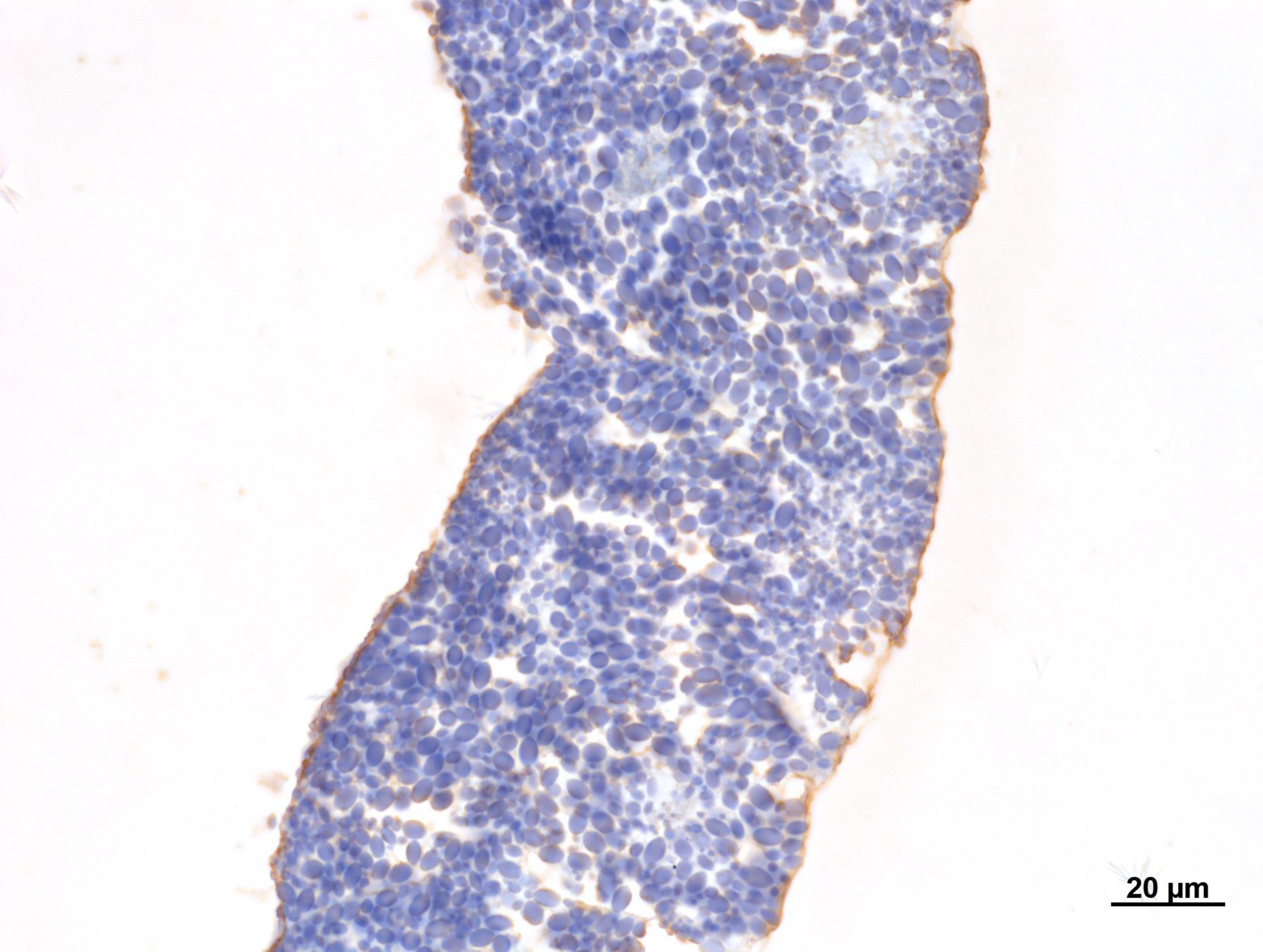

Supplementary Figure S3, continued

Figure7b
inhibitor
（IgG-20X)

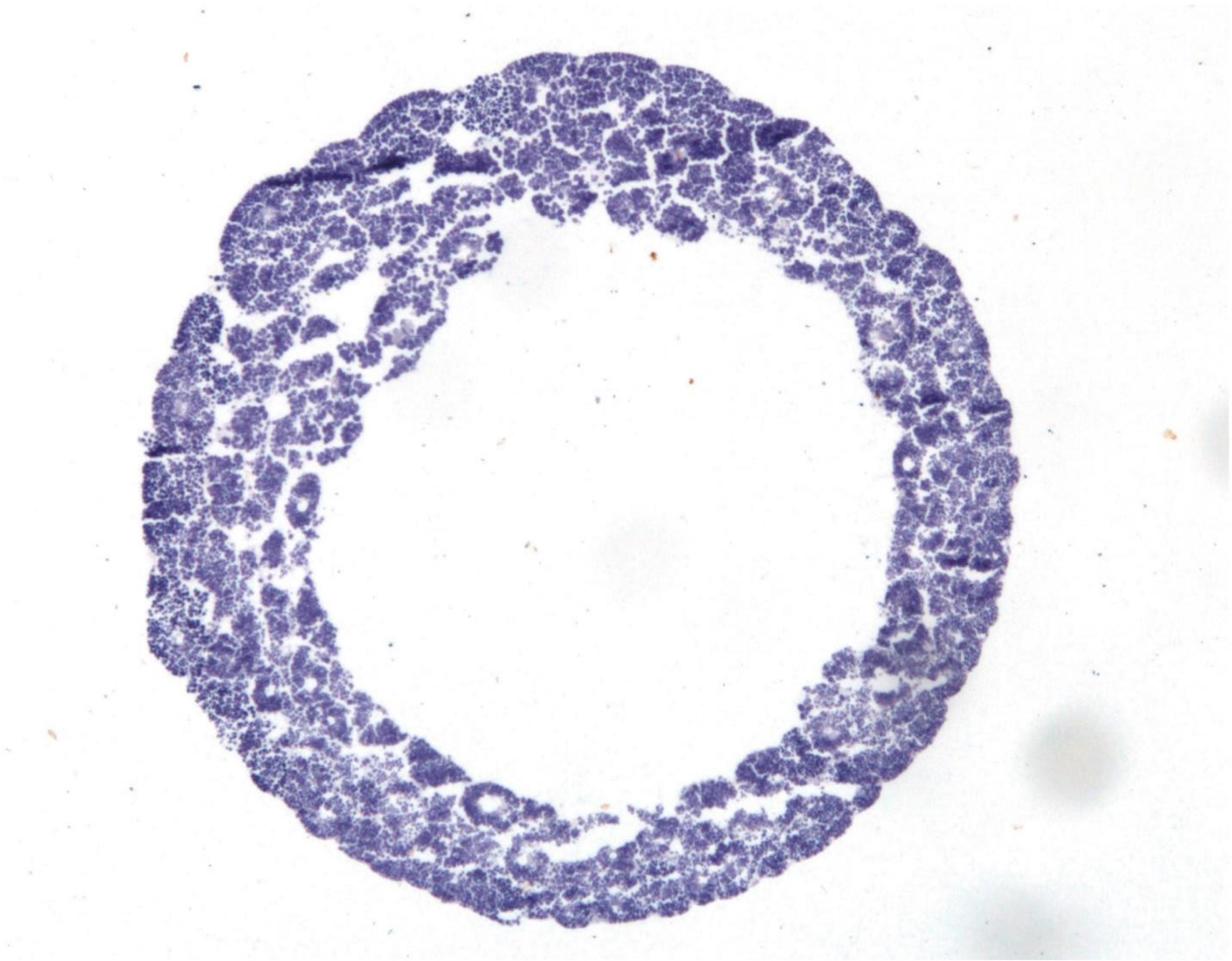

Supplementary Figure S3, continued

Figure7b
inhibitor
（IgG-63X）

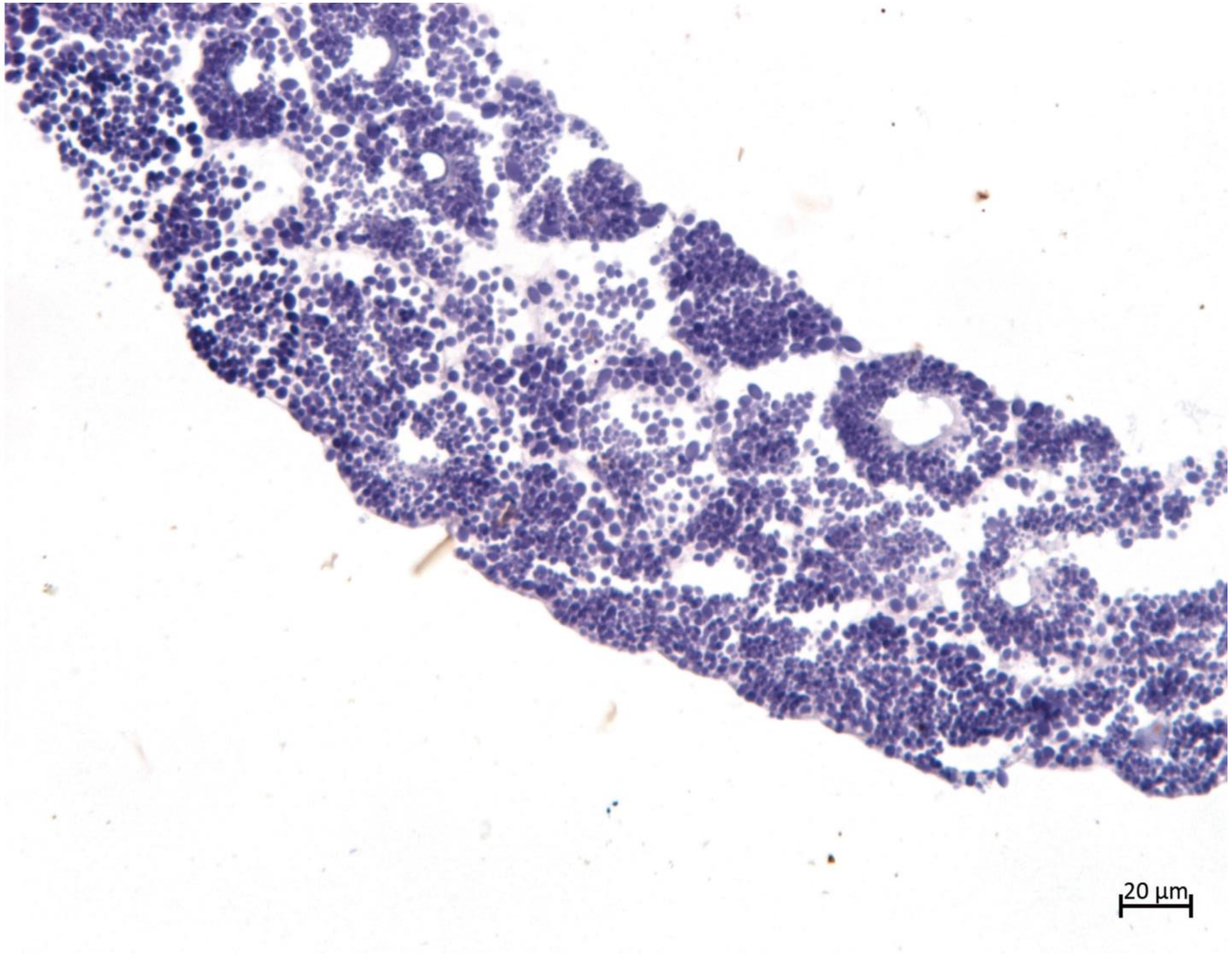

Supplementary Figure S3, continued

Figure7b
inhibitor
（LIP-20X）

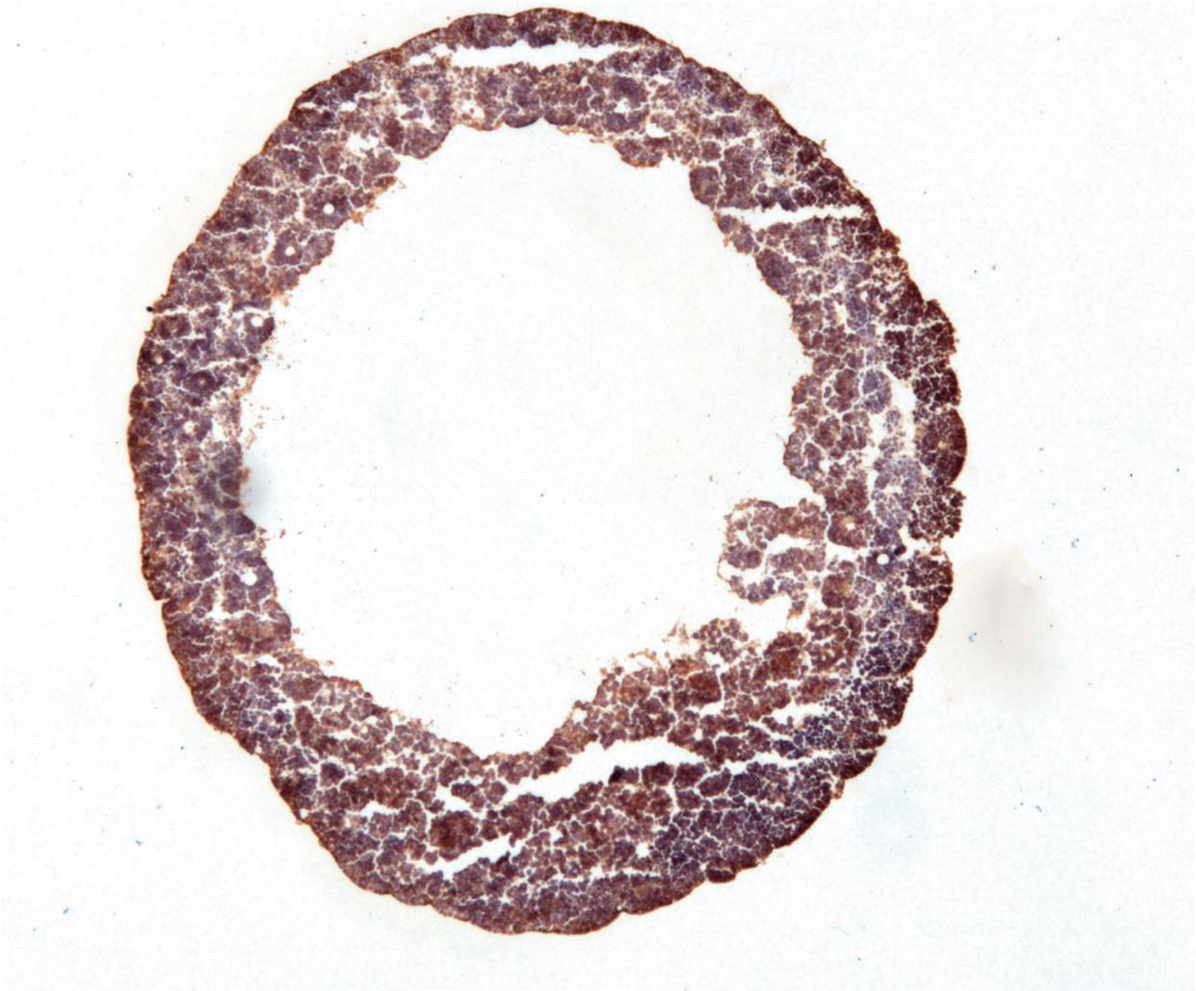

Supplementary Figure S3, continued

Figure7b
inhibitor
（LIP-63X）

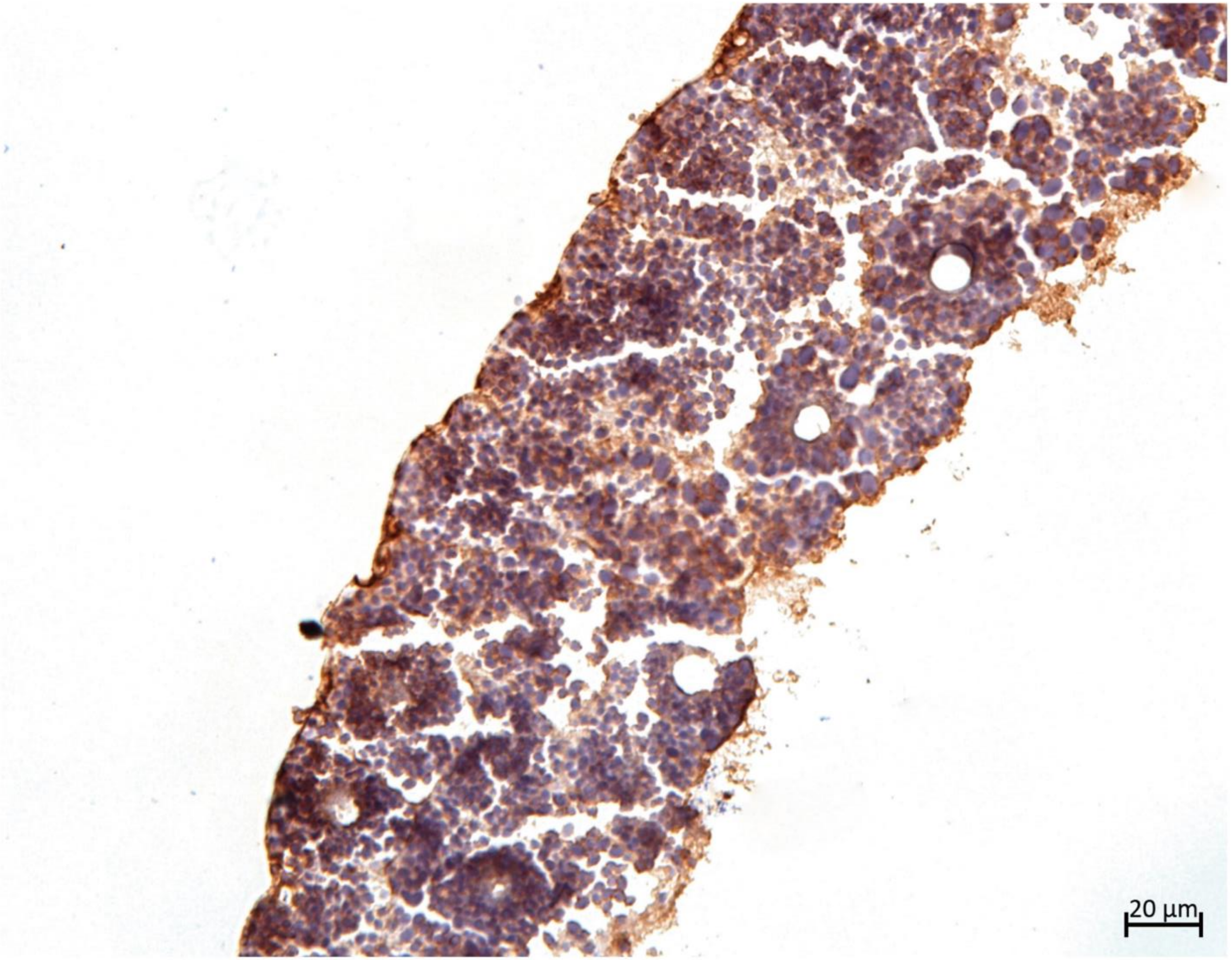

Supplementary Figure S3, continued

Figure7c（mimics）

zygote

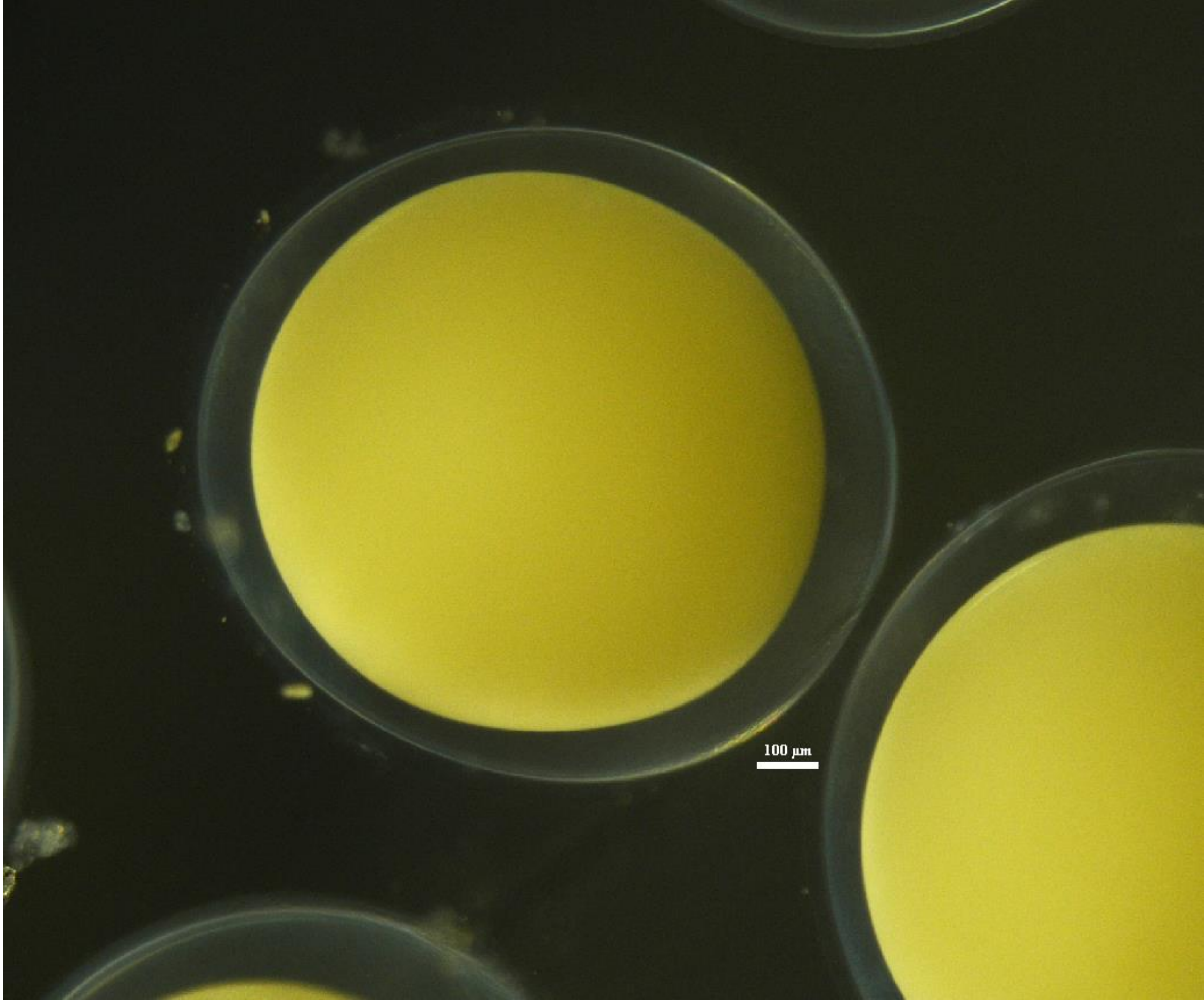

Supplementary Figure S3, continued

Figure7c（mimics）

2-cell

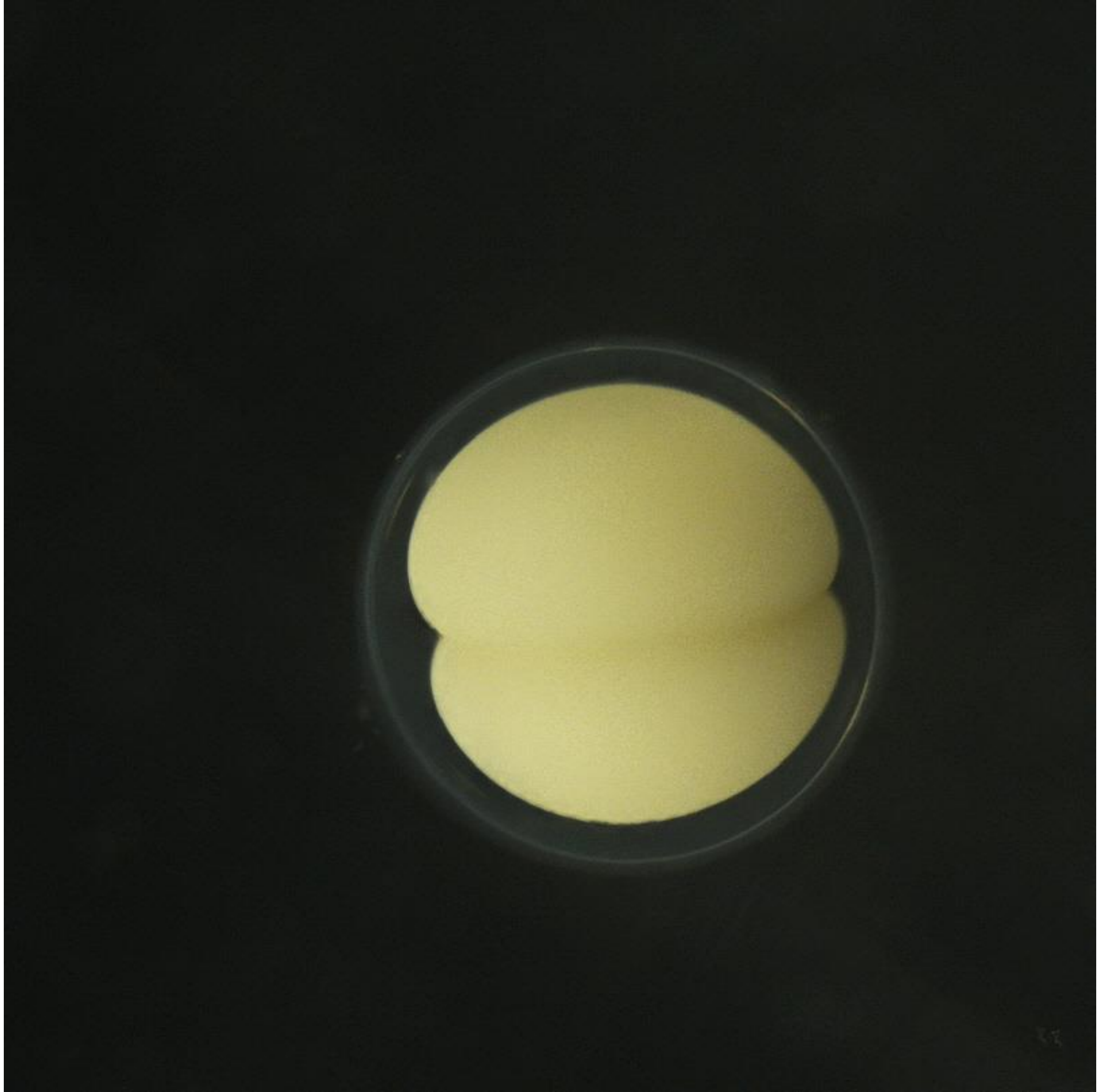

Supplementary Figure S3, continued

Figure7c（mimics）

4-cell

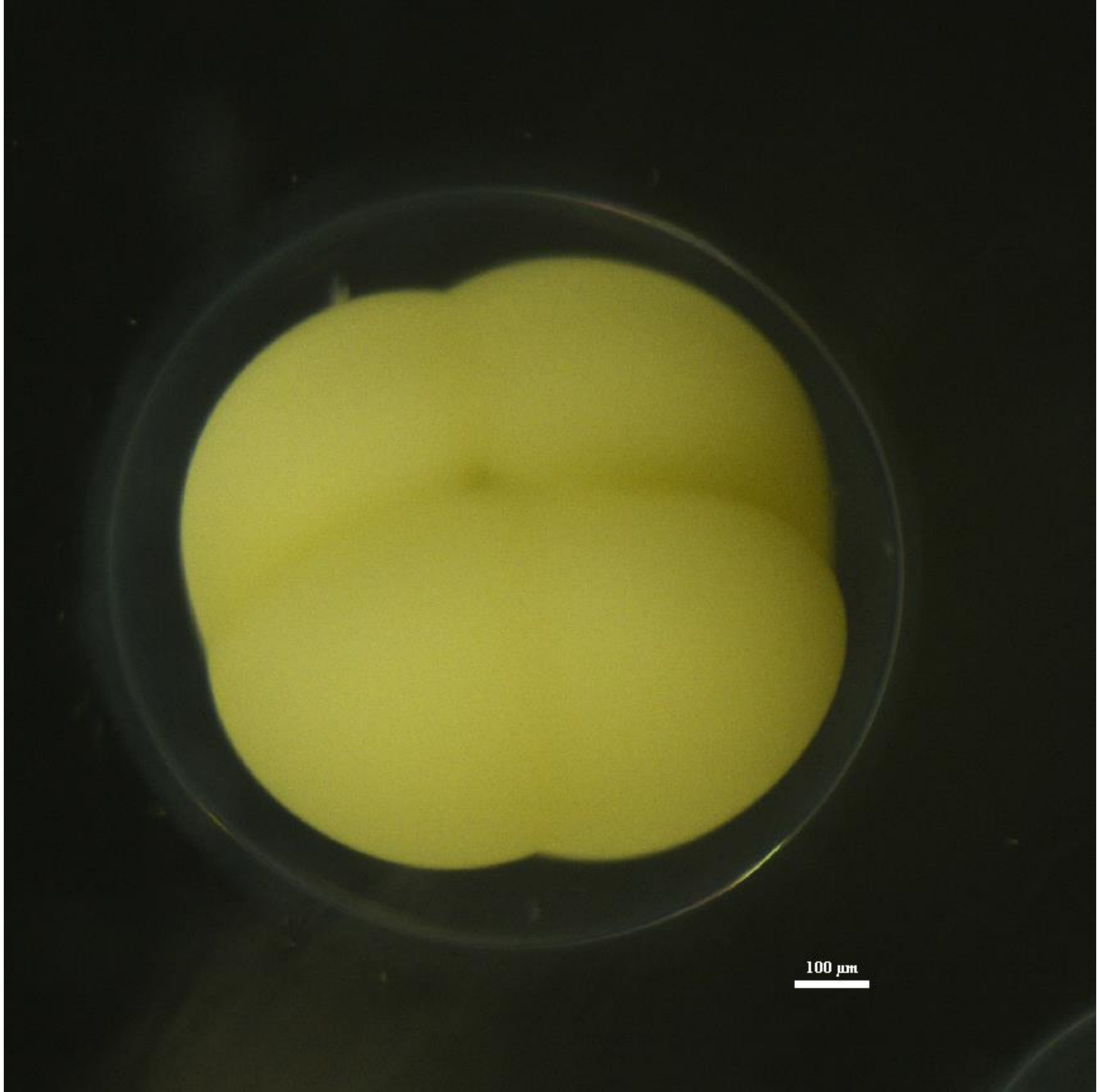

Supplementary Figure S3, continued

Figure7c（mimics）

8-cell

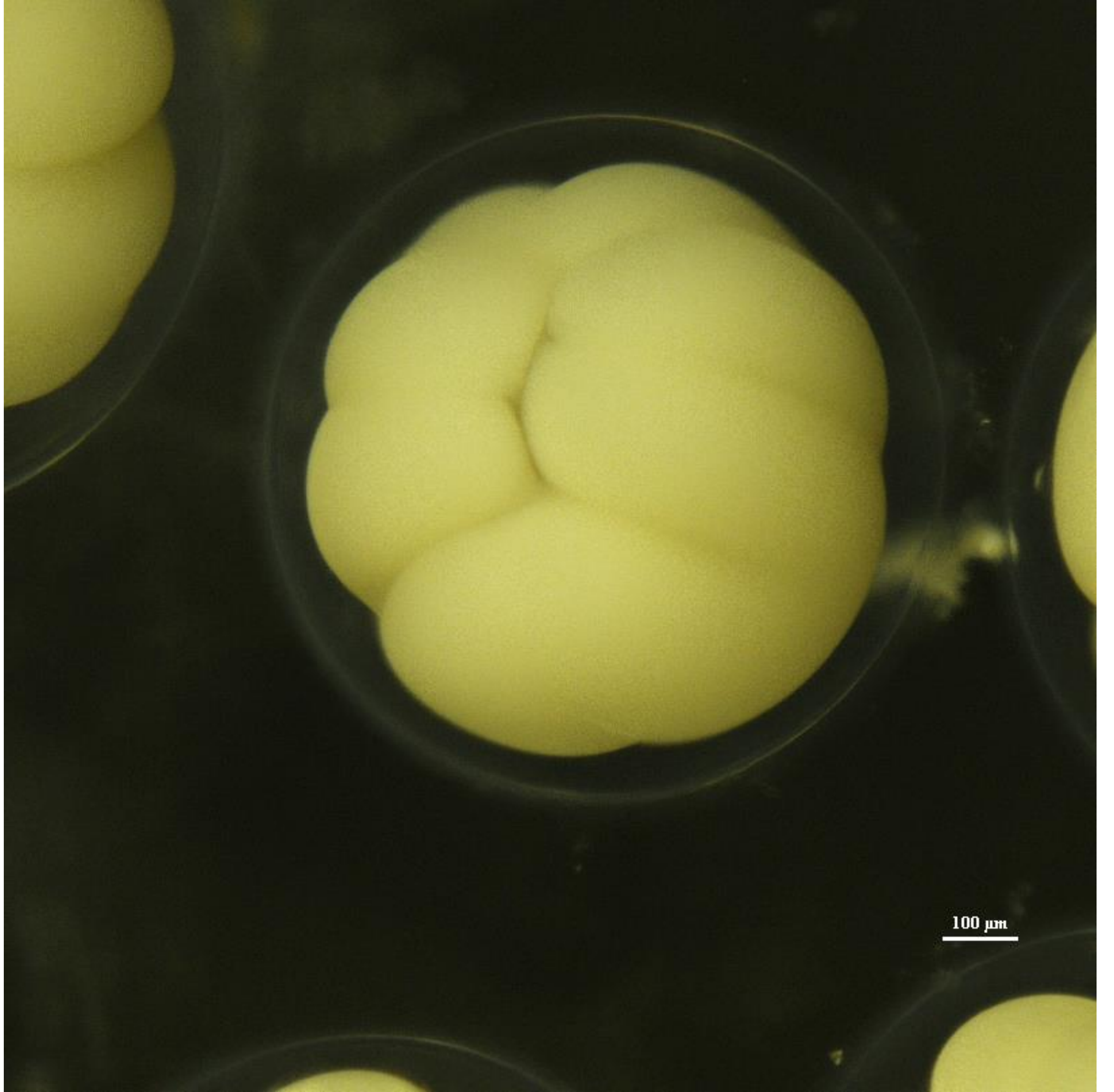

Supplementary Figure S3, continued

Figure7c（mimics）

16-cell

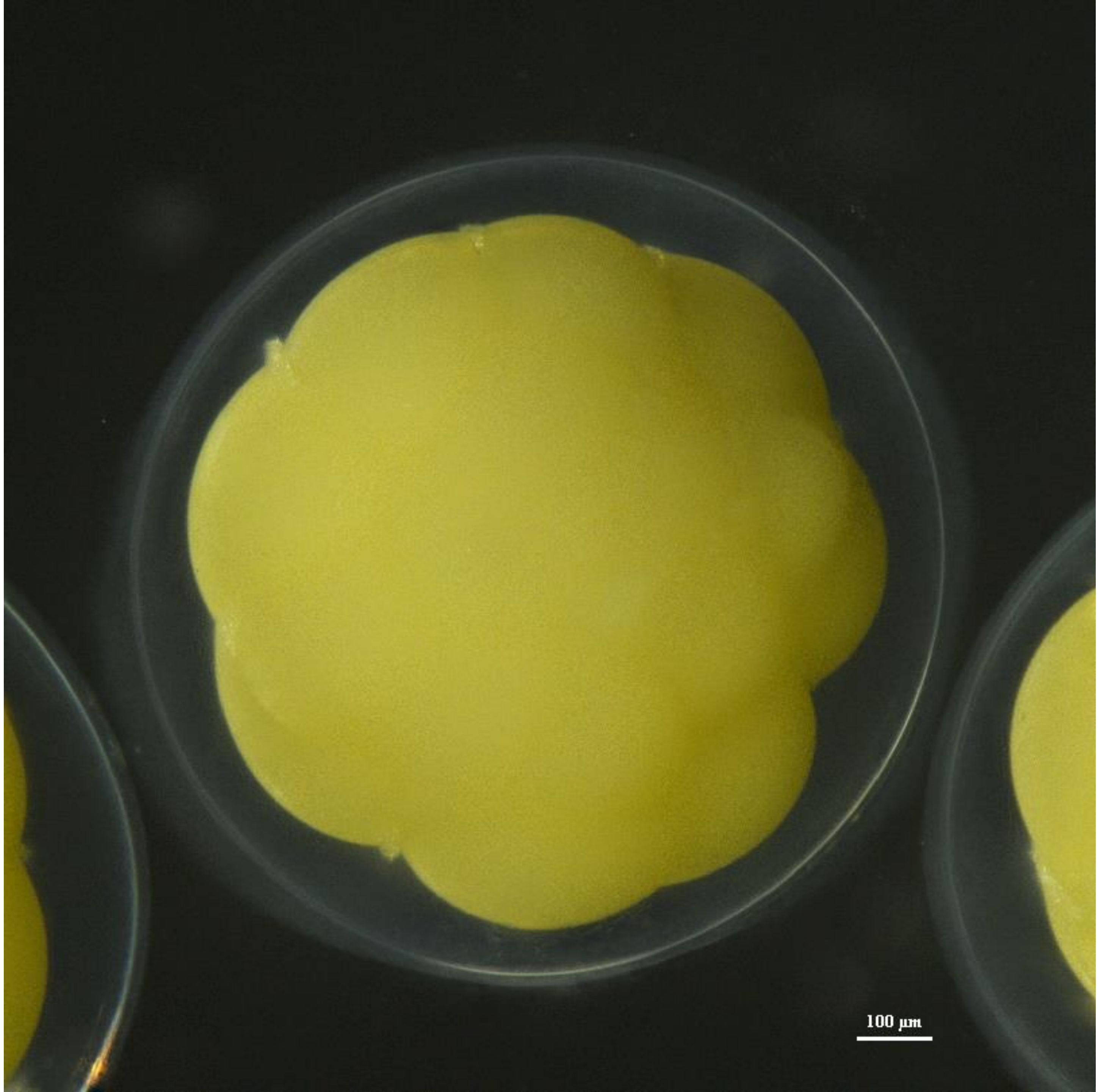

Supplementary Figure S3, continued

Figure7c（mimics）

32-cell

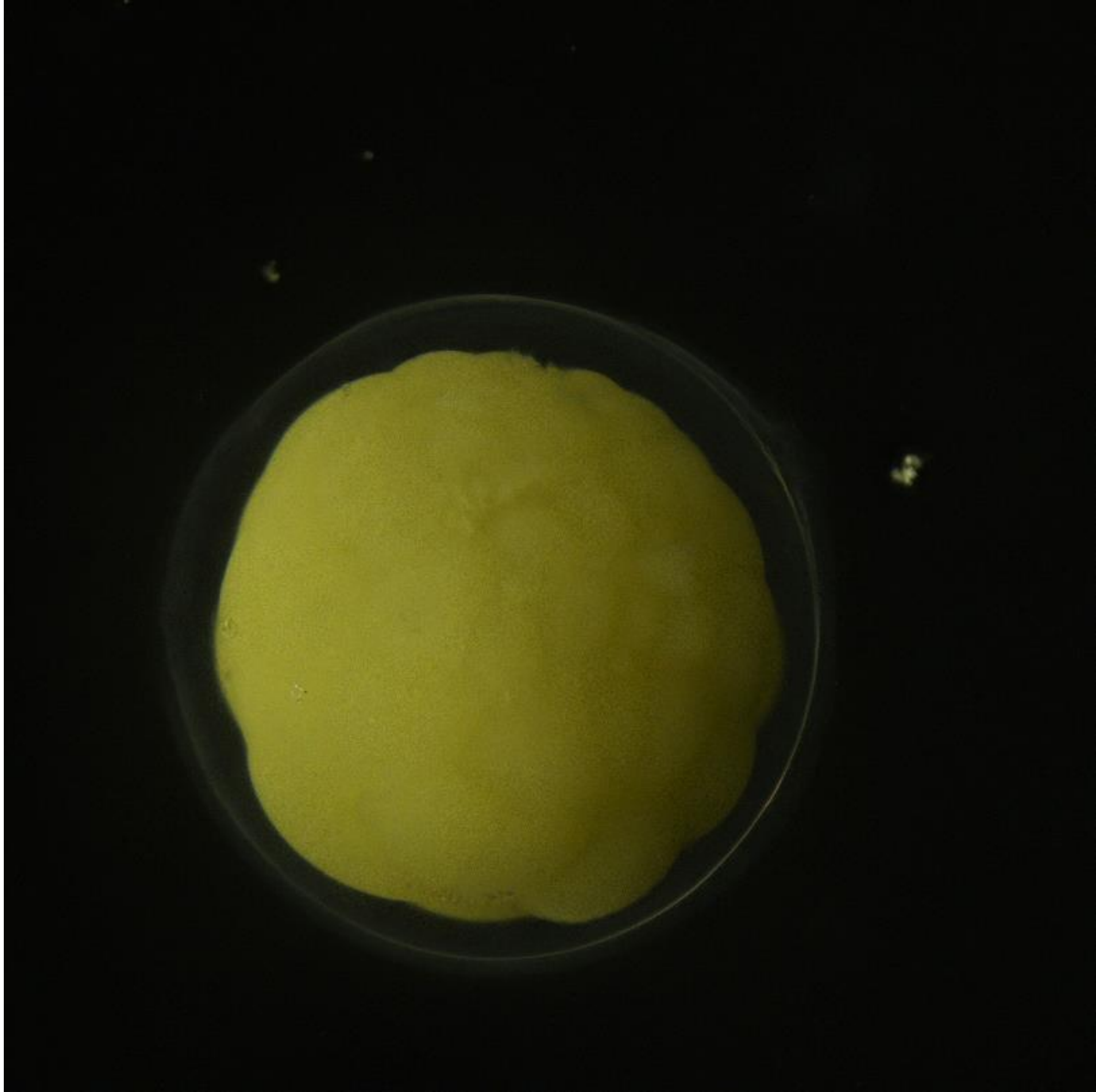

Supplementary Figure S3, continued

Figure7c（mimics）

64-cell

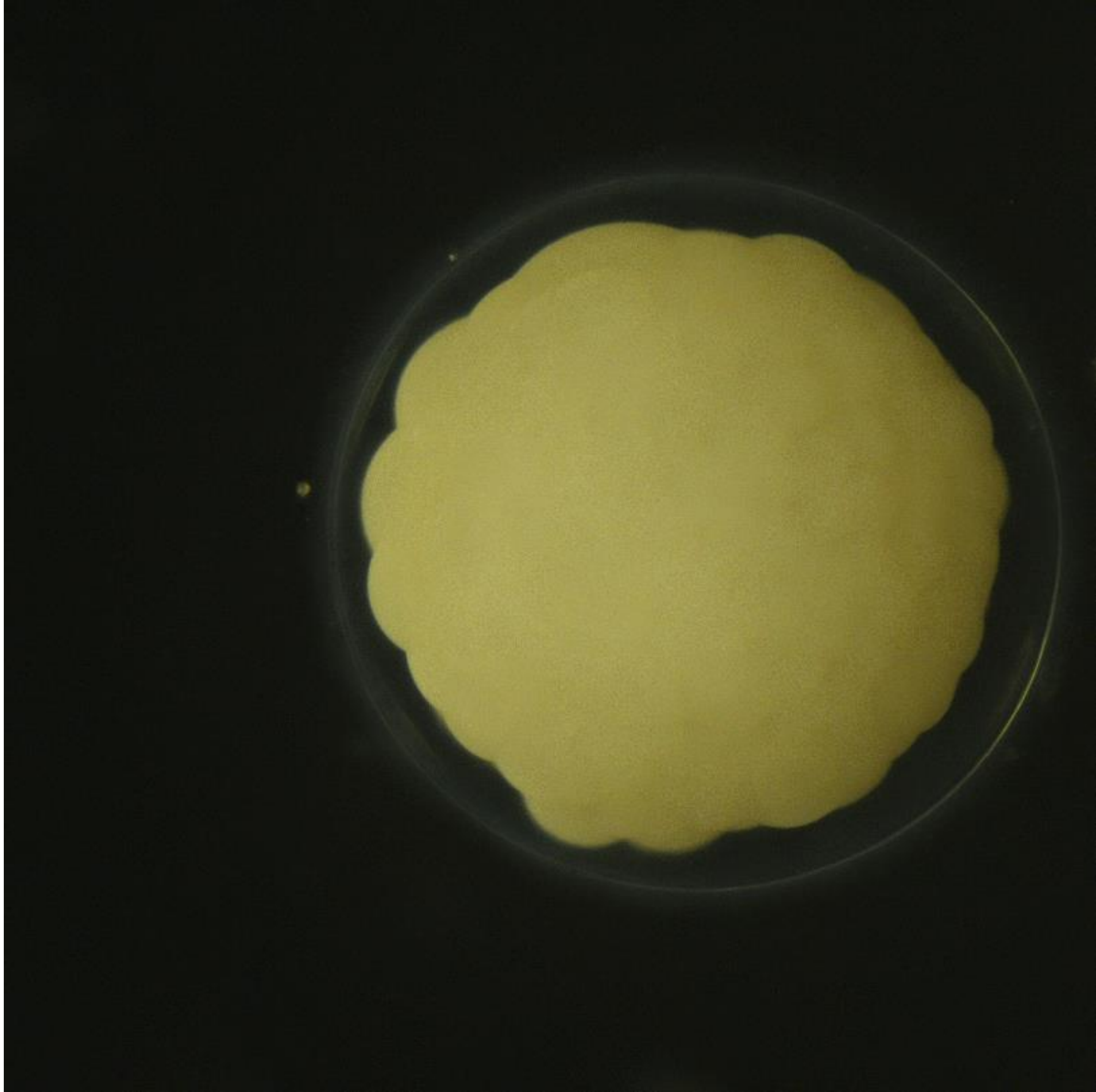

Supplementary Figure S3, continued

Figure7c（mimics）

128-cell

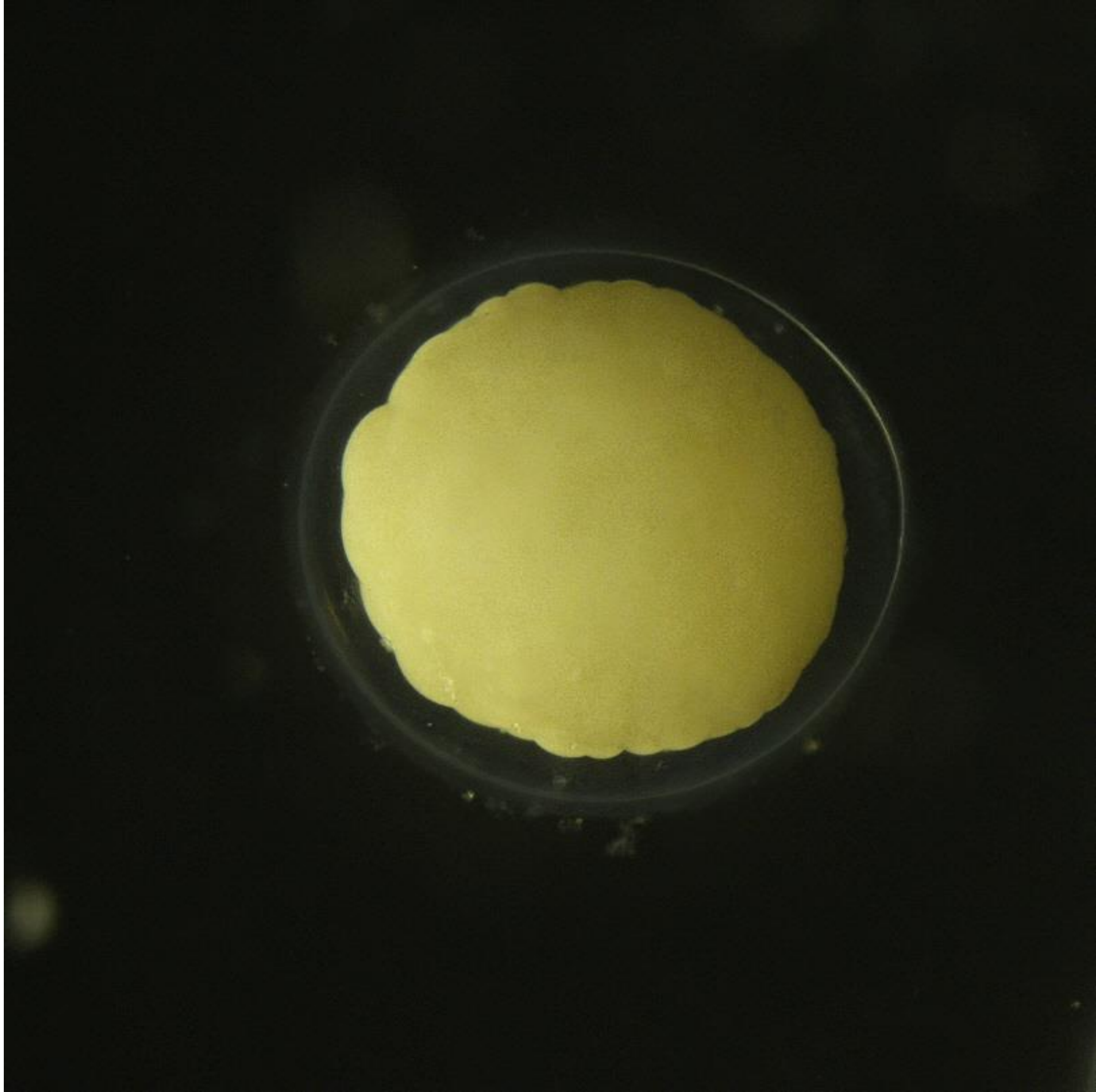

Supplementary Figure S3, continued

Figure7c（inhibitor）

zygote

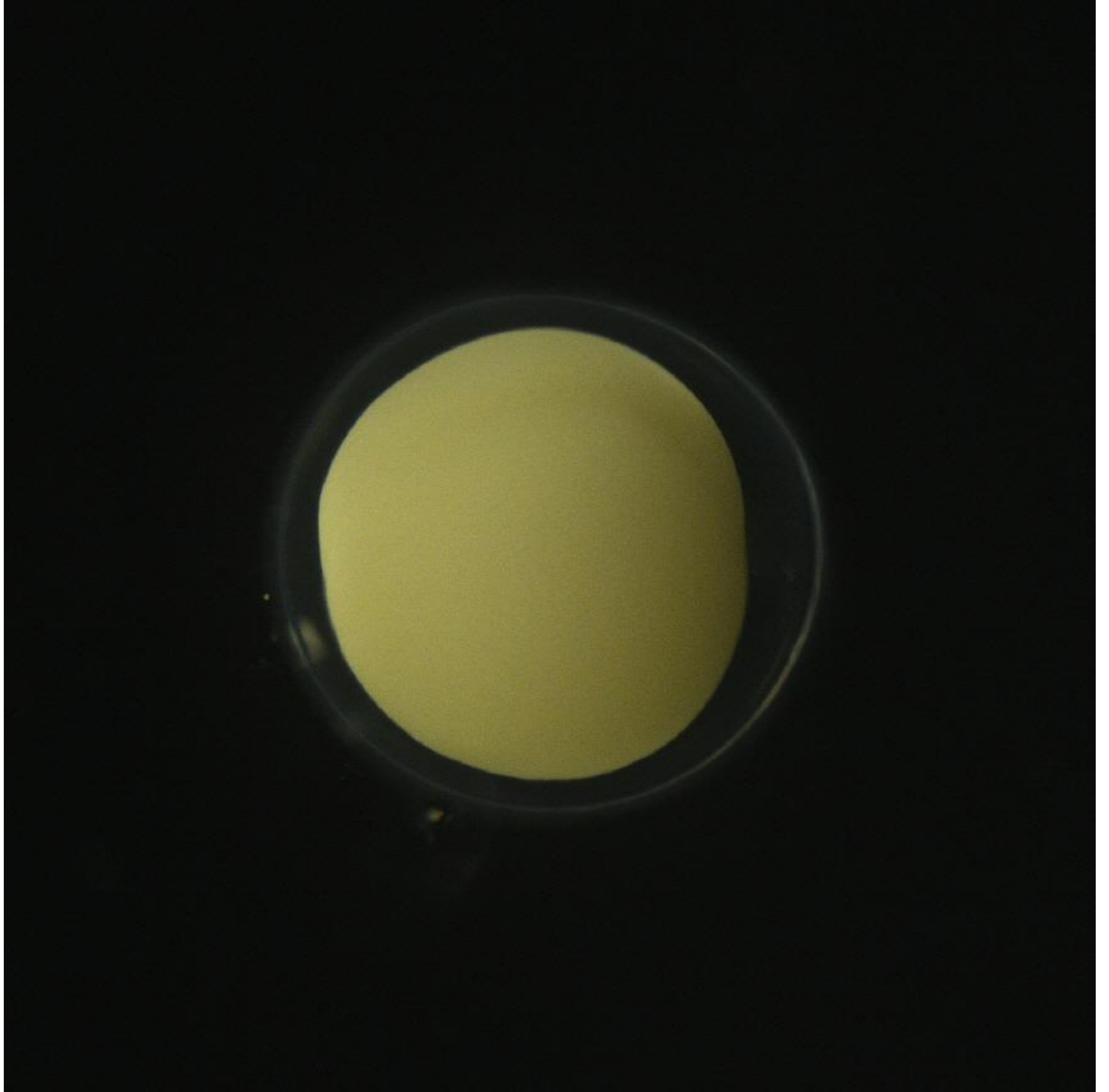

Supplementary Figure S3, continued

Figure7c（inhibitor）

2-cell

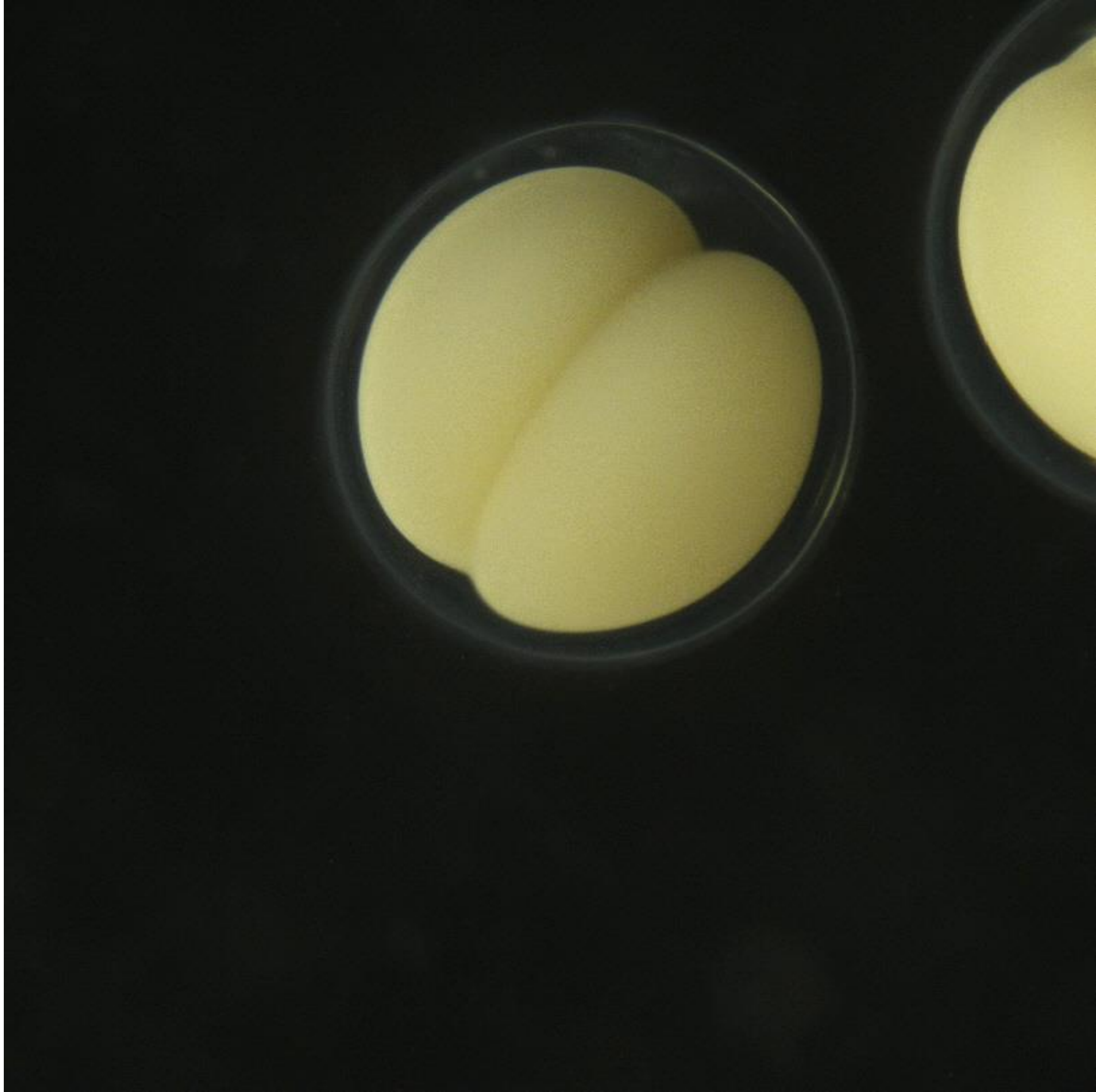

Supplementary Figure S3, continued

Figure7c（inhibitor）

4-cell

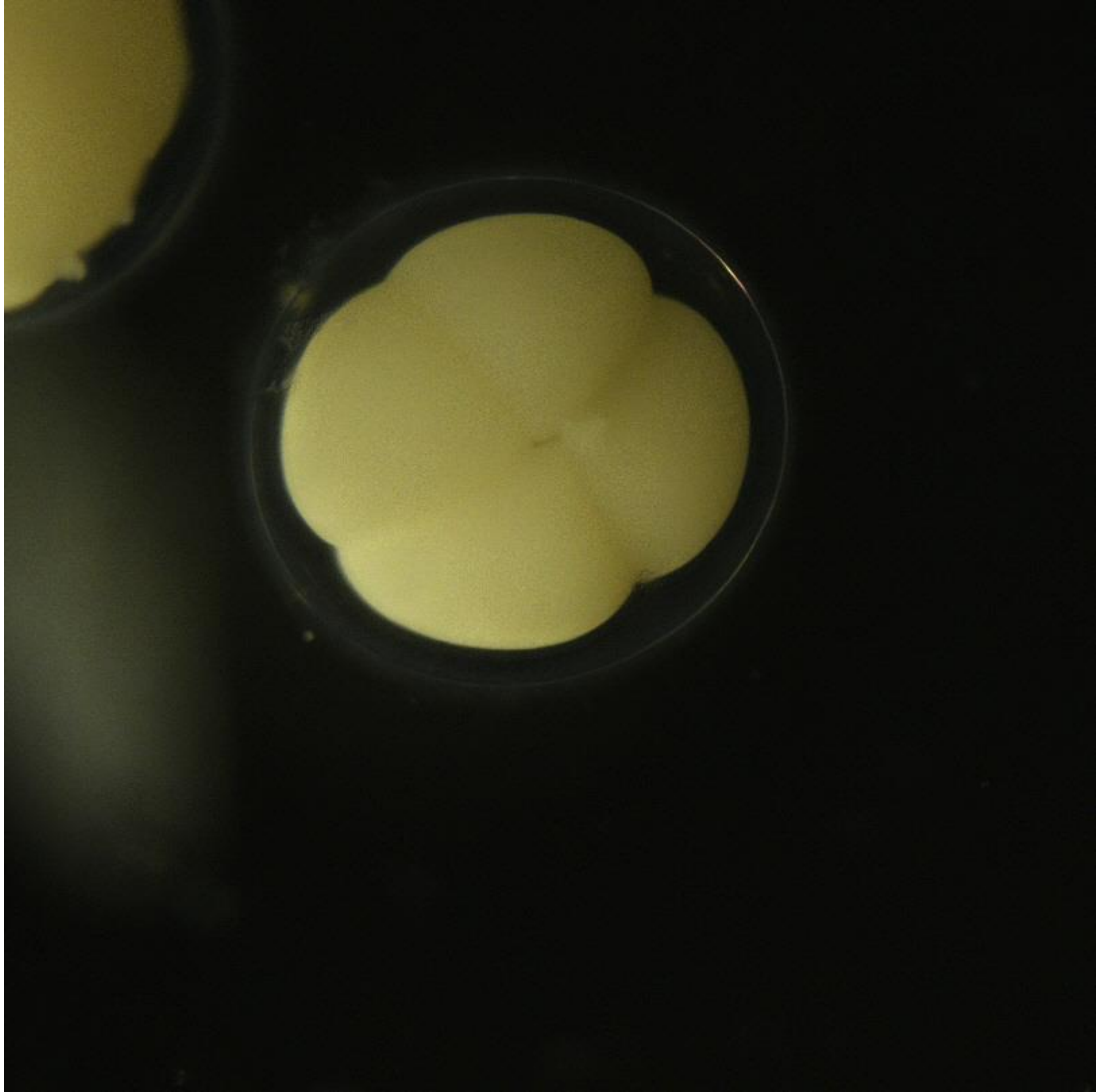

Supplementary Figure S3, continued

Figure7c（inhibitor）

8-cell

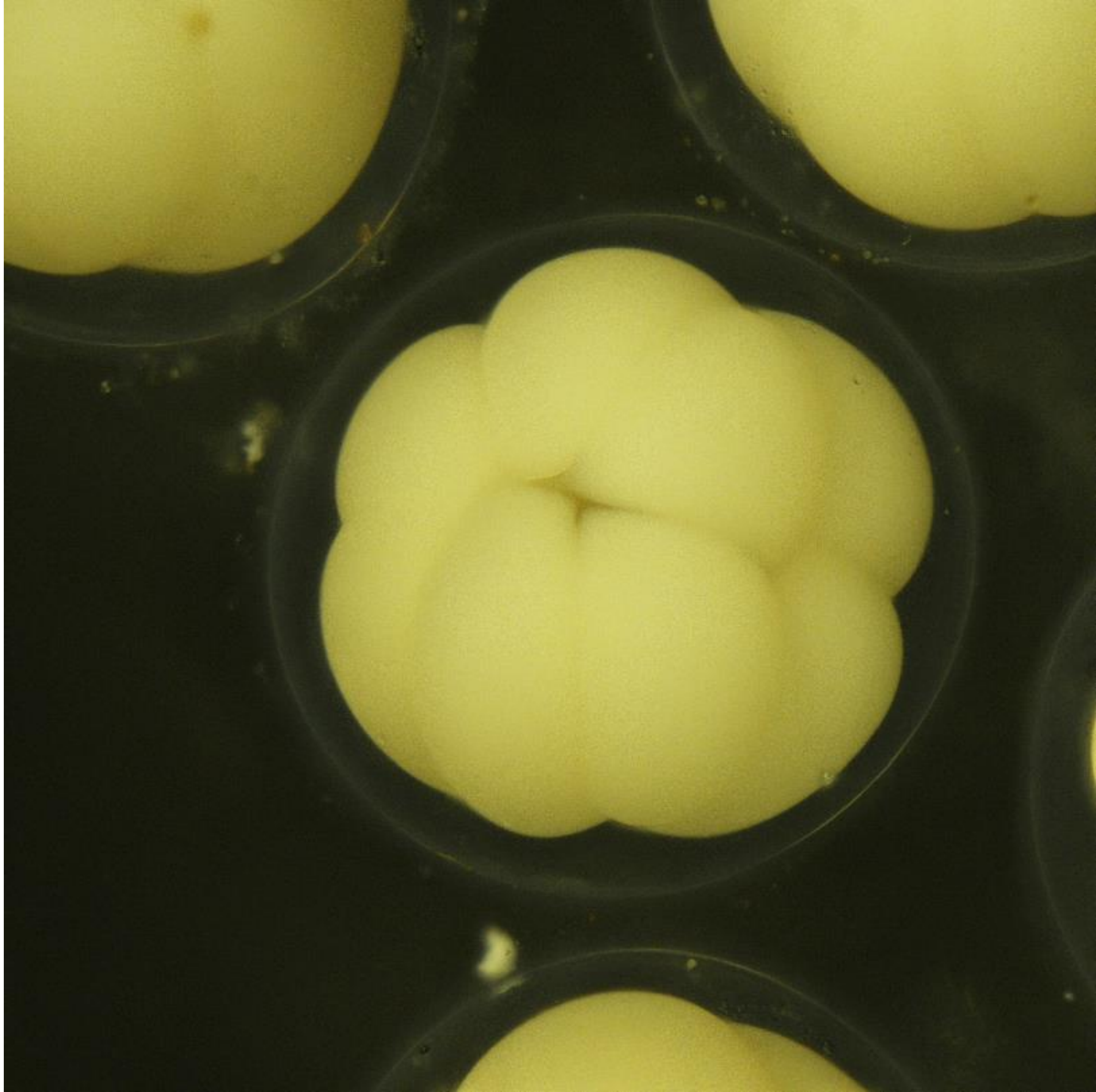

Supplementary Figure S3, continued

Figure7c（inhibitor）

16-cell

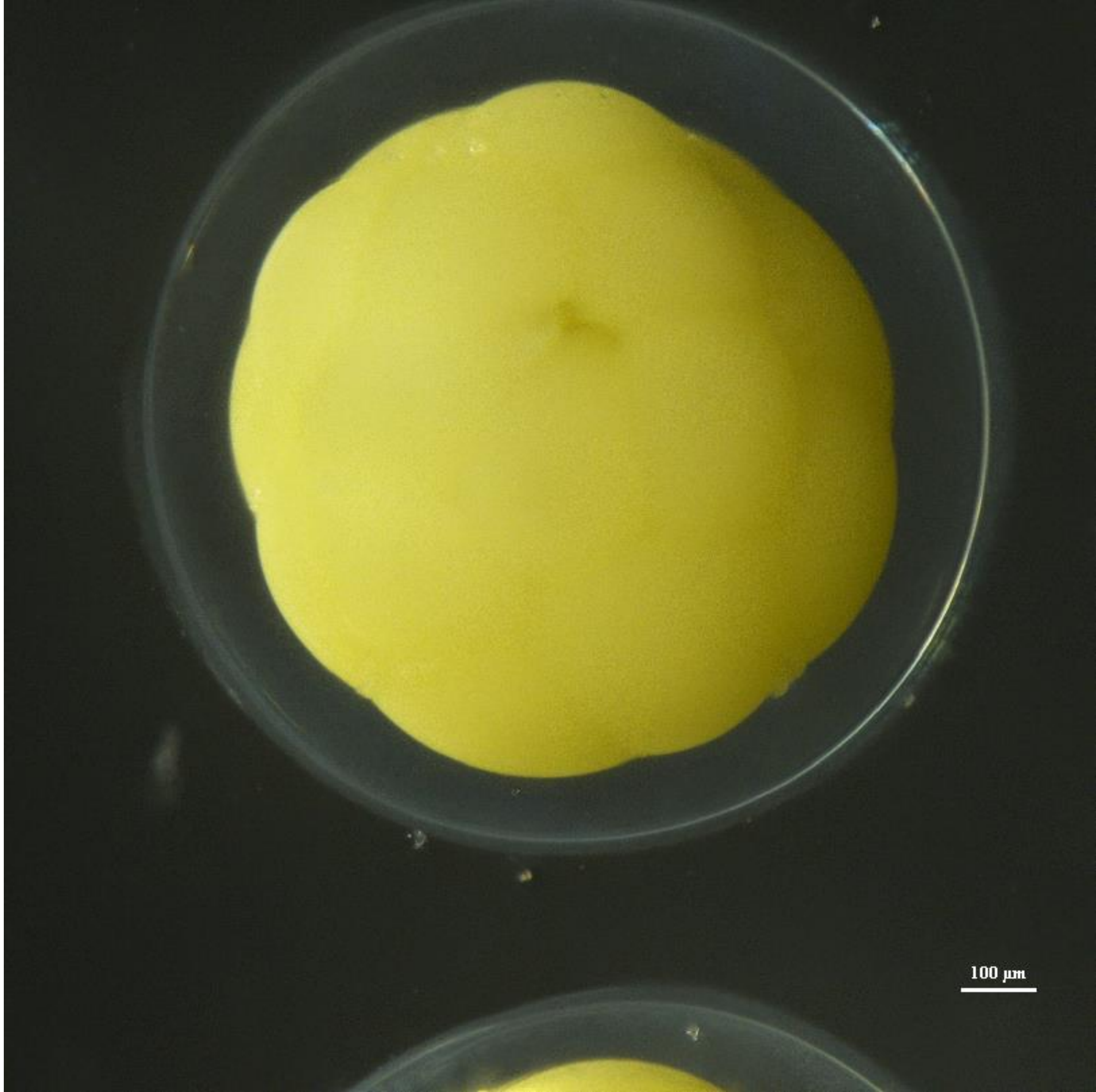

Supplementary Figure S3, continued

Figure7c（inhibitor）

32-cell

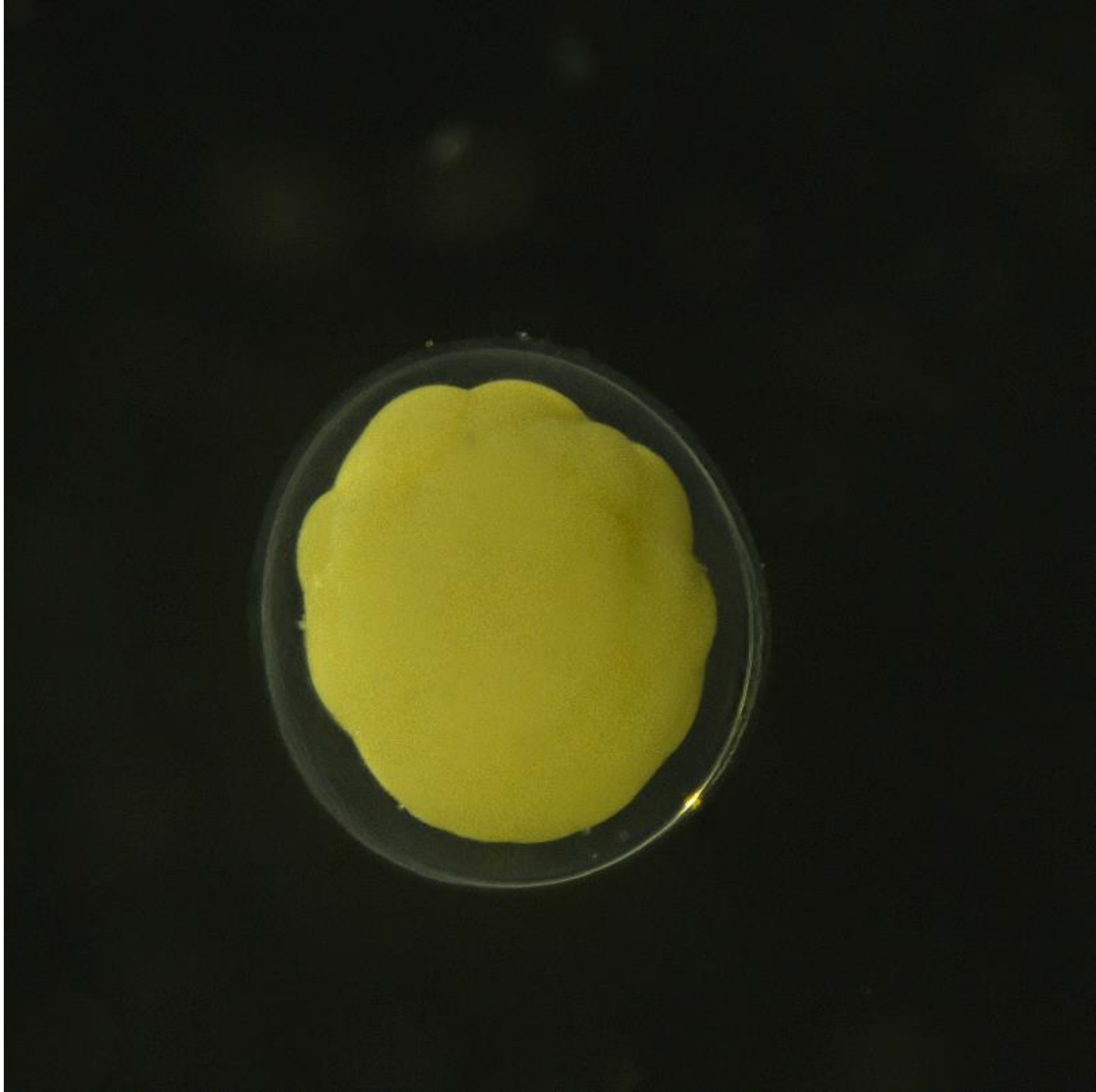

Supplementary Figure S3, continued

Figure7c（inhibitor）

64-cell

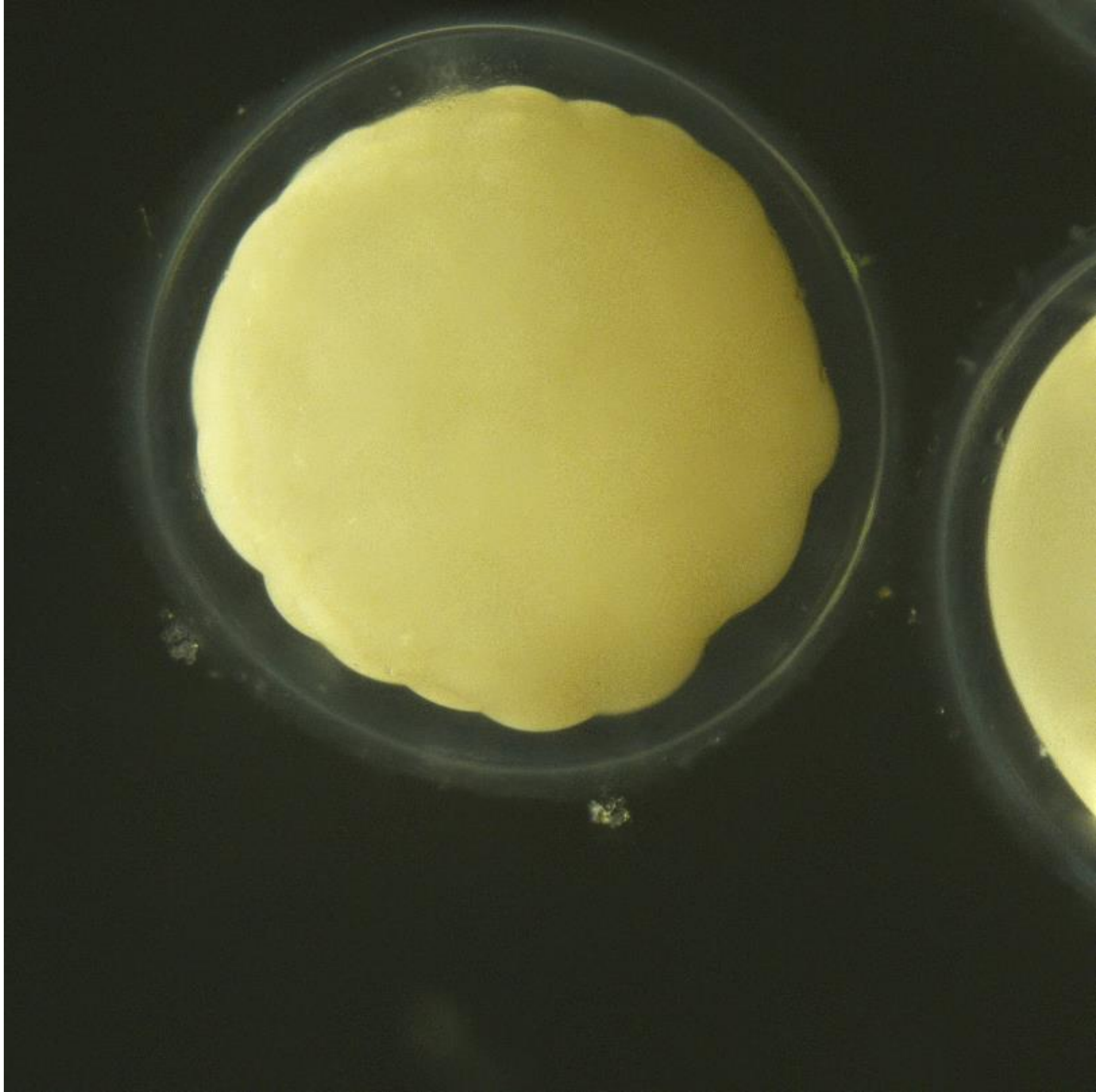

Supplementary Figure S3, continued

Figure7c（inhibitor）

128-cell

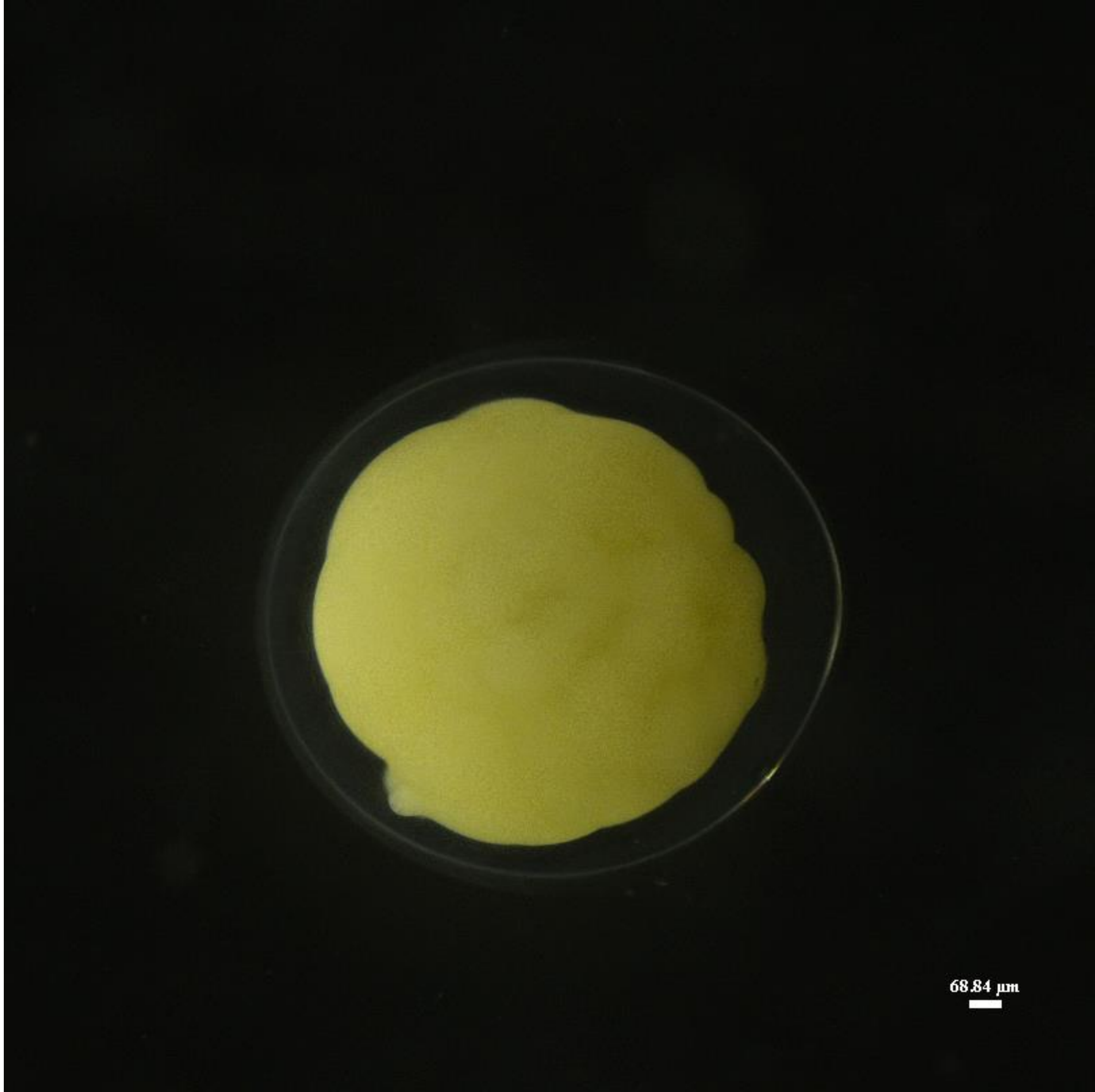

Supplementary Figure S3, continued

Figure7c（inhibitor）

256-cell

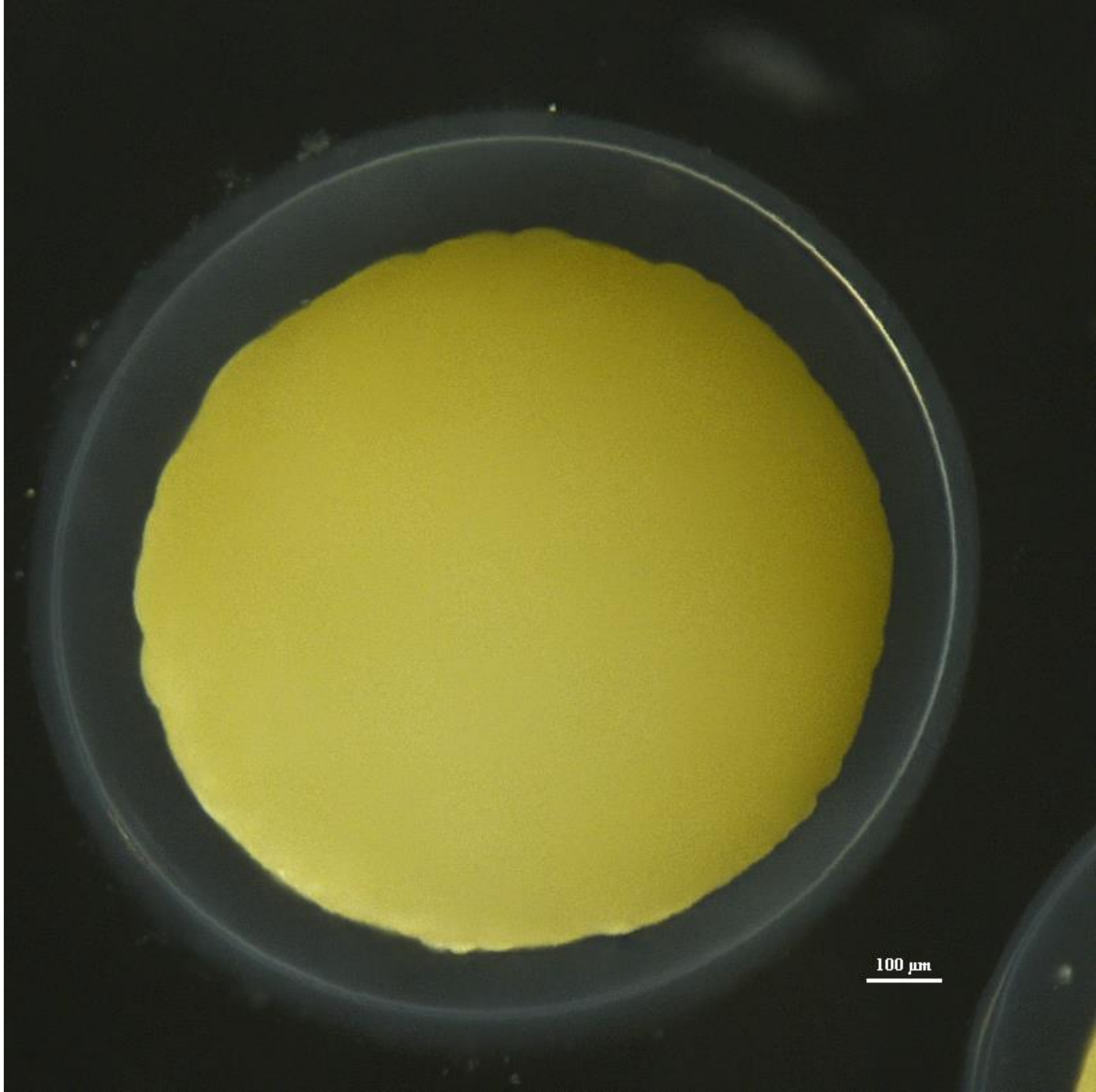

Supplementary Figure S3, continued

Figure7c（inhibitor）

gastrula

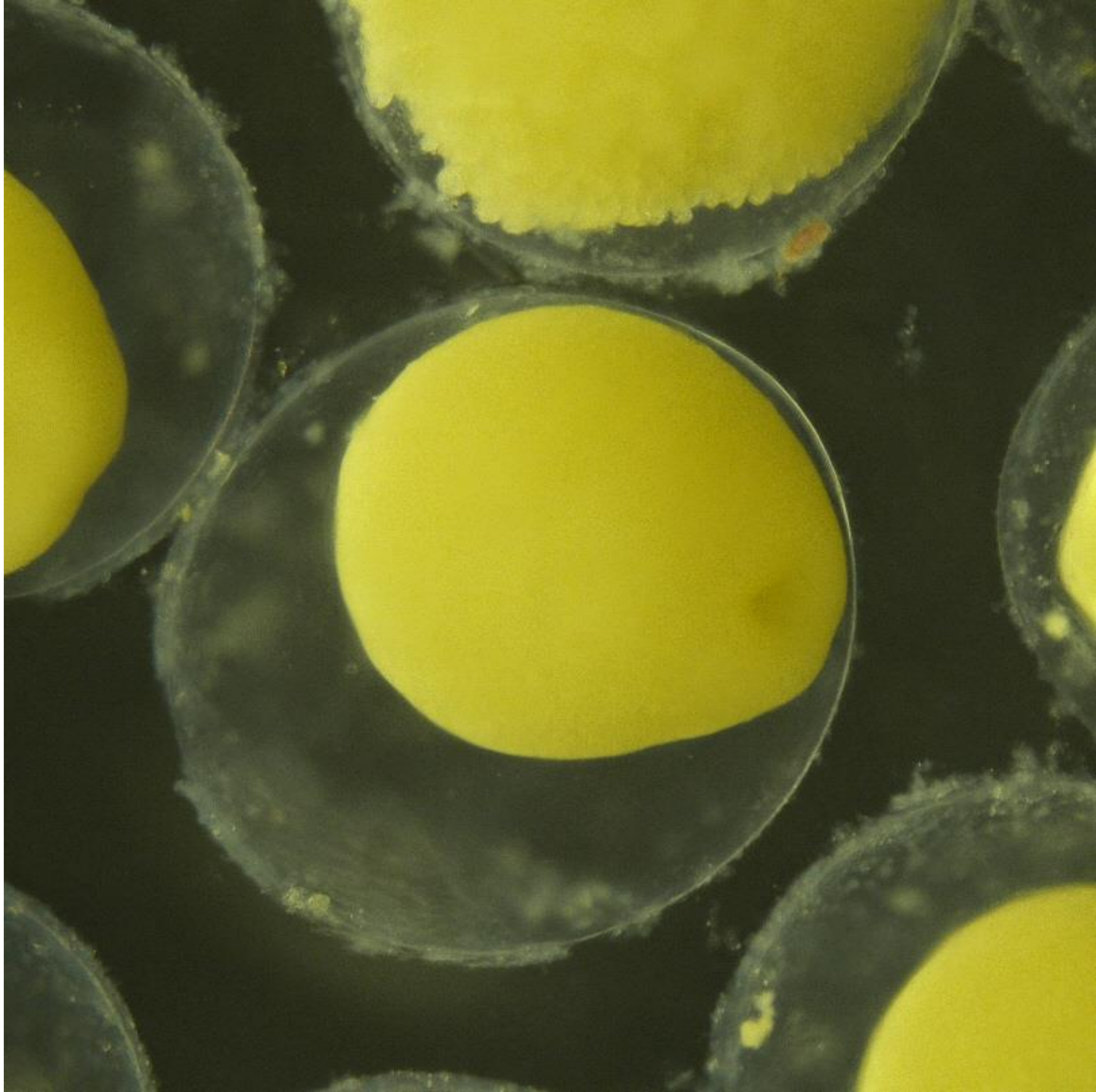

Supplementary Figure S3,
continued

Figure7c（inhibitor）

neurual

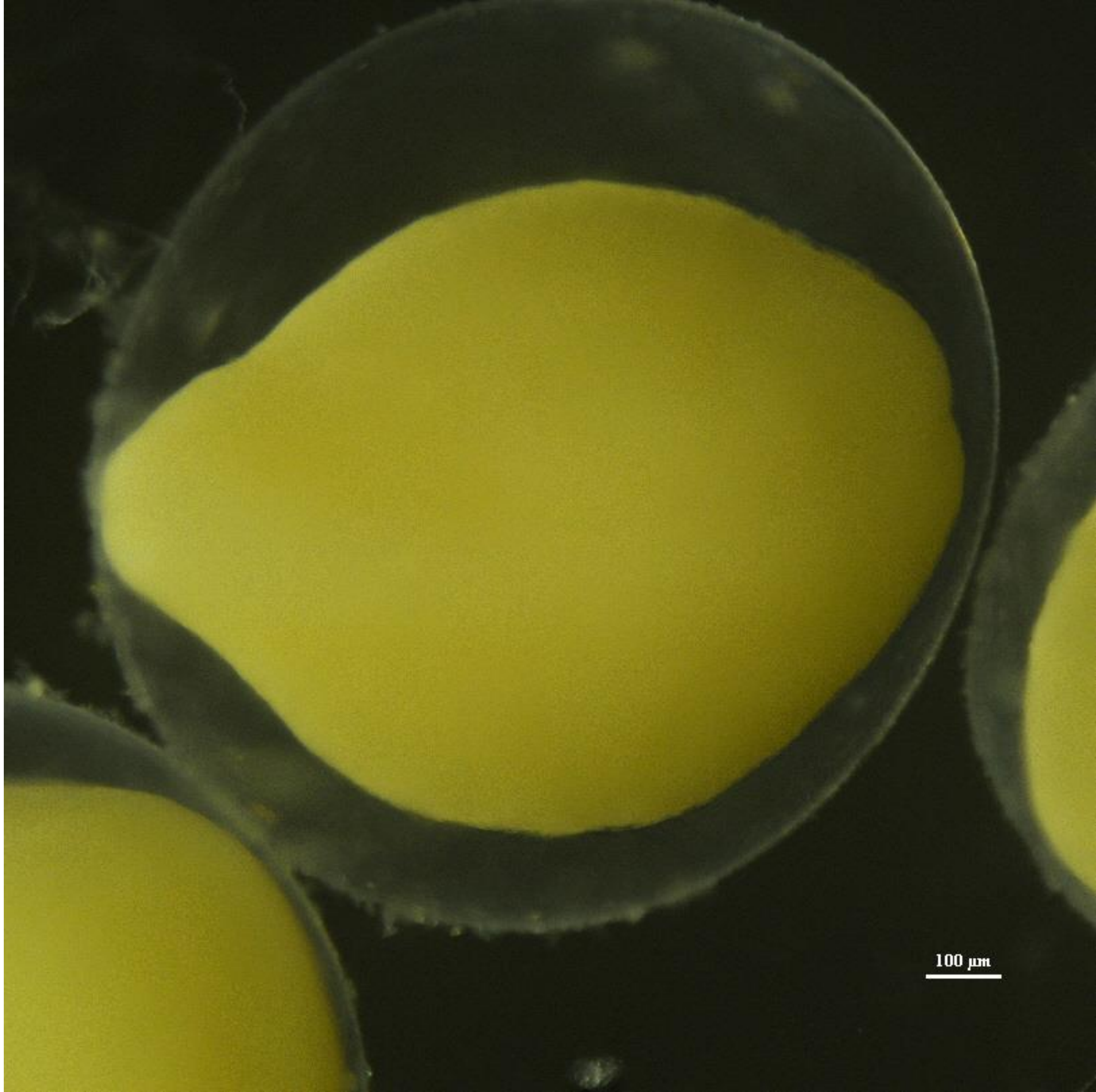

Supplementary Figure S3, continued

Figure7c（inhibitor）

head

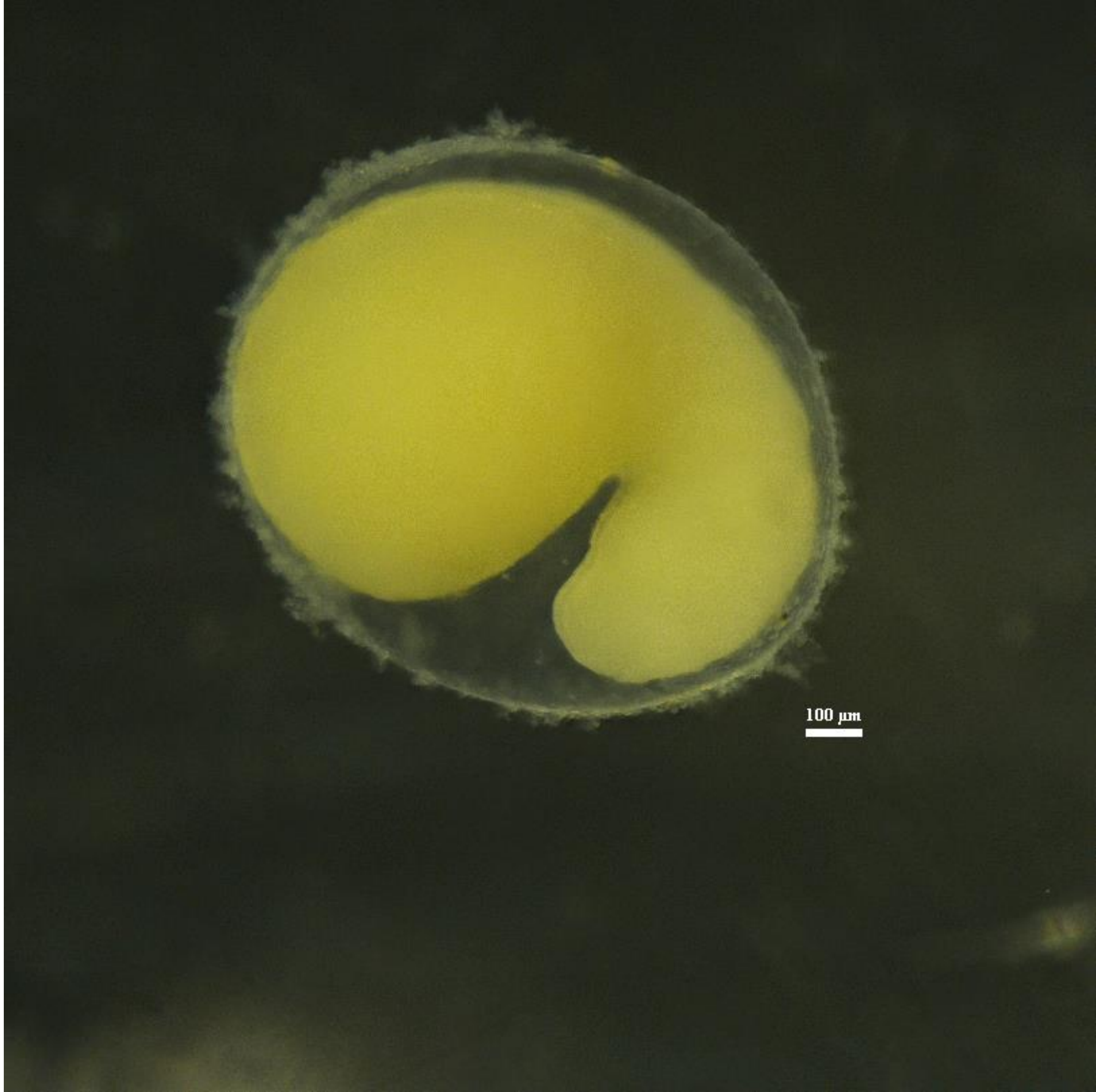

Supplementary Figure S3, continued

Figure7c（inhibitor）

prehatching

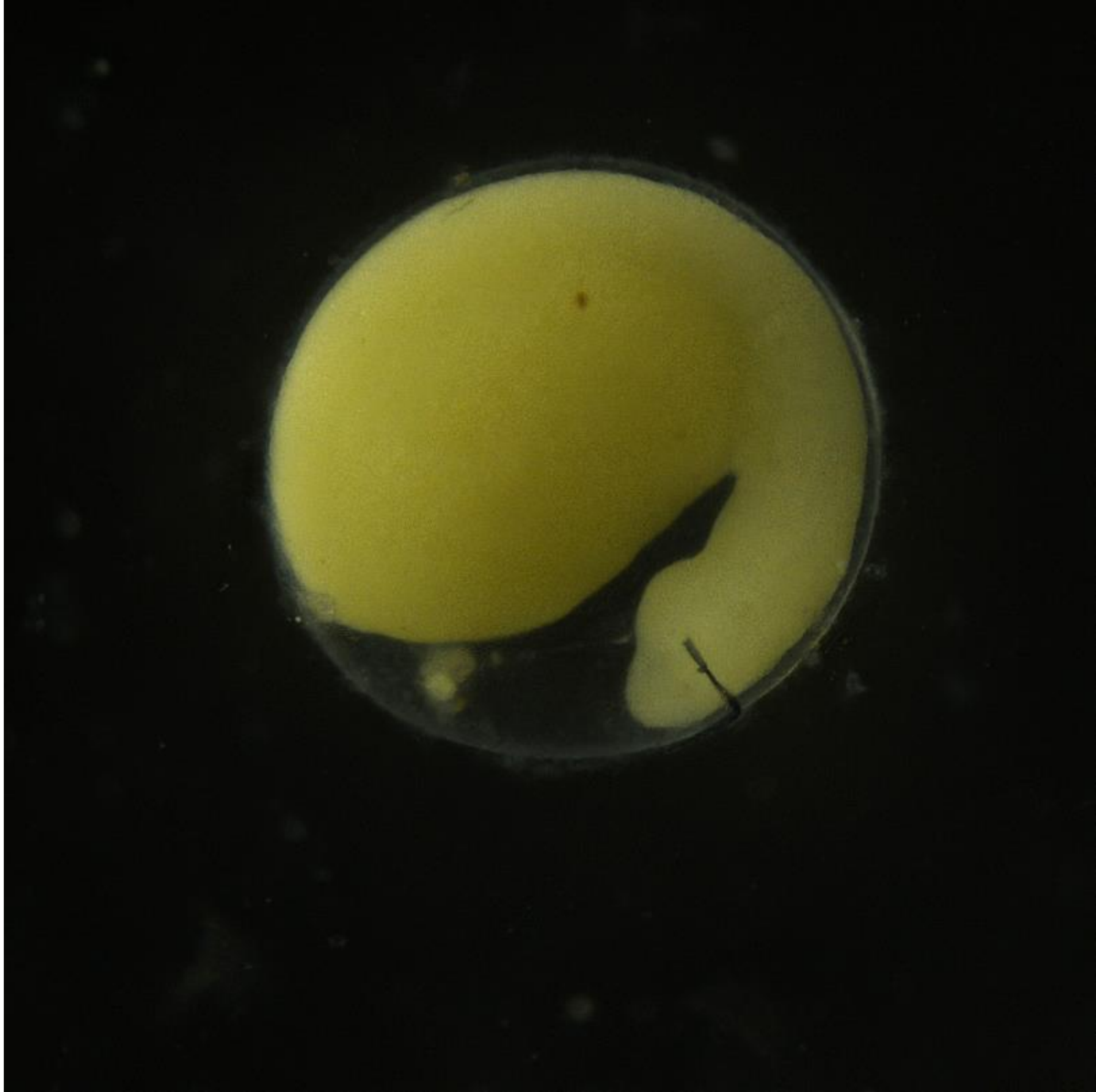

Supplementary Figure S3, continued

Figure7c（inhibitor）

hatching

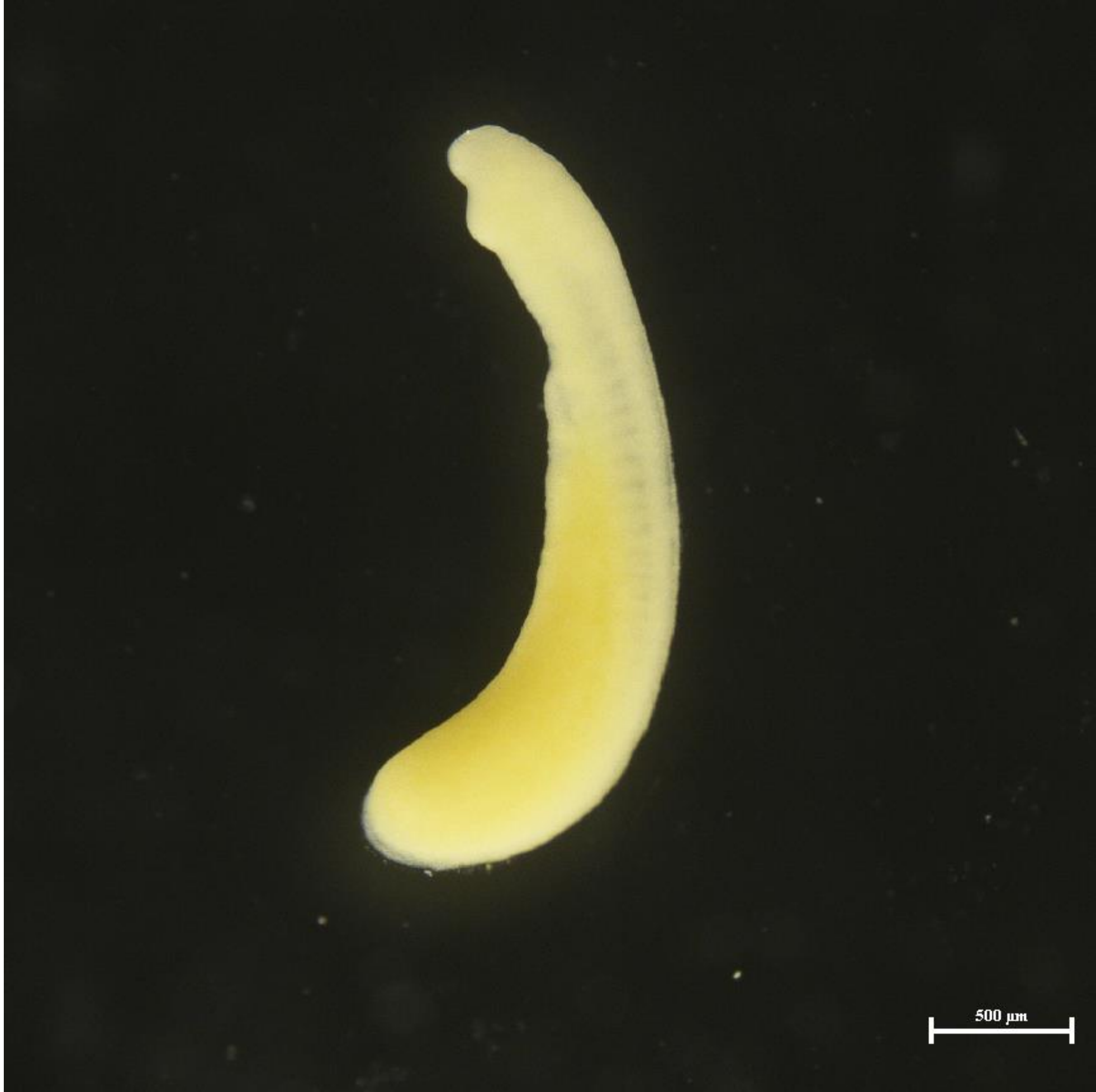

Supplementary Figure S3, continued

Figure7c（control）

zygote

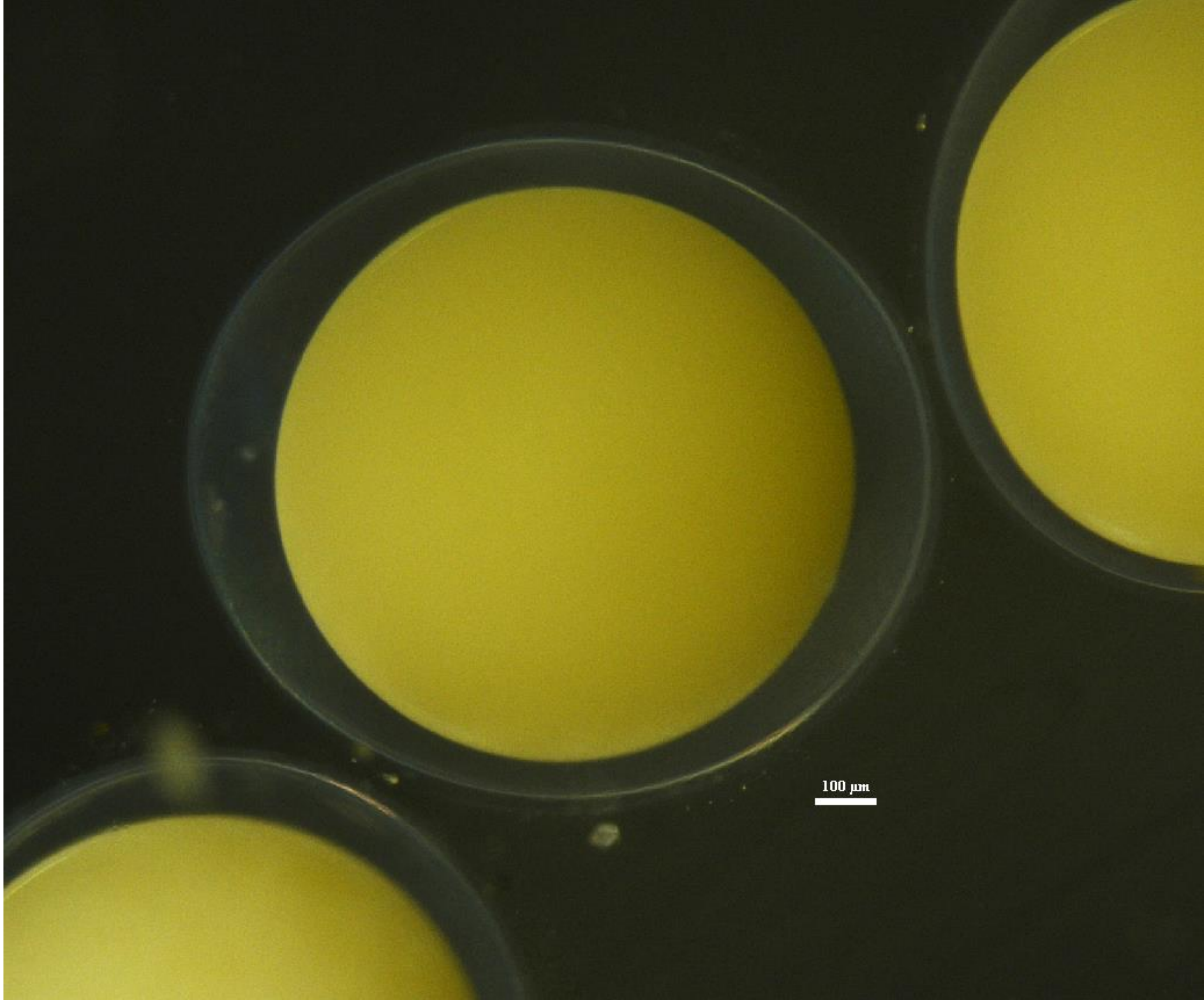

Supplementary Figure S3, continued

Figure7c（control）

2-cell

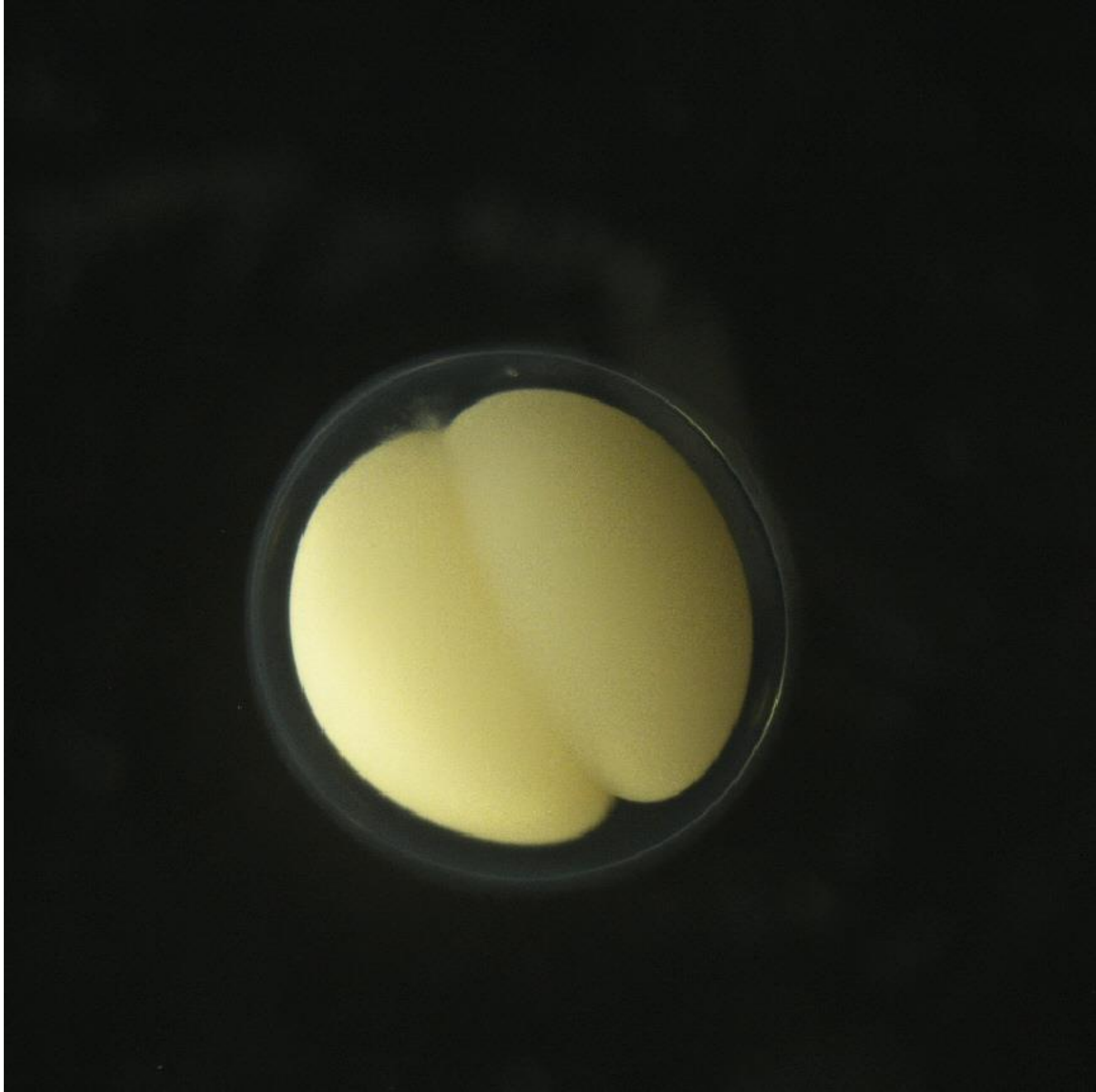

Supplementary Figure S3, continued

Figure7c（control）

4-cell

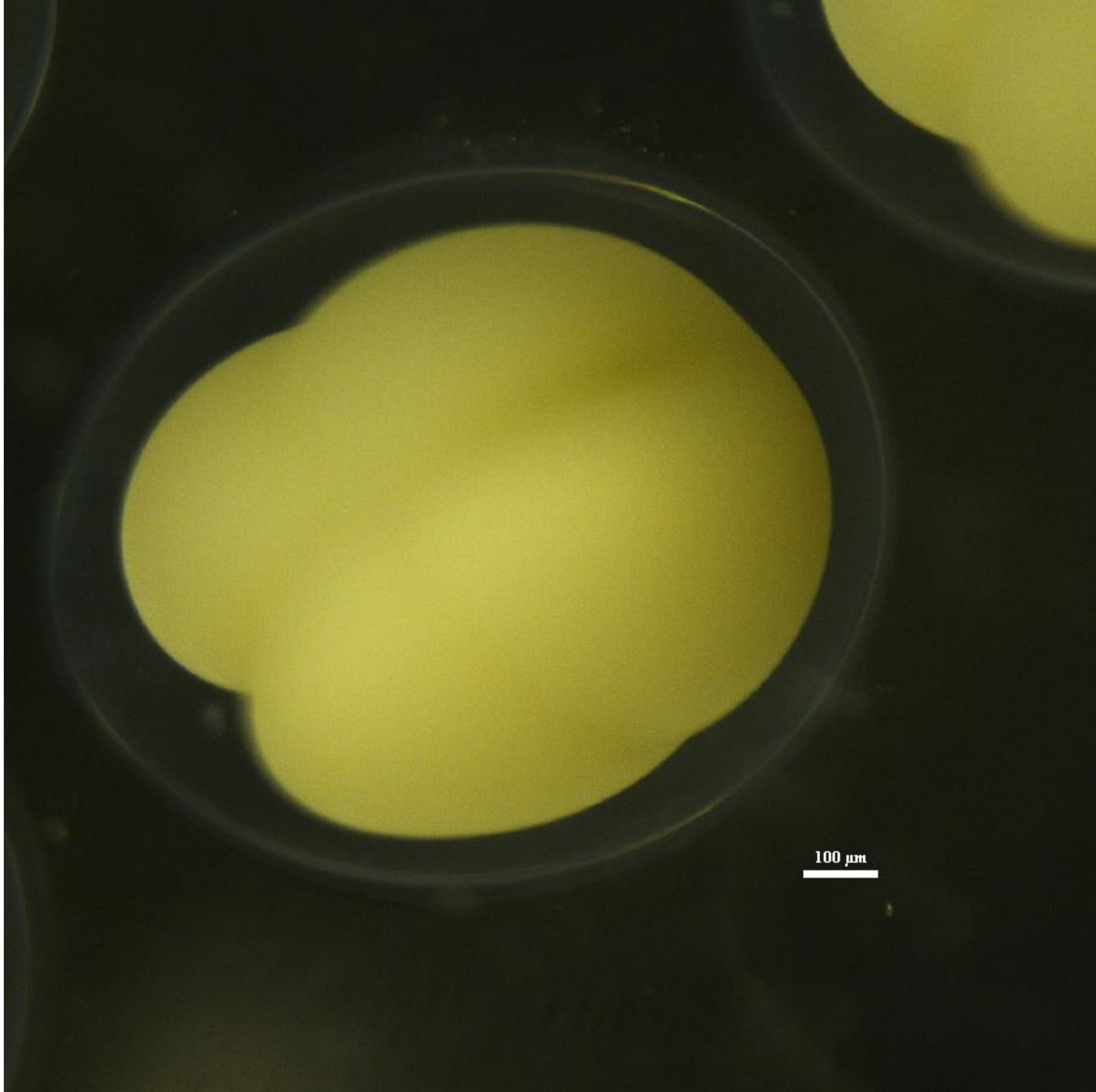

Supplementary Figure S3, continued

Figure7c（control）

8-cell

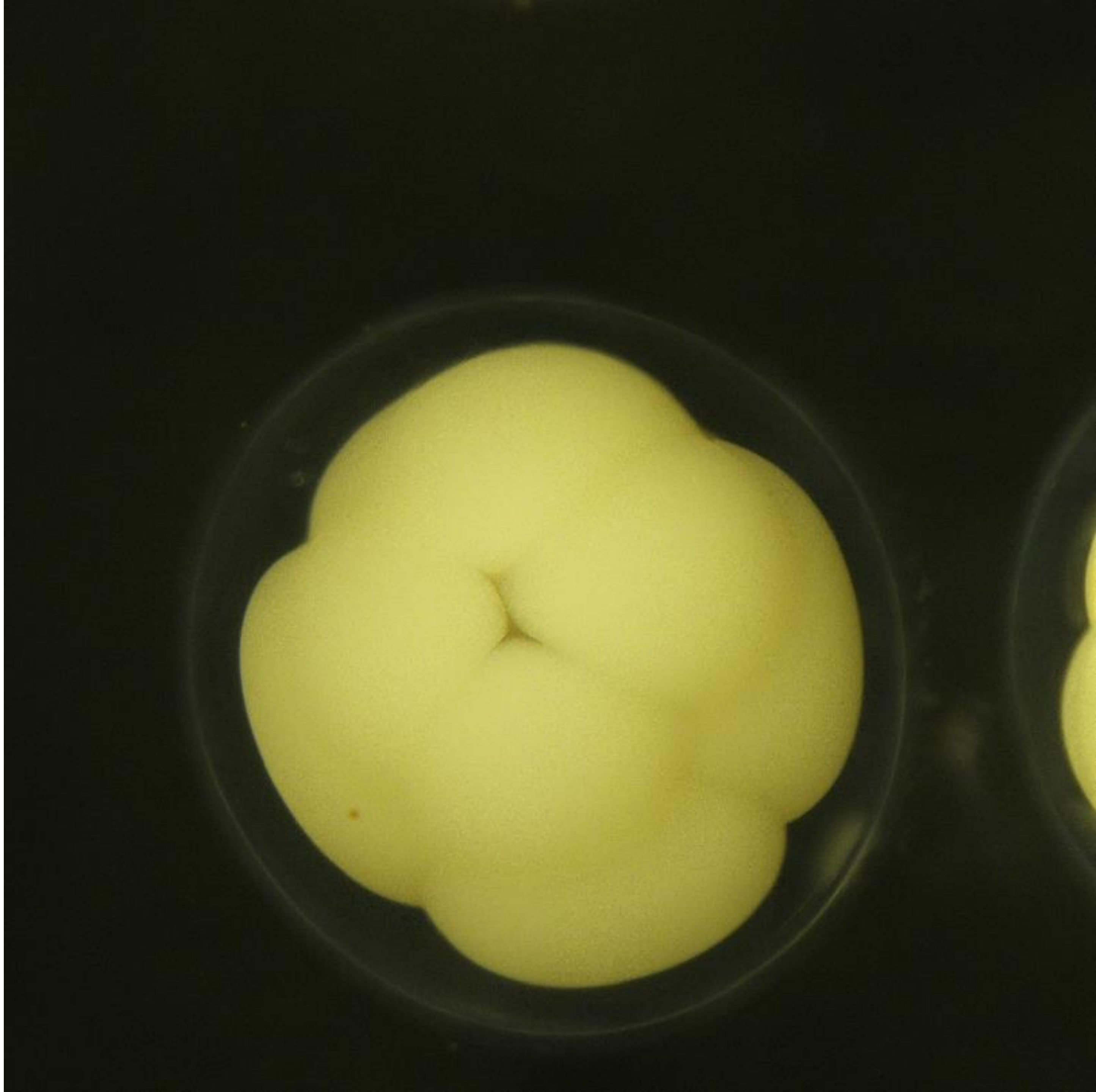

Supplementary Figure S3, continued

Figure7c（control）

16-cell

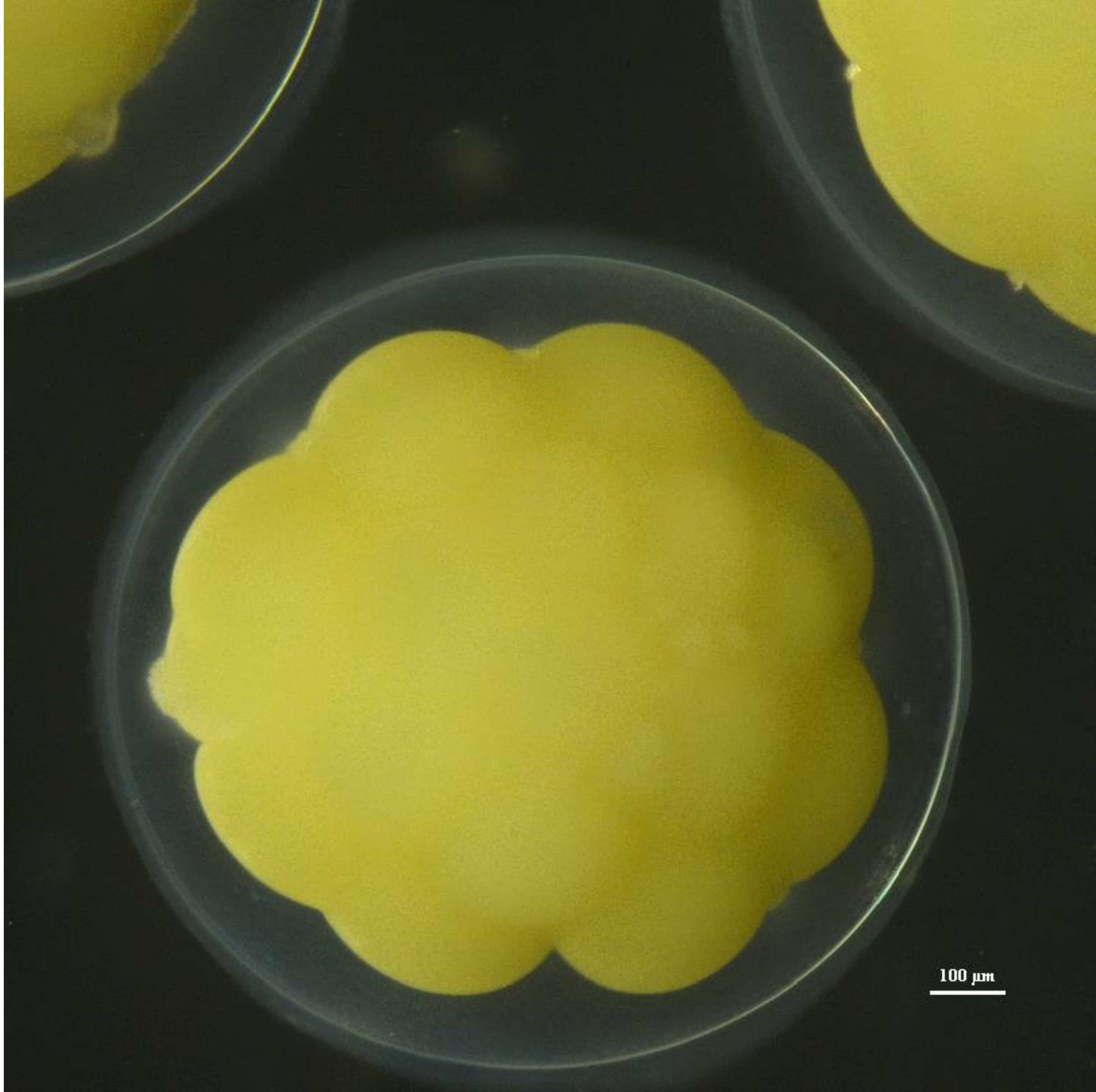

Supplementary Figure S3,
continued

Figure7c（control）

32-cell

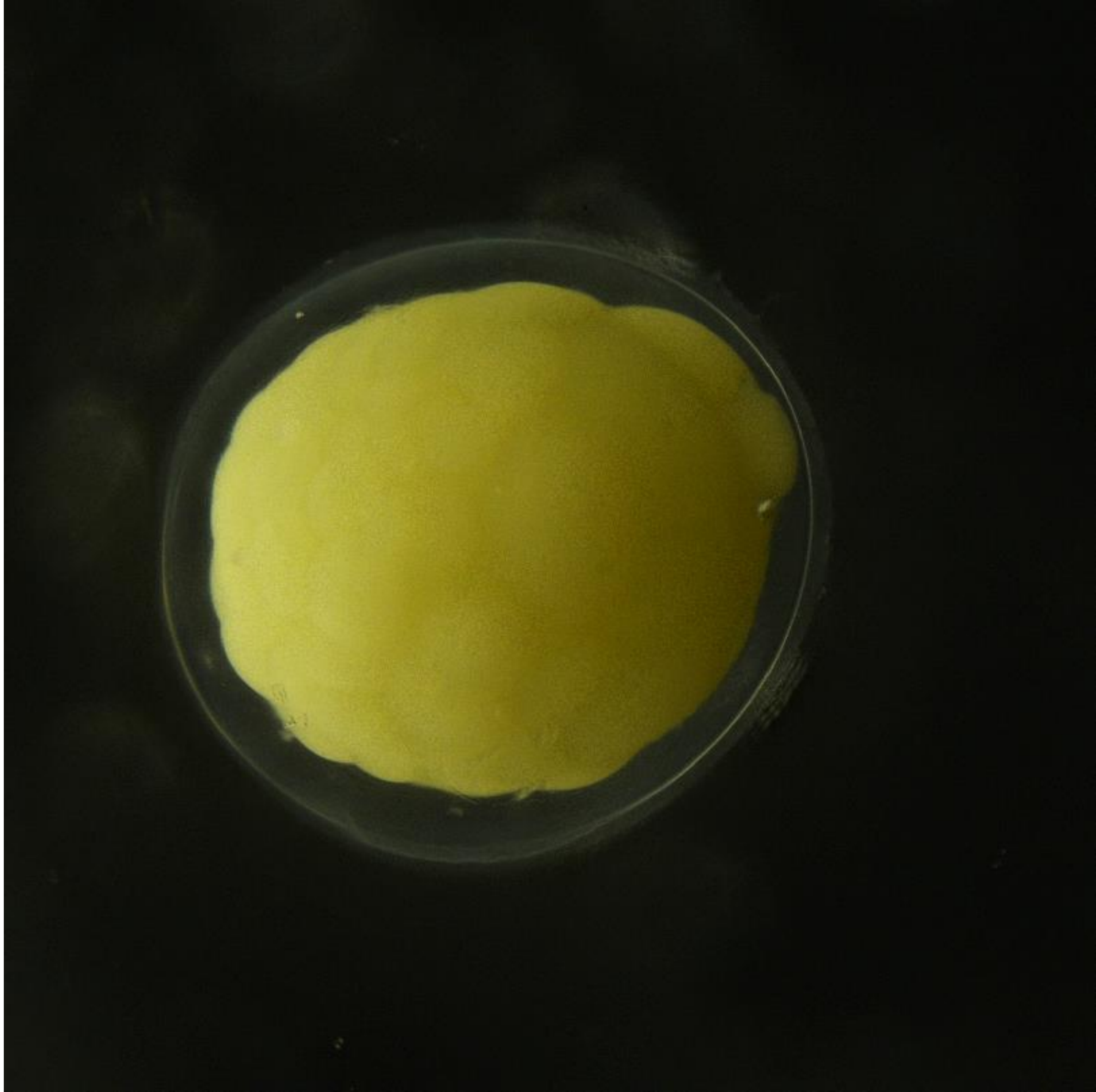

Supplementary Figure S3,
continued

Figure7c（control）

64-cell

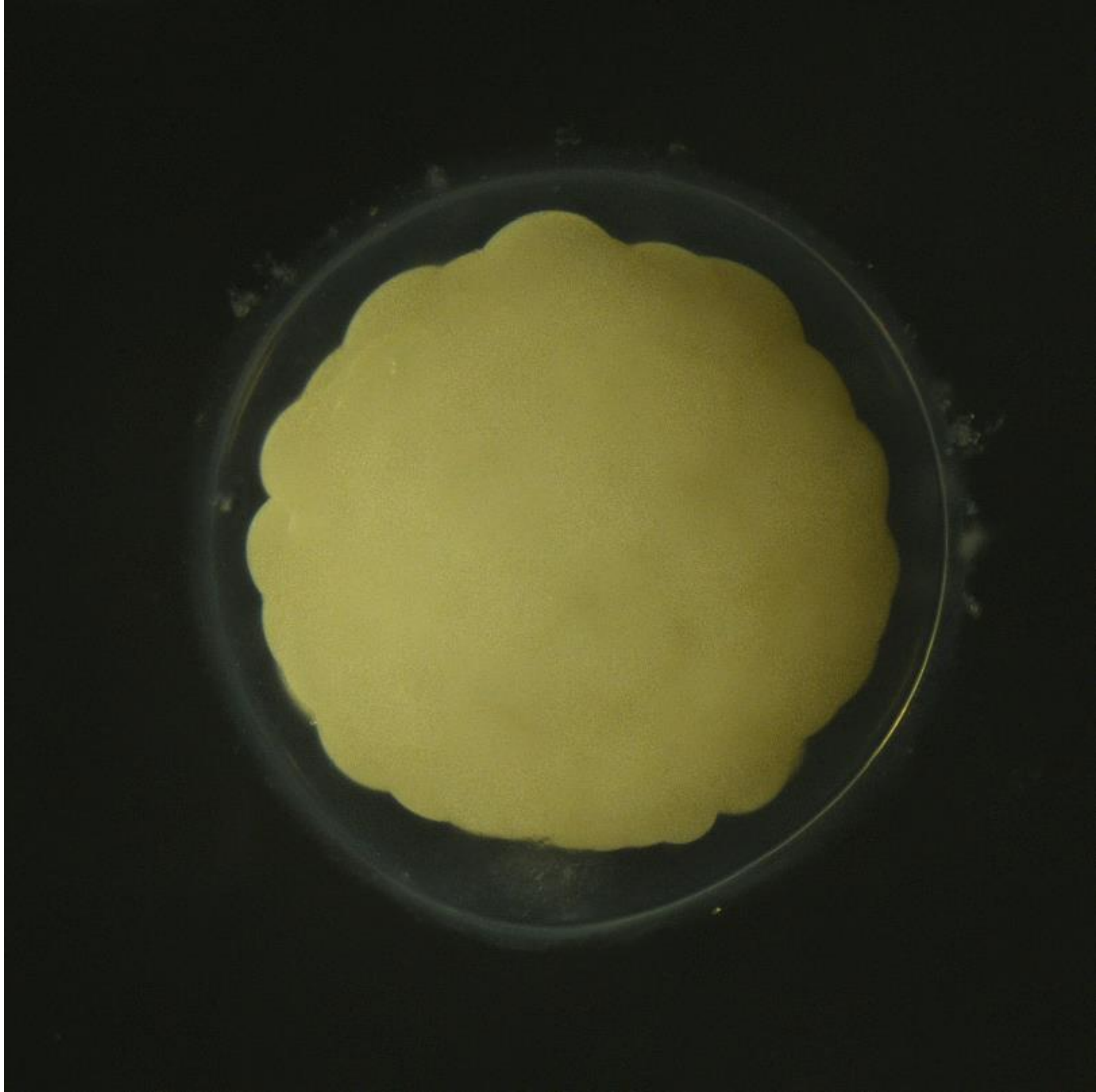

Supplementary Figure S3, continued

Figure7c（control）

128-cell

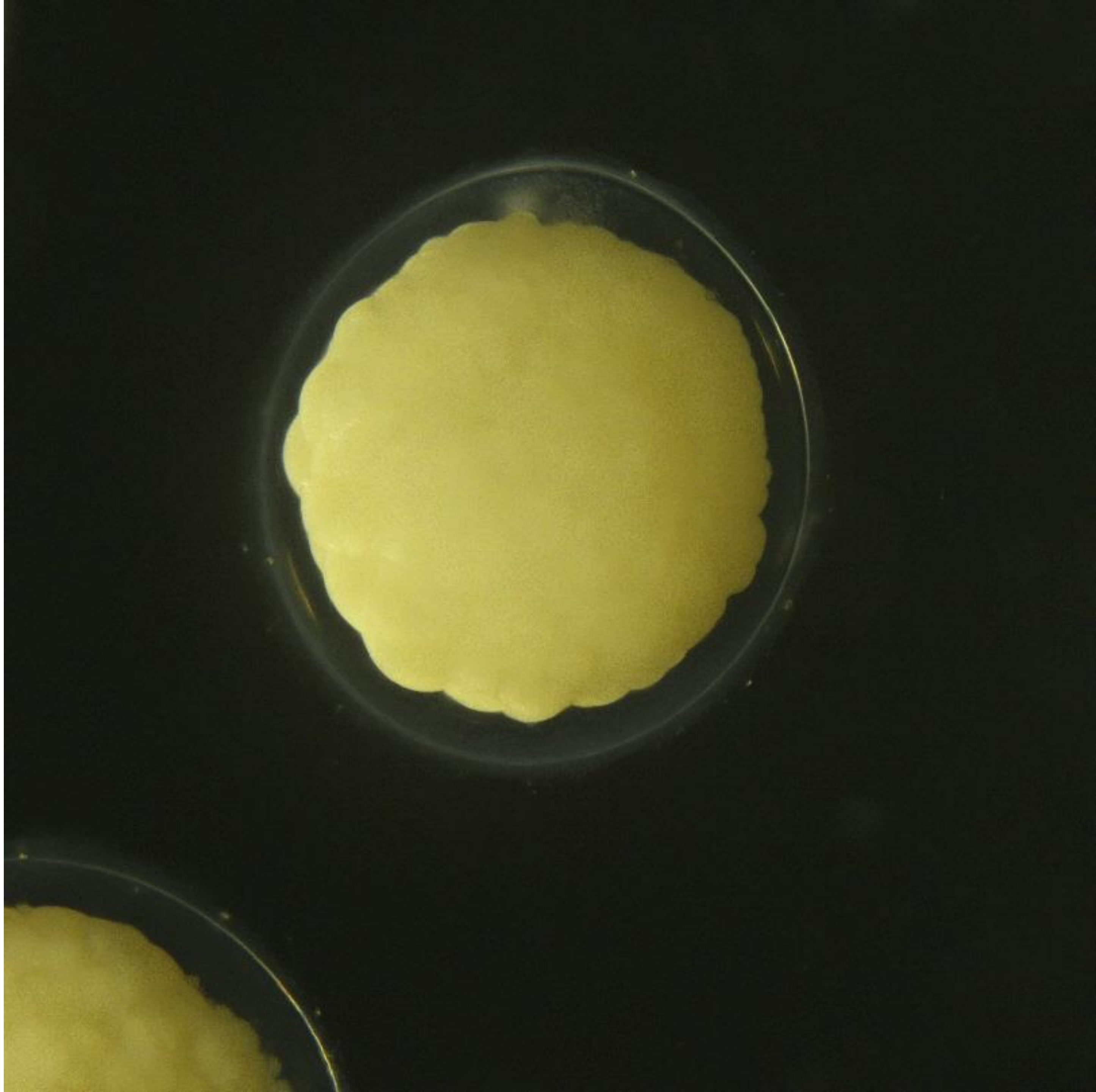

Supplementary Figure S3, continued

Figure7c（control）

256-cell

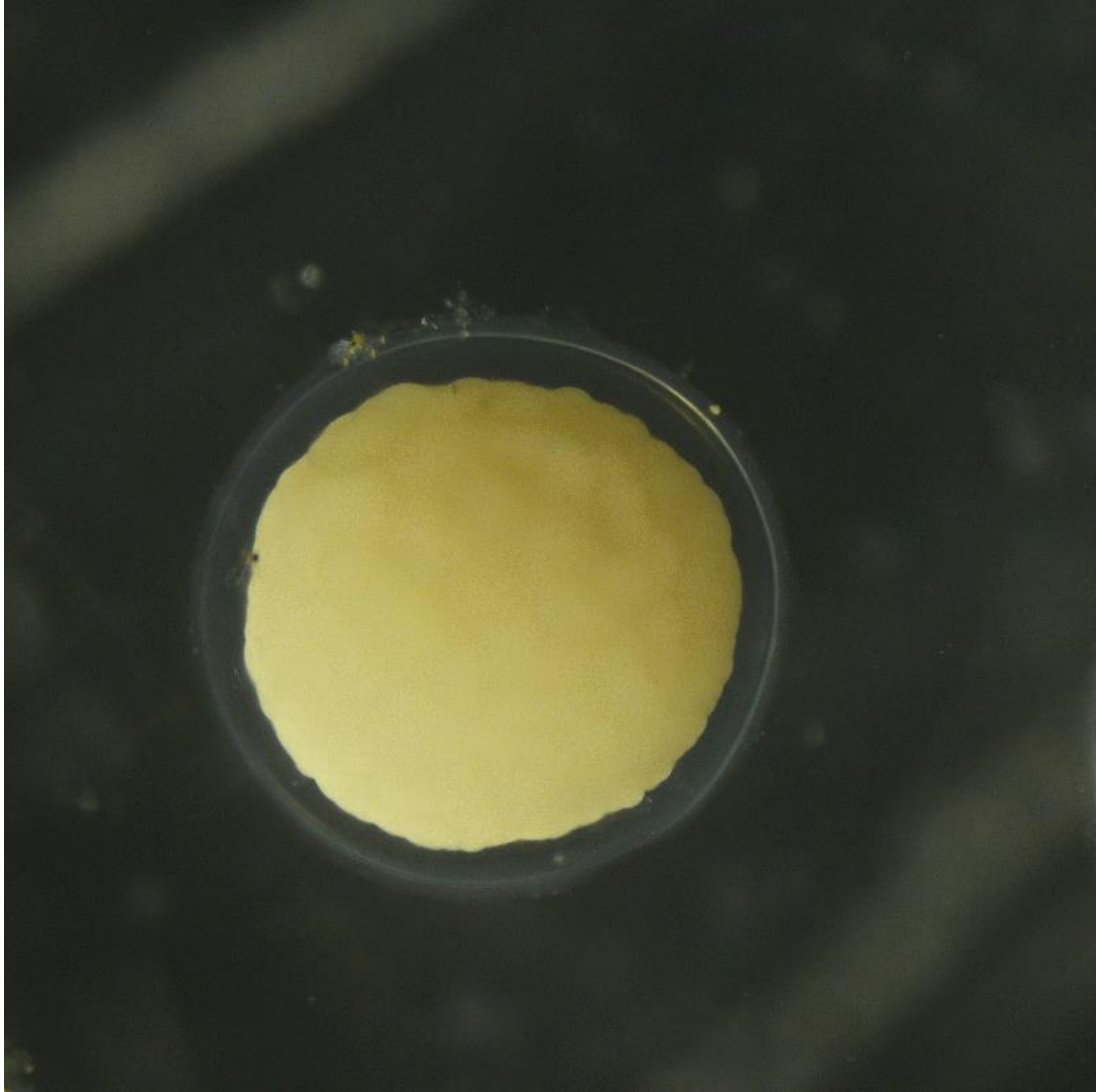

Supplementary Figure S3, continued

Figure7c（control）

gastrula

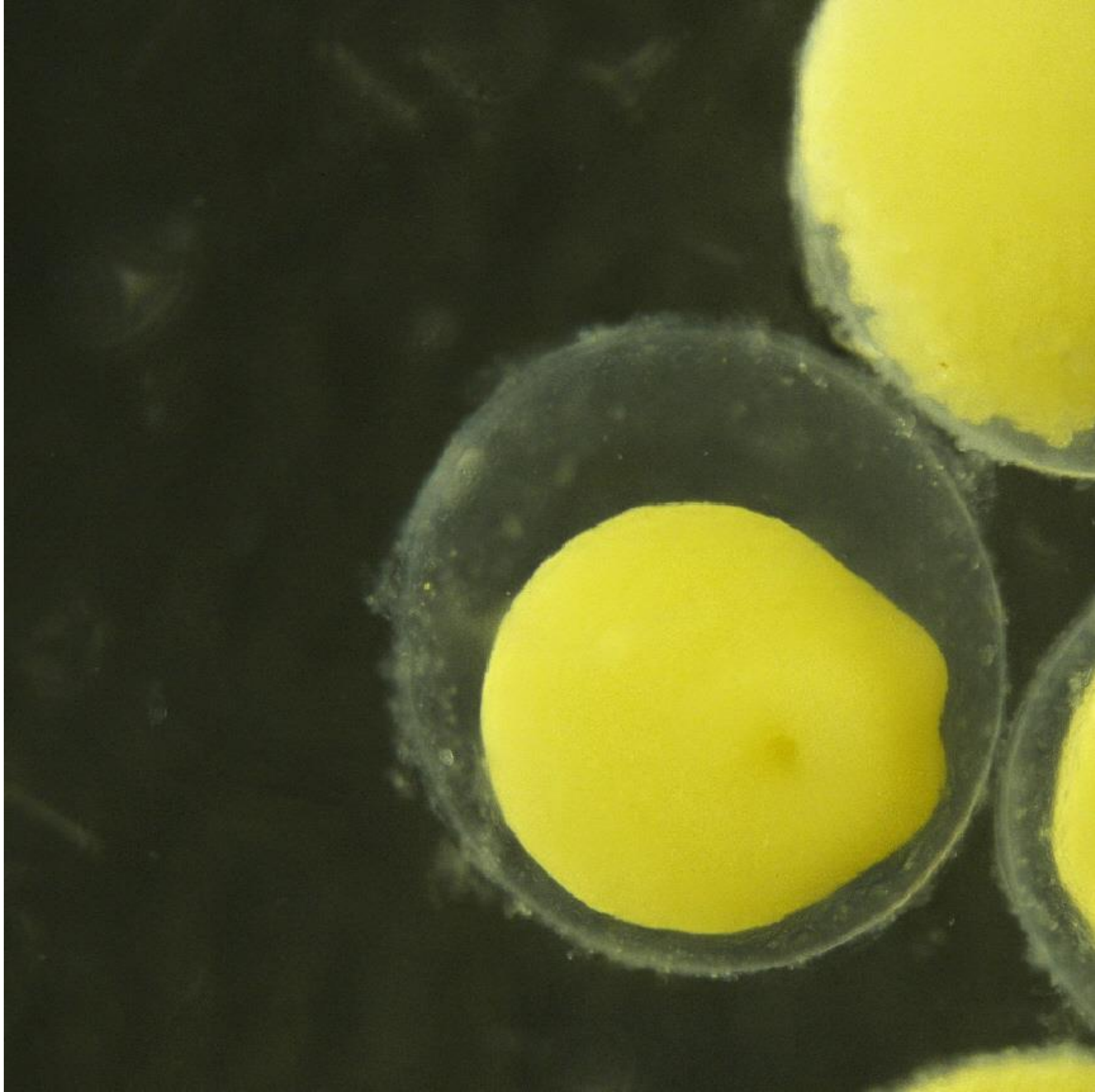

Supplementary Figure S3, continued

Figure7c（control）

neurual

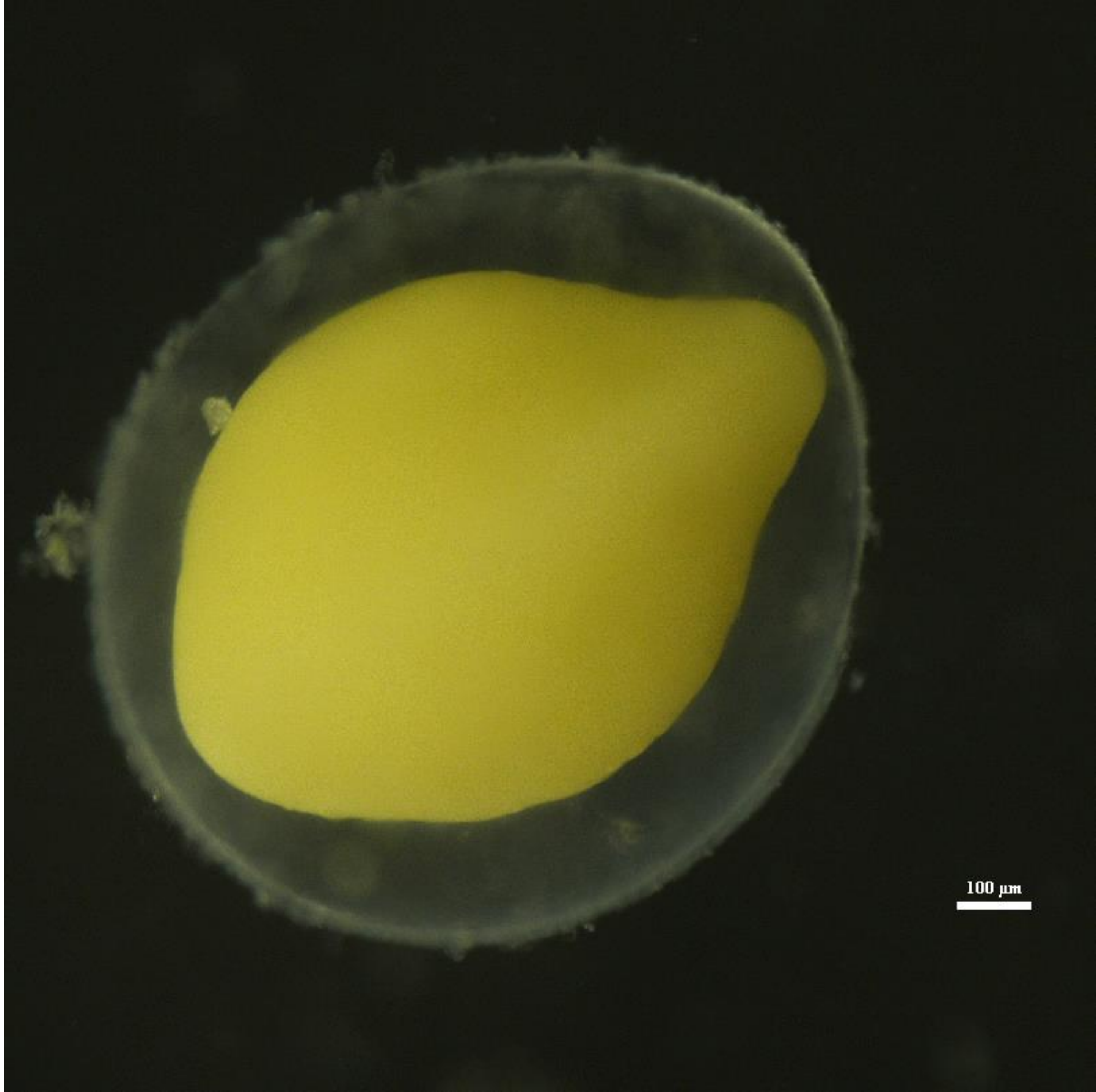

Supplementary Figure S3, continued

Figure7c（control）

head

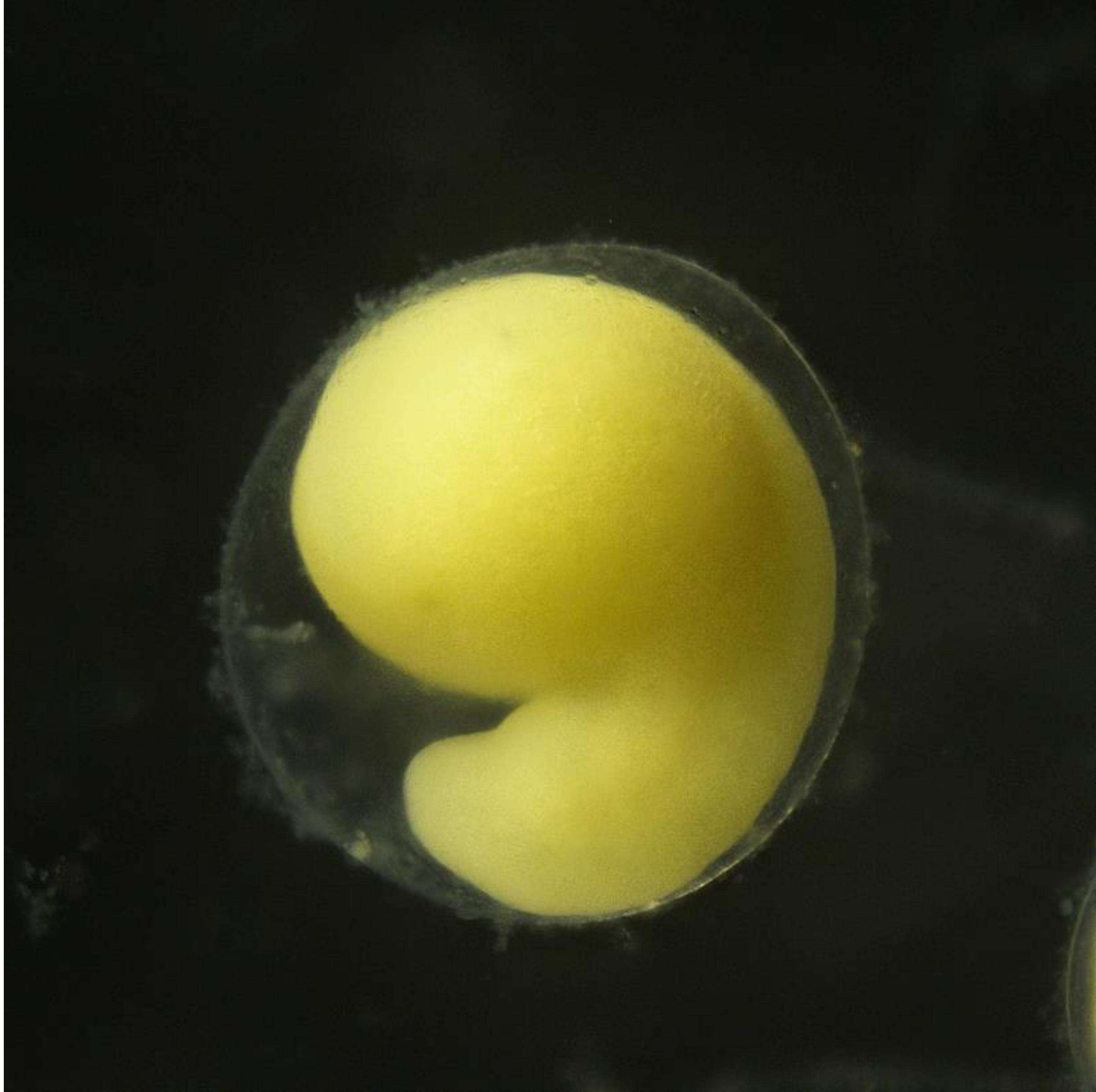

Supplementary Figure S3, continued

Figure7c（control）

prehatching

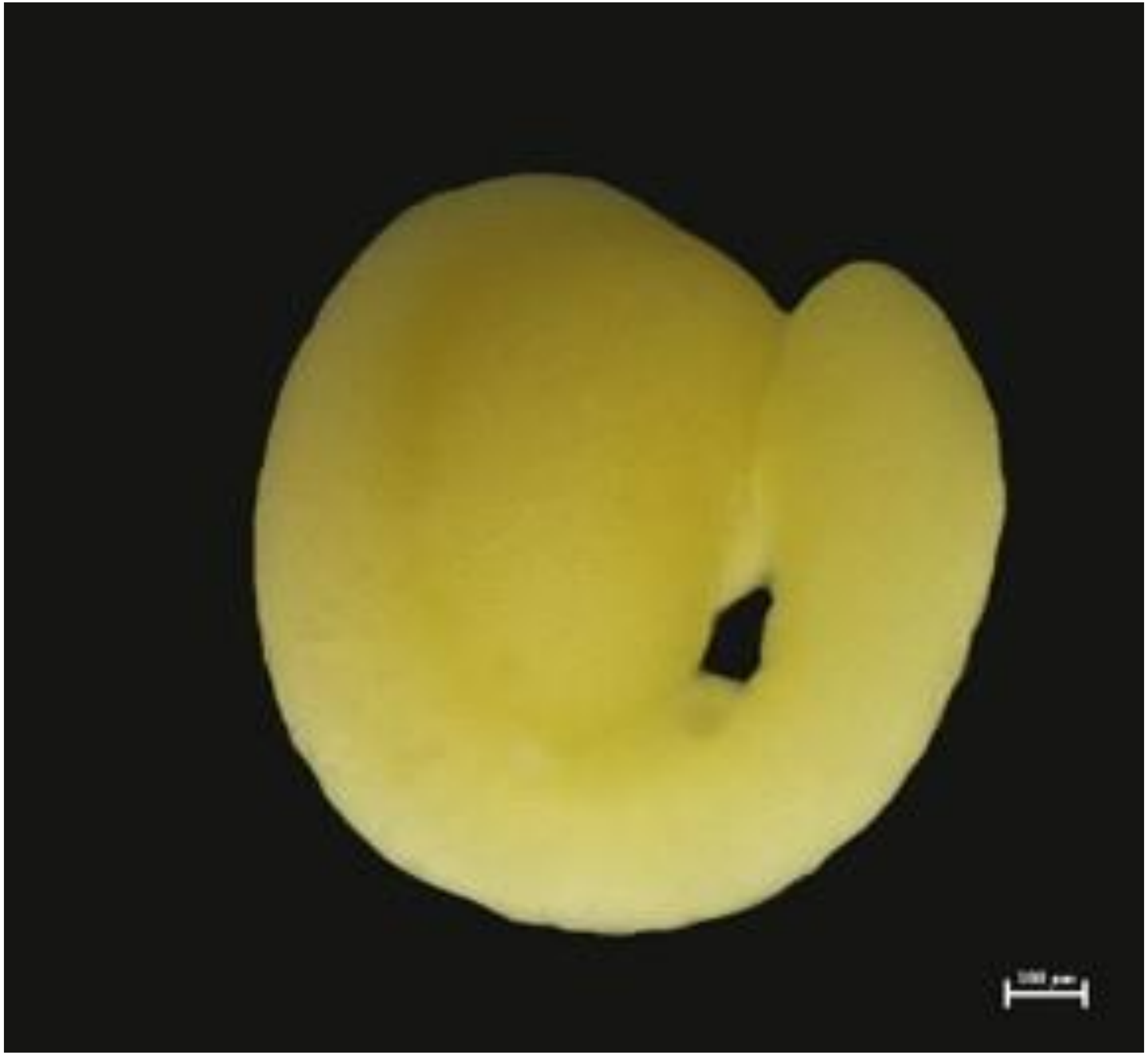

Supplementary Figure S3, continued

Figure7c（control）

prehatching

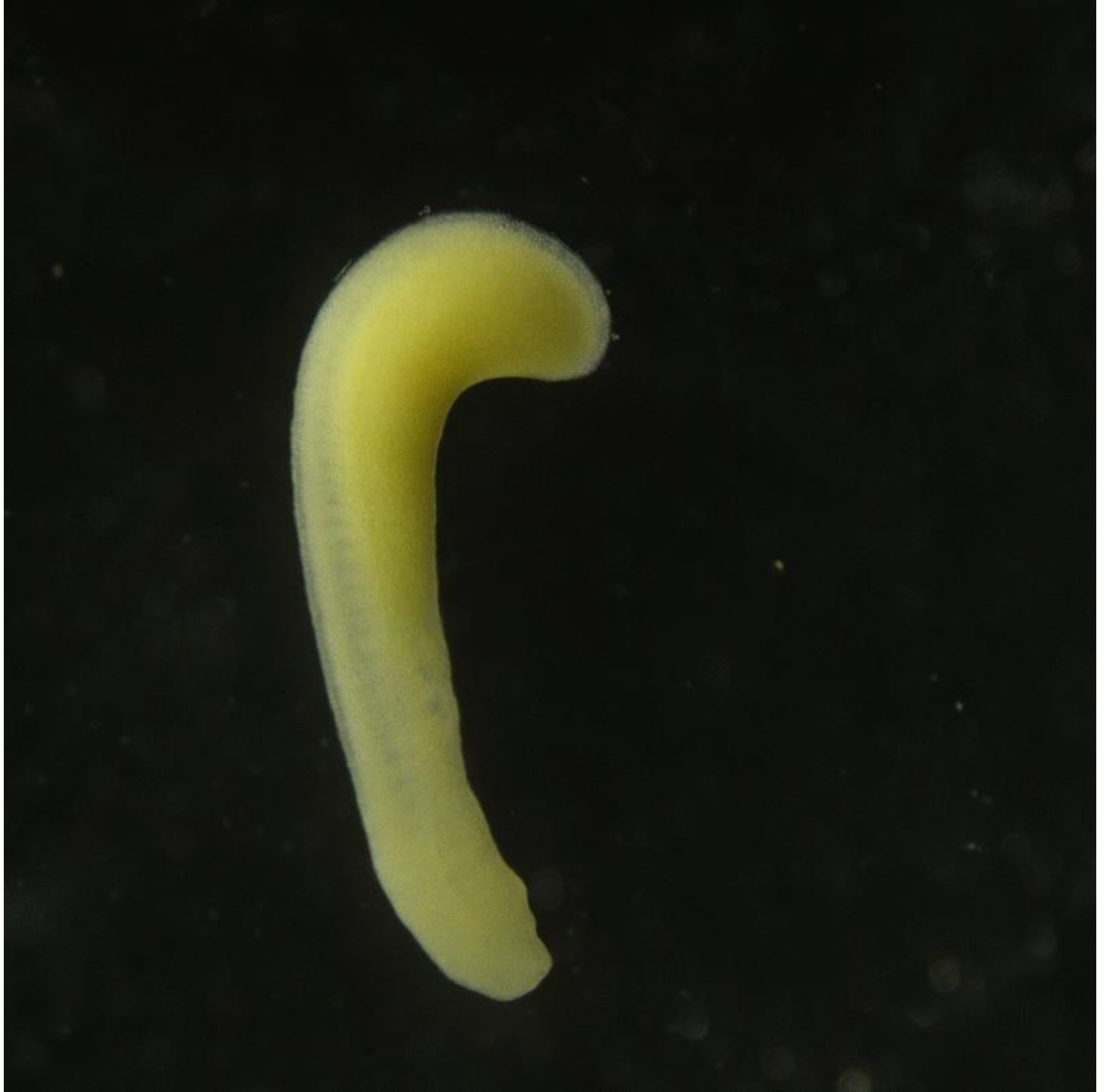

Supplementary Figure S3, continued

Figure7e(tunel-NC)

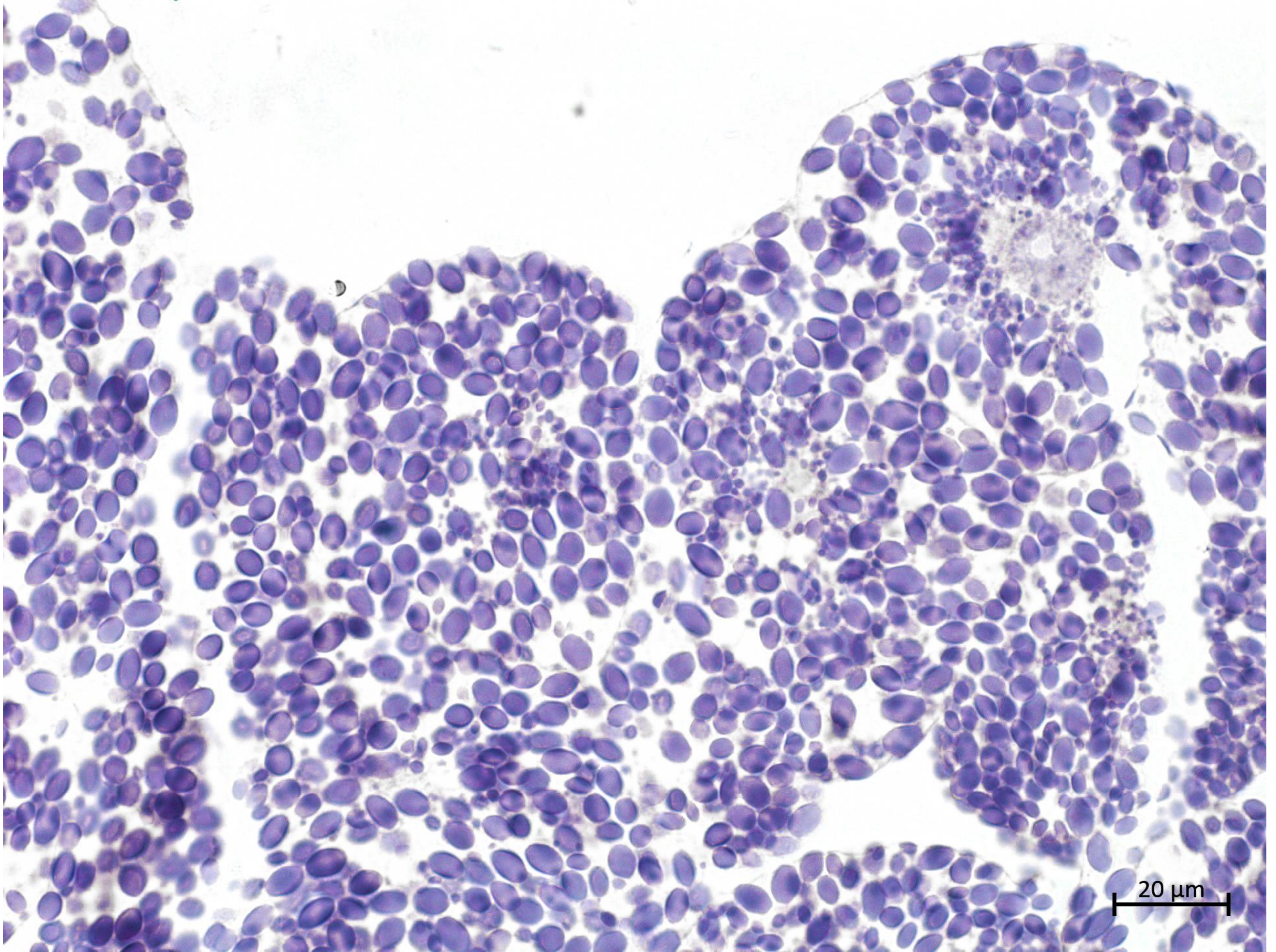

Supplementary Figure S3, continued

Figure7e(tunel-mimics)

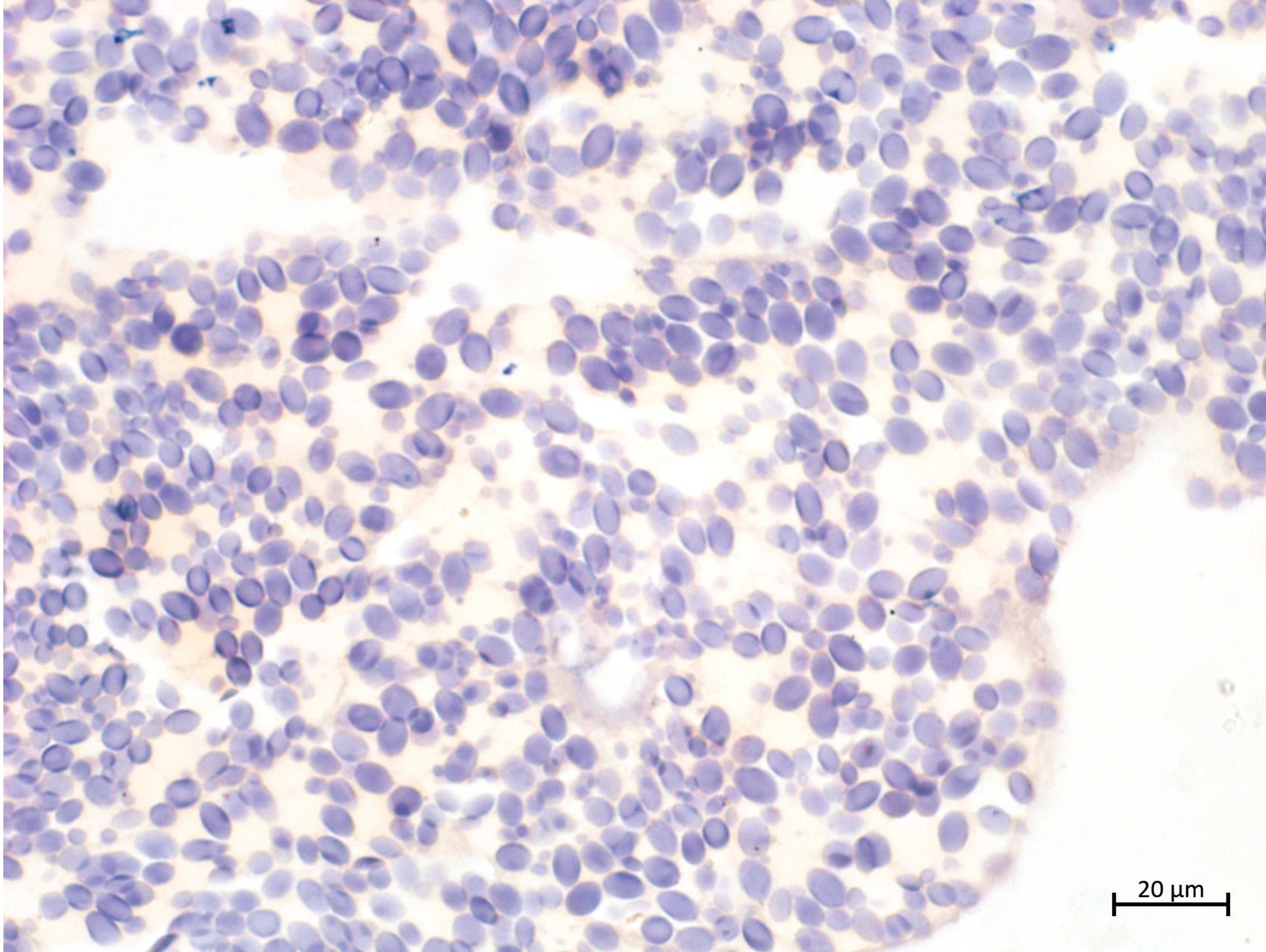

Supplementary Figure S3, continued

Figure7e(tunel-inhibitor)

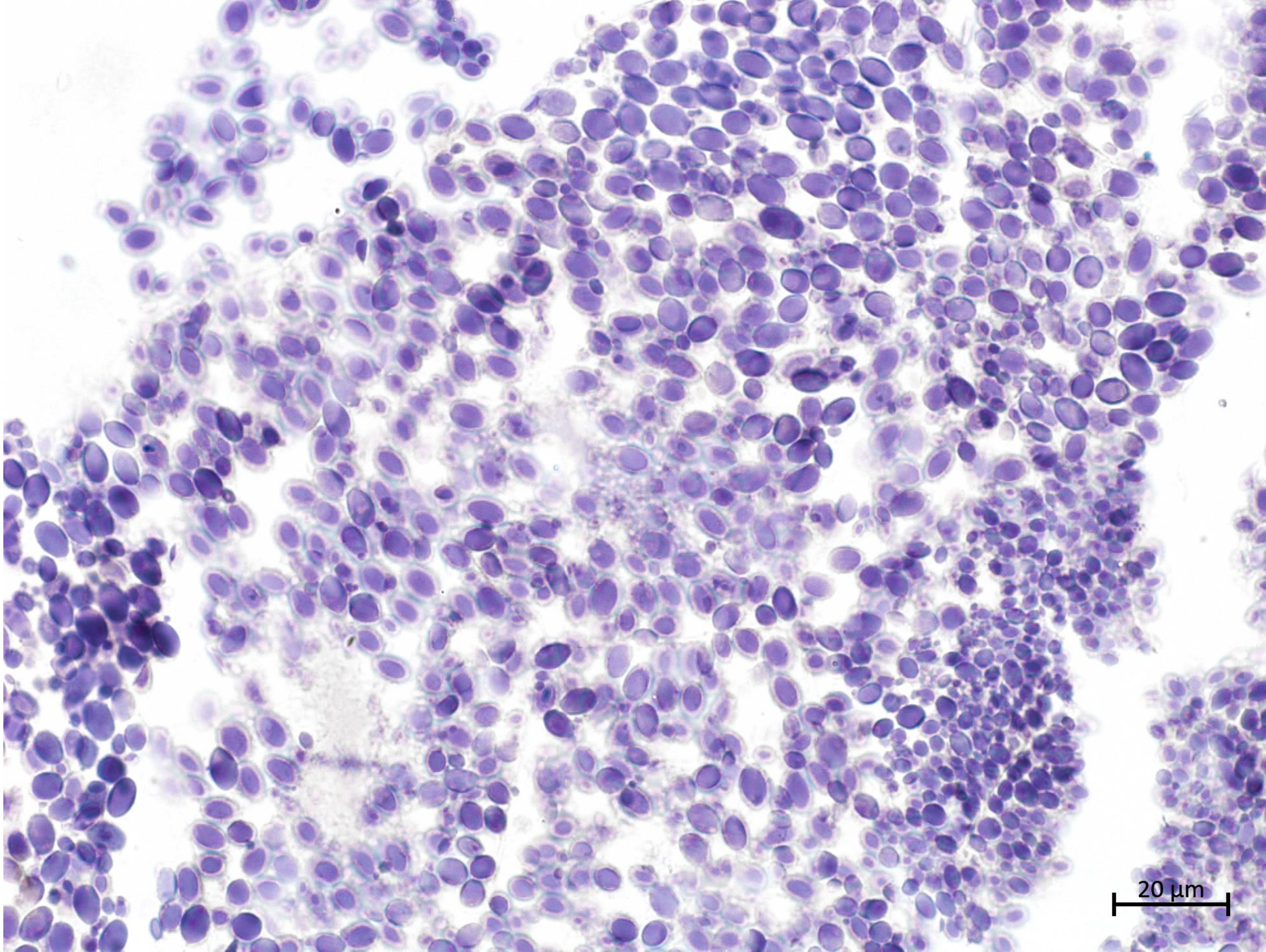

Supplementary Figure S3, continued

Figure8c(nc-64cell)

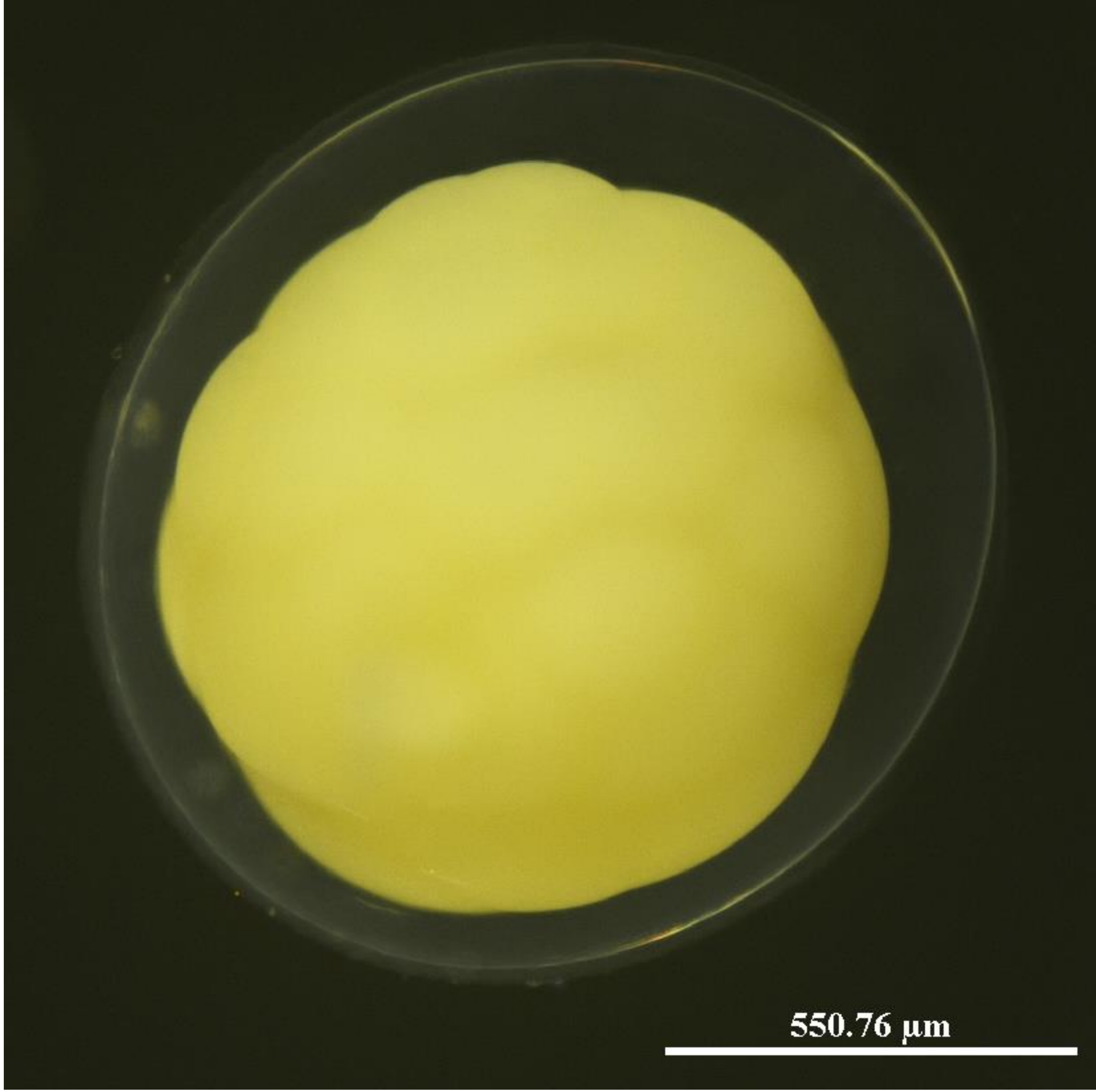

Supplementary Figure S3, continued

Figure8c(nc-neurual)

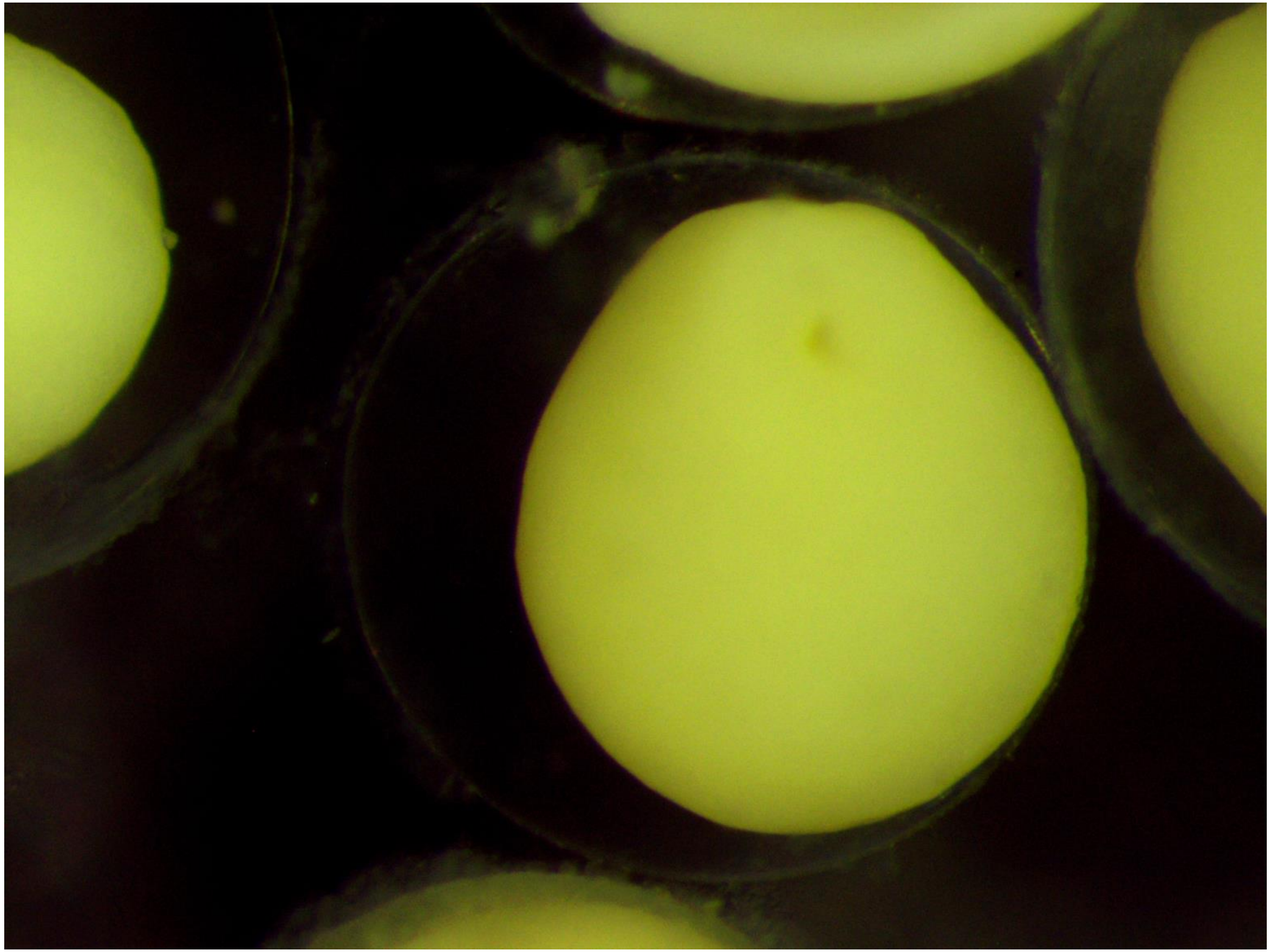

Supplementary Figure S3, continued

Figure8c(nc-head stage)

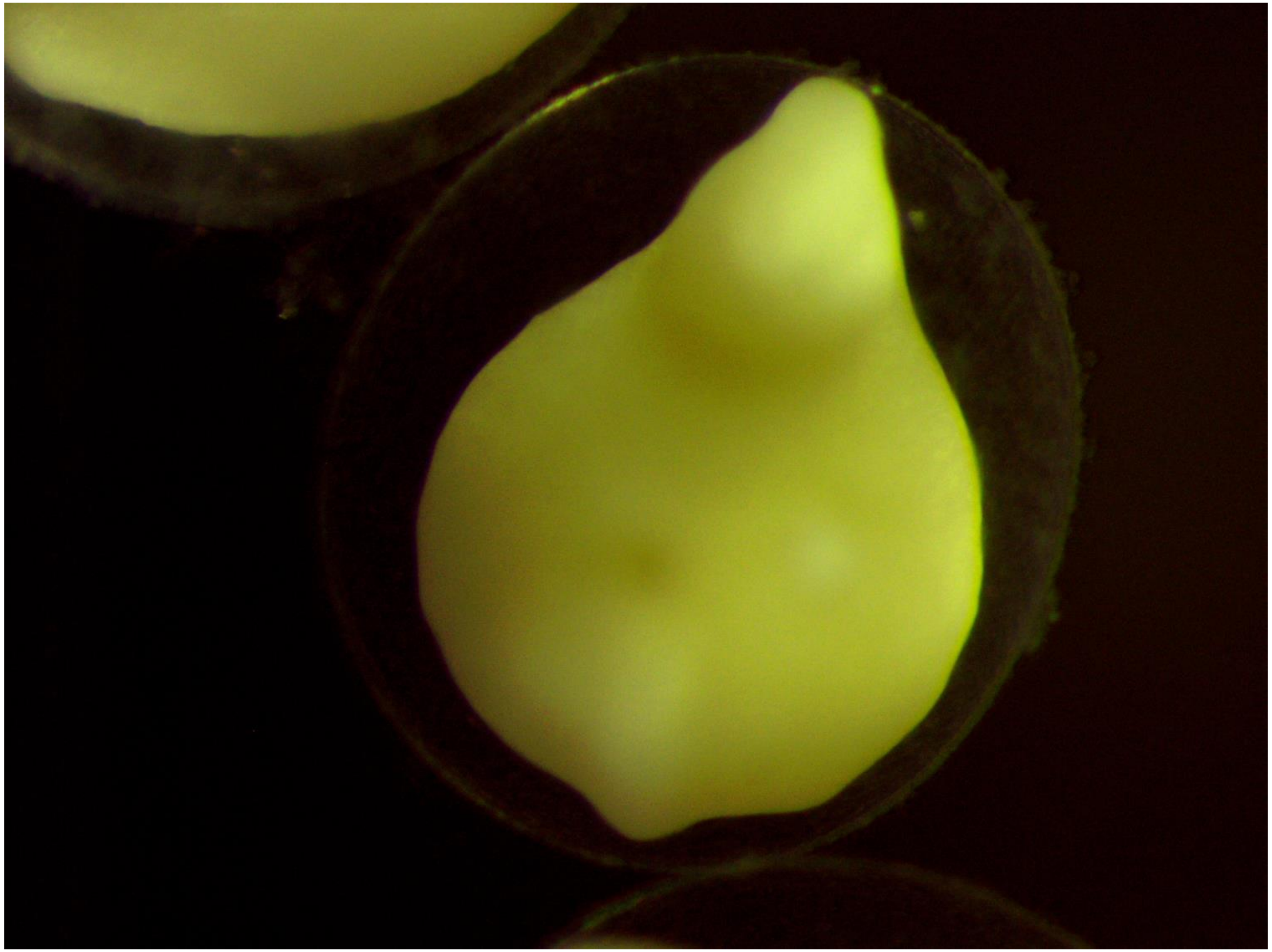

Supplementary Figure S3, continued

Figure8c(siRNANC-64cell)

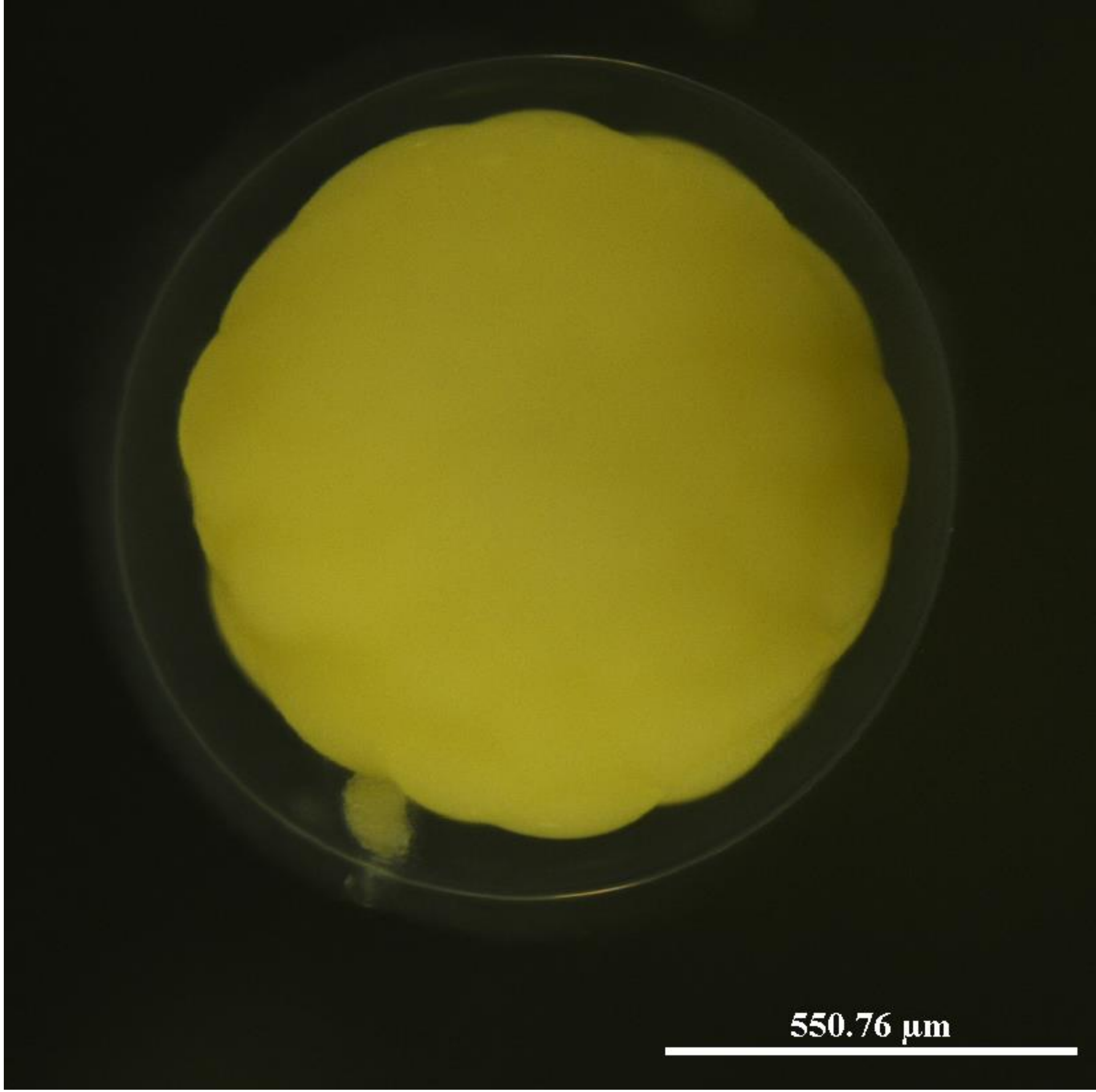

Supplementary Figure S3, continued

Figure8c(siRNANC-neurual)

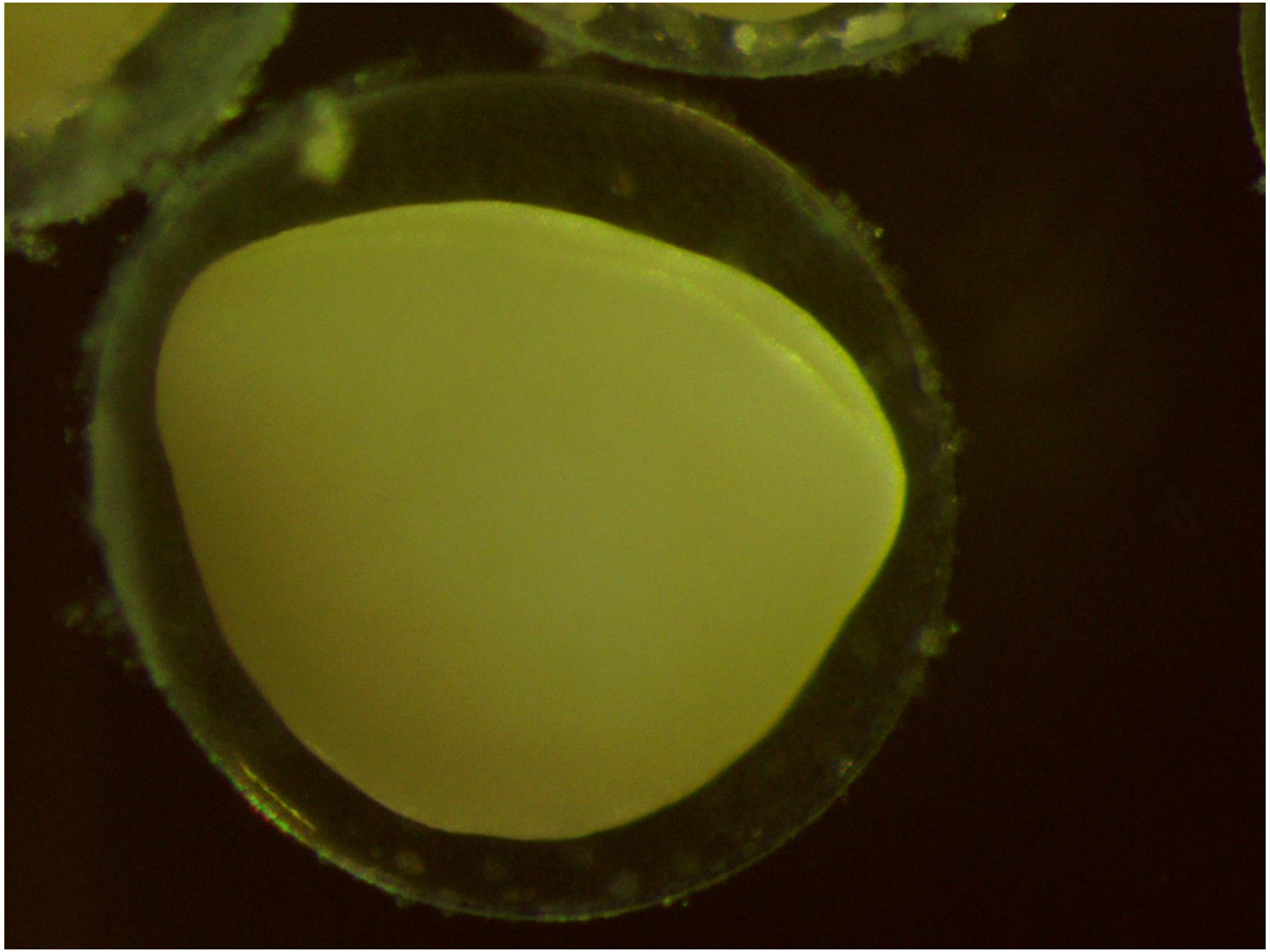

Supplementary Figure S3, continued

Figure8c(siRNANC-head stage)

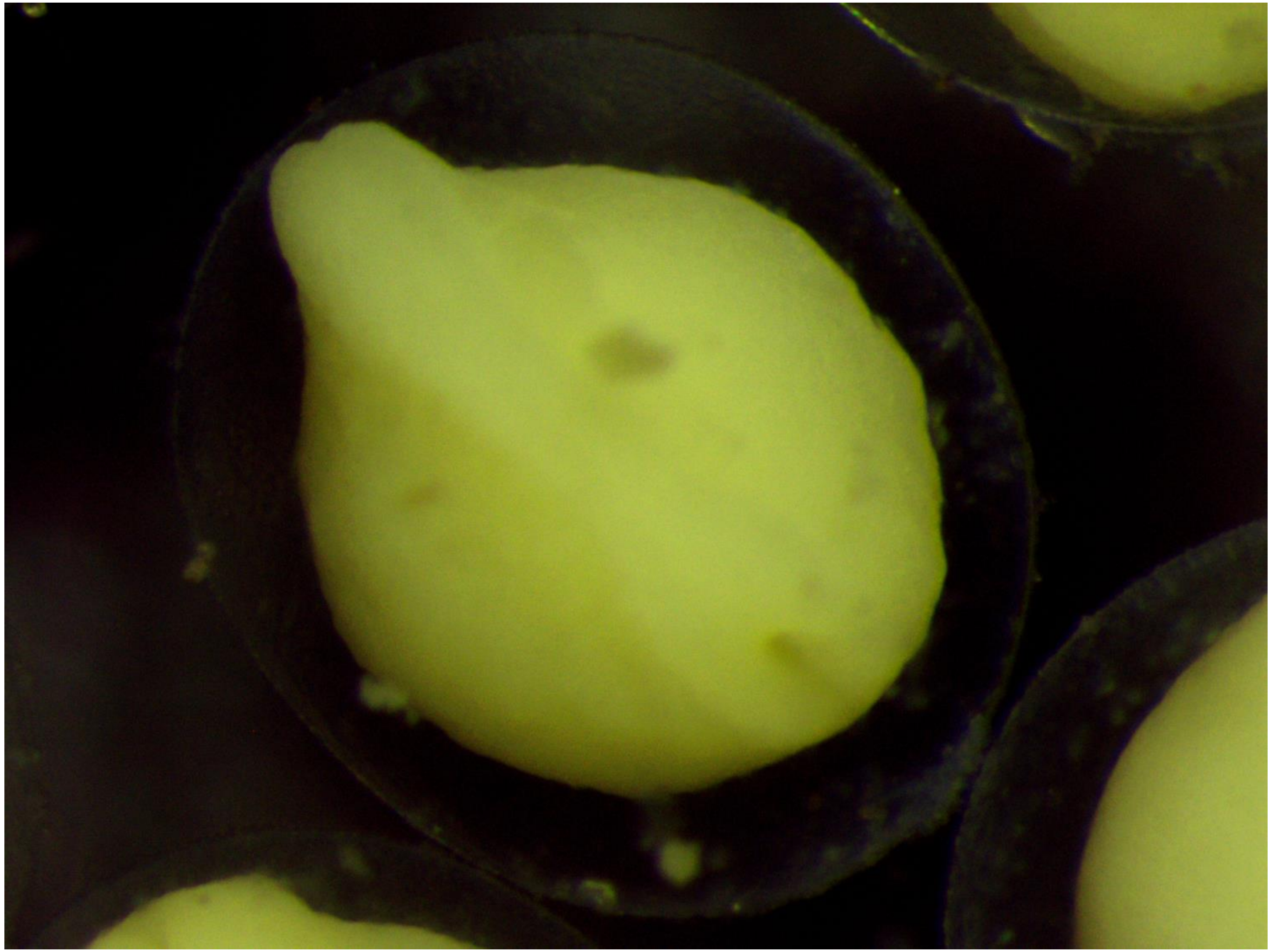

Supplementary Figure S3, continued

Figure8c(siRNALIP-64cell)

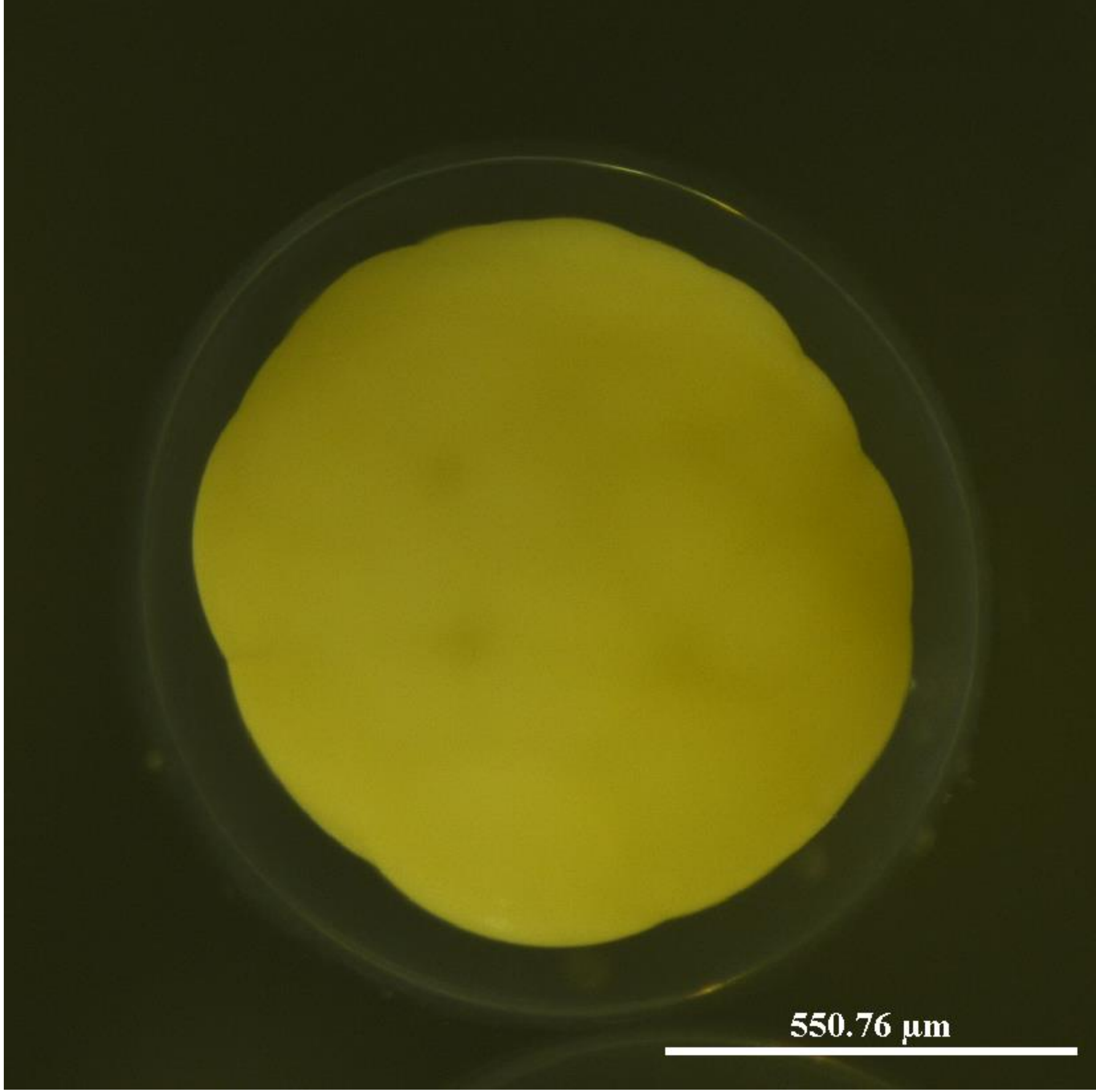

Supplementary Figure S3, continued

Figure8c(siRNALIP-neurual)

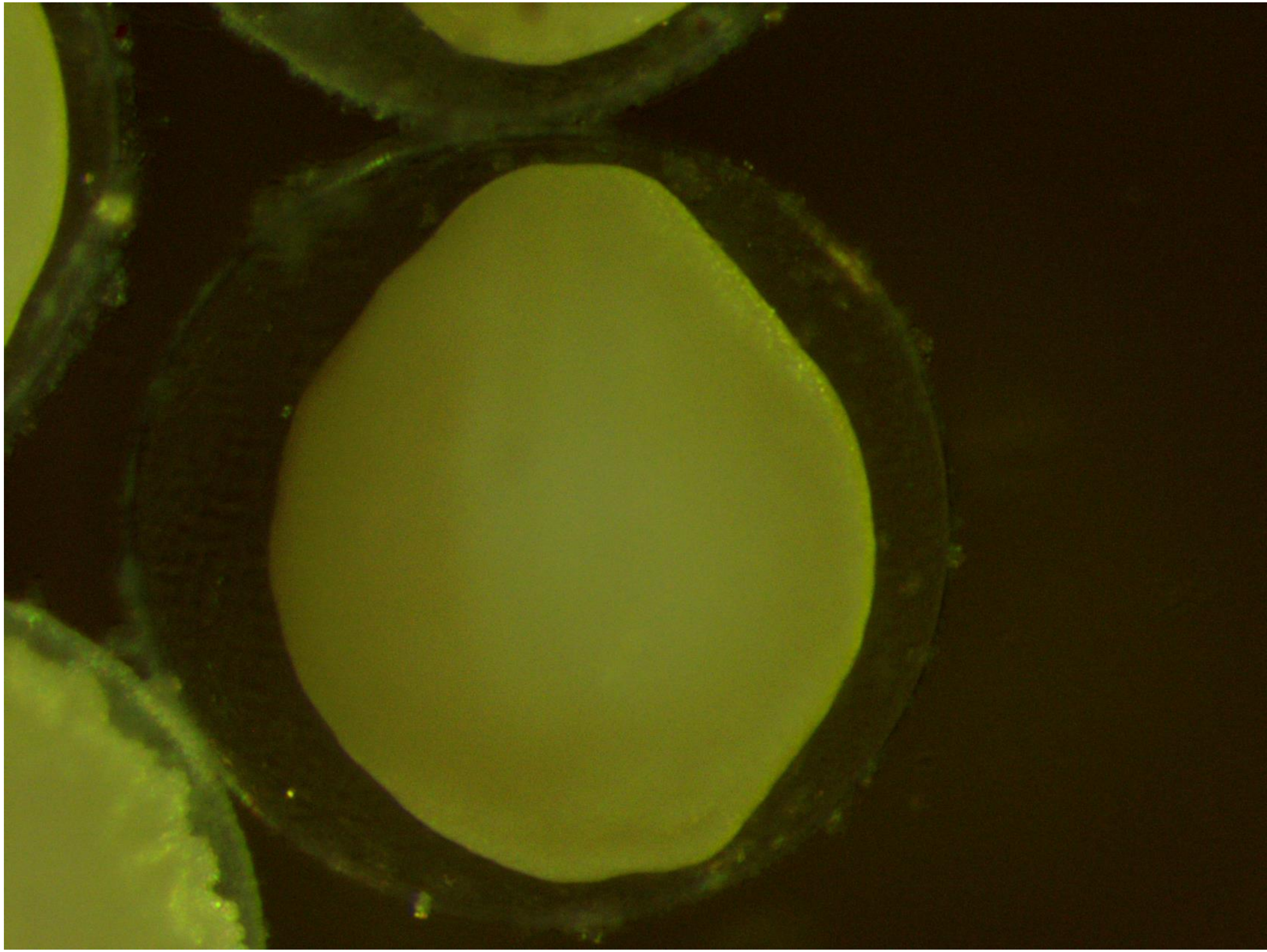

Supplementary Table S1.
Primers used in this study

Genebank No.	Primers	Primers sequence (5'→3')	length
MT176433	*lip*-F	TGGCATCTGCTTGGGTGTT	19
	lip-R	CACGTTAGGCAGAATTTGGT	20
MN395386	*tnfr*-F	TTGCCTCCCCAAGCTGATAC	20
	tnfr-R	ACATCCTGCAGCGTGACAGA	20
AEH59759	*hmgb*1-F	CCCGTCGGCTTTCTTCATC	19
	*hmgb*1-R	TTCCACATCTCACCCAGTTTCTT	23
AY578058	*gapdh*-F	AGGTGAAGGTCGGAGTCAACGGA	23
	gapdh-R	TCAAAGGTGGAGGAGTGGGTGTC	23
	miR-4561-F	TCGGCAGGUUUGGUGAUGAUUU	22
	miR-4561-R	CTCAACTGGTGTCGTGGA	18
	U6-F	CGCTTCGGCAGCACATATAC	20
	U6-R	TTCACGAATTTGCGTGTCATC	21
	RT-miR-4561	CTCAACTGGTGTCGTGGAGTCG GCAATTCAGTTGAGCCGTCATCA	45
	tnfr10-F	AGAACTCCATGATGATGCAACAA	23
MN395388	*tnfr10*-R	AAGGCTGGGCATTTGGATACT	21
	tnfr1like-F	AGCCCTTTCTGTTGCAAGCT	20
MN395389	*tnfr1like*-R	GGGCTGGCATTTGAAGCAT	19
	caspase1-F	CCAAAGCGCAGAGGAGATTG	20
MN832741	*caspase1*-R	TCGATGCTCAGCAGGTCACT	20
	caspase3-F	CGAACCTCAGCAGCAGCTGTCAGT	
MN832743	*caspase3*-R	TGTGCTGTTTGTCCCTCGAA	20
	caspase7-F	TCCCAGTGGAGGCAGACTTC	20
MN832747	*caspase7*-R	GCAGCTGCATCAACTCCAAA	20
	caspase8-F	GCGCAACGACCTTGTCAAA	19
MN832744	*caspase8*-R	GAGTGAGGACCGGGATATCG	20
MN988679	*FADD*-F	CCAGAGACAAACTCGACGCC	20
	FADD-R	CCTCCATCAGCGTGAAGAGC	20
	PDCD5-F	TCCTCGCCGACGAAGCAGACAG	22
MK716281	*PDCD5*-R	GCCACAACCTGTCAGTATTAGC	22

Supplementary Table S2. 0h vs 8h differential expression miRNA

sRNA	8h	0h	log2.Fold_change.	p.value	q.value.
novel_2	4493.07	16750.89	-1.8985	0	0
pma-miR-23b	6642.665	15722.51	-1.243	0	0
pma-miR-4561	16400.42	50160.17	-1.6128	0	0
pma-miR-451	10109.08	4202.178	1.2664	3.93E-257	3.39E-256
novel_8	6.506038	315.2982	-5.5988	3.16E-88	2.27E-87
pma-miR-497-5p	50.7471	168.8272	-1.7341	3.26E-24	1.28E-23
novel_73	141.8316	18.50161	2.9385	4.59E-19	1.65E-18
novel_60	1073.496	512.6487	1.0663	9.91E-19	3.28E-18
novel_38	977.207	467.1656	1.0647	4.01E-17	1.23E-16
pma-miR-497-3p	19.51812	79.40274	-2.0244	5.59E-14	1.60E-13
novel_94	11.71087	0	4.5498	0.0015053	0.0027006
novel_14	13.01208	26.21061	-1.0103	0.0026814	0.0044406
novel_77	11.71087	0	3.9252	0.0026722	0.0044406

Supplementary Table S3. 17d vs 0h differential expression miRNA

sRNA	17d	0h	log2.Fold_change.	p.value	q.value..
novel_2	8611.567	25381.39	-1.5594	0	0
novel_41	11124.39	5551.924	1.0027	0	0
novel_60	2076.624	776.7787	1.4187	0	0
pma-miR-4561	8497.834	76004	-3.1609	0	0
pma-miR-23b	9671.287	23823.16	-1.3006	7.23E-219	1.36E-218
novel_8	6.690154	477.7481	-6.1581	2.71E-90	3.58E-90
novel_7	515.1419	209.0878	1.3009	6.88E-80	8.26E-80
novel_37	183.3102	68.91721	1.4114	2.04E-31	1.80E-31
novel_14	97.67625	39.715	1.2983	1.81E-16	1.50E-16
pma-miR-144-5p	644.9309	1418.059	-1.1367	9.40E-08	5.91E-08
novel_35	17.3944	0	5.1206	4.00E-07	2.40E-07
pma-miR-132-5p	24.08456	109.8003	-2.1887	5.61E-07	3.22E-07
pma-miR-4543	42.81699	151.8515	-1.8264	3.45E-06	1.90E-06
novel_23	14.71834	2.336177	2.6554	3.08E-05	1.57E-05
novel_83	17.3944	4.672353	1.8964	5.60E-05	2.74E-05
novel_115	10.70425	0	4.4201	7.57E-05	3.45E-05
novel_98	10.70425	0	4.4201	7.57E-05	3.45E-05
novel_17	17.3944	7.00853	1.3114	0.0004857	0.00021398
novel_74	8.028185	1.168088	2.7809	0.0018013	0.00076798
novel_65	0	12.84897	-4.6836	0.0019732	0.000815
novel_24	0	11.68088	-4.5461	0.003561	0.0014262
novel_69	1.338031	14.01706	-3.389	0.0069465	0.0027003
novel_31	5.352123	0	3.4201	0.0071573	0.0027028
novel_13	0	8.176618	-4.0315	0.021411	0.0074472

Supplementary Table S4. 17d vs 8h differential expression miRNA

sRNA	B17	B8	log2.Fold_change.	p.value	q.value..
novel_1	639462.5	1356331	-1.0848	0	0
novel_4	1937.888	642.7728	1.5921	0	0
novel_7	474.007	87.21038	2.4423	1.74E-138	2.32E-138
pma-miR-497-5p	422.2971	62.98527	2.7452	2.13E-131	2.53E-131
pma-miR-4561	7819.269	20355.55	-1.3803	1.45E-88	1.41E-88
novel_37	168.6726	30.68513	2.4586	1.16E-50	9.50E-51
novel_62	188.3716	90.44039	1.0585	1.44E-33	1.09E-33
novel_14	89.87665	16.15007	2.4764	6.71E-28	4.21E-28
novel_73	18.46781	176.0358	-3.2528	2.19E-17	1.30E-17
pma-miR-29a-5p	1460.188	3790.421	-1.3762	2.45E-17	1.37E-17
pma-miR-130b	43.09155	11.30505	1.9304	1.97E-12	1.00E-12
pma-miR-497-3p	51.70985	24.2251	1.0939	1.62E-10	7.19E-11
novel_35	16.00543	0	5.0005	2.89E-07	1.23E-07
novel_17	16.00543	4.845021	1.724	3.55E-05	1.40E-05
pma-miR-132-5p	22.16137	101.7454	-2.1988	4.73E-05	1.74E-05
novel_115	9.849496	0	4.3	5.91E-05	1.97E-05
novel_23	13.54306	3.230014	2.0679	5.56E-05	1.97E-05
novel_98	9.849496	0	4.3	5.91E-05	1.97E-05
novel_94	0	14.53506	-4.8615	0.0014715	0.00047589
novel_16	8.618309	3.230014	1.4159	0.004475	0.0013646
novel_24	0	11.30505	-4.4989	0.0068537	0.0020318
novel_13	0	9.690042	-4.2765	0.0149	0.0042979
novel_111	4.924748	1.615007	1.6085	0.024972	0.0070134

Supplementary Table S5 miRNA target gene prediction

miRNA	Putative target genes
novel_8	CCNI AR6P1 AMPD3 LURA1 ZN629 FDFT MYDGF
miR-4546	SYTC HSP7C MCL1 PHYD1 IF2 PHYD1
miR-497-3p	VIP2 THOC2 CLVS2
novel_2	VIP2 THOC2
miR-497-5p	HSP7C PHYD1 IL-8
**miR-4561**	MYH7 CL16A SND1 UBA1 EF2 TBA1 SCFD1 **LIP**
miR-23b	ZDH17 HSP7C RUSD3 SSXT IL8 ICA69
miR-130b	ZN300 HS90A HSP7C TSN1 SFR1 SFR1 IL8
novel_14	IF2 CLIP1 ANK2 EIF3A CC152
miR-4543	CCSAP JDP2 EIF3A TIMP3 ZN233
novel_7	SFR1 CD22 IL8
novel_37	UBIQP TRA2A RUSD3 DJC24 ASHWN CD22 IL8 T251A
novel_49	ICA69 ANK2
miR-144-5p	ZDH17 CFA36 YSM6 GBP5 ID4 CLTR1 S1PR3 TFAM PS11B MMGT1
novel_48	HSP7C CD22 CHIA RETN CHIA
novel_4	IL8 YSM6 ZN300 ZN112 TIMP3
novel_111	ZDH17 HSP7C TSN1 RUSD3 SFR1 IL8 CLIP1 ANK2 CHIA LURA1
novel_114	RUSD3 PHYD1 LRC14 IL8 ANR37 THOC2
novel_62	ZDH17 CC152 JDP2 LURA1 CD63
novel_16	CC152 RARB
novel_21	TIMP2 CFDP2 IF
novel_39	SFR1 COA3 MMP2
novel_17	HSP7C CC152 ANXA6 BAF CYT RARB
novel_69	SYTC POLR
miR-23a-3p	ZDH17 HSP7C RUSD3 SSXT IL8 ICA69
miR-144-3p	DCP1A ZN236 IL8 T251A ICA69 ANXA6 JDP2 STN1 SENP8 CYT
novel_32	RUSD3 LRC14 ZN300 SENP8 TIMP3
miR-132-3p	JDP2 TNF6B RARB CLTR1
novel_81	ZDH17 PHYD1 SSXT IL8 CC152 ZN112 ASHWN RARB BGAL
novel_46	LBR SFR1 IL8 YSM6 ICA69
miR-132-5p	PRC1 ANK2
novel_24	PHYD1 CYT ICA69 ANXA6 STN1
miR-23a-5p	RUSD3 IF2
novel_61	RETN JDP2 TIMP3
novel_78	SFR1 CD22 IL8 ZN300 CC152
novel_57	FR1OP RETN CLTR1
miR-210	TSN1 VIP2 ASHWN IL8 ANK2
novel_1	EIF3A LITAF GBP5 CH1B2
novel_13	RUSD3 PHYD1 LITAF
miR-29a-5p	RUSD3 VIP2 CYT
novel_104	HS90A UBIQP MED1 ITA8 NUCB2 SMN CXCR2 PMP22 MMGT1 ZDH17
novel_23	ZDH17 CH1B2 ASHWN MED1 CENPN DAP1
novel_87	CFA36 SFR1 ICA69 ZN300 ANR37
novel_41	ASHWN IF2 CD22 PRC1 CD22 IL8 IRF6
novel_67	DAP1 CFDP2 HSC20 BORG2 CFAD COQ9 TNIP1
novel_38	IL8 YSM6 YSM6 ICA69 TNF6B FANCM GRN
miR-451	CD22 IL8 ICA69 RARB UBIQP ZHX1 VDAC3
novel_83	TSN1 IL8 MMP2 ICA69 ANR37 IF BUB1
novel_74	CD22 EIF3A TECT3
novel_73	GP146 UBTD2
novel_77	BLNK CD63 UBTD2 SAP SQSTM

Supplementary Table S6 Statistics of Embryo hatching rate

Batch number	Embryo number	Control group	Mimics group	Inhibitor group
1	1000	0.50	0.29	0.40
2	1000	0.35	0.21	0.31
3	1000	0.42	0.15	0.40
4	1000	0.36	0.18	0.21
5	1000	0.15	0.05	0.07
6	1000	0.19	0.15	0.17
7	1000	0.39	0.17	0.29
8	1000	0.22	0.06	0.15
9	1000	0.33	0.10	0.17
10	1000	0.42	0.05	0.11
11	1000	0.12	0.03	0.13
12	1000	0.15	0.04	0.08
13	1000	0.20	0.10	0.15

Supplementary Table S7. Top 10 highest expressed miRNAs in immune group

0h group		8h group		17d group	
readcount	microRNA	readcount	microRNA	readcount	microRNA
991192	novel_1	839830	novel_1	519387	novel_1
65067	pma-miR-4561	12604	pma-miR-4561	8314	novel_41
21729	novel_2	7769	pma-miR-451	7228	pma-miR-23b
20395	pma-miR-23b	5131	novel_41	6545	pma-miR-451
7290	pma-miR-23a-3p	5105	pma-miR-23b	6436	novel_2
5451	pma-miR-451	4248	pma-miR-23a-3p	6351	pma-miR-4561
4753	novel_41	3453	novel_2	5100	pma-miR-23a-3p
2509	pma-miR-29a-5p	2347	pma-miR-29a-5p	1574	novel_4
1279	novel_57	1063	novel_57	1552	novel_60
1214	pma-miR-144-5p	825	novel_60	1186	pma-miR-29a-5p
1196	novel_4	751	novel_38	797	novel_57
