# Supplementary material for: MicroRNA expression profile in Lampetra morii upon Vibrio anguillarum infection and miR-4561 characterization targeting lip
Source: Commun Biol. 2021 Aug 20;4:995. doi: 10.1038/s42003-021-02525-z (PMC8379177; doi:10.1038/s42003-021-02525-z)
Supplement: Supplementary file 3 — Description of Additional Supplementary Files. [file 42003_2021_2525_MOESM3_ESM.pdf]

Description of Additional Supplementary Files

File name: Supplementary Data 1

Description: Source data for graphs and charts in this study.
