# Supplementary material for: MicroRNA expression profile in Lampetra morii upon Vibrio anguillarum infection and miR-4561 characterization targeting lip
Source: Commun Biol. 2021 Aug 20;4:995. doi: 10.1038/s42003-021-02525-z (PMC8379177; doi:10.1038/s42003-021-02525-z)
Supplement: Supplementary file 5 — Reporting summary. [file 42003_2021_2525_MOESM5_ESM.pdf]

nature research

Corresponding author(s): Yue Pang

Last updated by author(s): Jul 25, 2021

Reporting Summary

Nature Research wishes to improve the reproducibility of the work that we publish. This form provides structure for consistency and transparency in reporting. For further information on Nature Research policies, see our Editorial Policies and the Editorial Policy Checklist.

Statistics

For all statistical analyses, confirm that the following items are present in the figure legend, table legend, main text, or Methods section.

n/a	Confirmed	
☐	☒	The exact sample size (n) for each experimental group/condition, given as a discrete number and unit of measurement
☐	☒	A statement on whether measurements were taken from distinct samples or whether the same sample was measured repeatedly
☐	☒	The statistical test(s) used AND whether they are one- or two-sided<br>*Only common tests should be described solely by name; describe more complex techniques in the Methods section.*
☐	☒	A description of all covariates tested
☐	☒	A description of any assumptions or corrections, such as tests of normality and adjustment for multiple comparisons
☐	☒	A full description of the statistical parameters including central tendency (e.g. means) or other basic estimates (e.g. regression coefficient) AND variation (e.g. standard deviation) or associated estimates of uncertainty (e.g. confidence intervals)
☐	☒	For null hypothesis testing, the test statistic (e.g. F, t, r) with confidence intervals, effect sizes, degrees of freedom and P value noted<br>*Give P values as exact values whenever suitable.*
☐	☒	For Bayesian analysis, information on the choice of priors and Markov chain Monte Carlo settings
☐	☒	For hierarchical and complex designs, identification of the appropriate level for tests and full reporting of outcomes
☐	☒	Estimates of effect sizes (e.g. Cohen's d, Pearson's r), indicating how they were calculated

Our web collection on statistics for biologists contains articles on many of the points above.

Software and code

Policy information about availability of computer code

Data collection	No unreported custom computer code or algorithm used to generate results that are reported in the paper.
Data analysis	Mapped small RNA tags were compared to known miRNA. Differential expression analysis between two samples was performed using the DEGseq (2010) package for R. The secondary structures of the candidate miRNAs were generated using minimum free energy (MFE) and the partition function algorithm on the RNAfold software (http://rna.tbi.univie.ac.at/cgi-bin/RNAWebSuite/RNAfold.cgi).

For manuscripts utilizing custom algorithms or software that are central to the research but not yet described in published literature, software must be made available to editors and reviewers. We strongly encourage code deposition in a community repository (e.g. GitHub). See the Nature Research guidelines for submitting code & software for further information.

Data

Policy information about availability of data

All manuscripts must include a data availability statement. This statement should provide the following information, where applicable:

- Accession codes, unique identifiers, or web links for publicly available datasets
- A list of figures that have associated raw data
- A description of any restrictions on data availability

All relevant data are available from the corresponding author upon reasonable request. Source data underlying plots shown in figures are provided in Supplementary Information. All miRNA transcriptome data have been uploaded with the SRR accession numbers SRR11306709, SRR11306708 and SRR11306707. All source data underlying the graphs and charts shown in the main and supplementary figures are presented in Supplementary Data 1.

Field-specific reporting

Please select the one below that is the best fit for your research. If you are not sure, read the appropriate sections before making your selection.

☒ Life sciences ☐ Behavioural & social sciences ☐ Ecological, evolutionary & environmental sciences

For a reference copy of the document with all sections, see nature.com/documents/nr-reporting-summary-flat.pdf

Life sciences study design

All studies must disclose on these points even when the disclosure is negative.

Sample size	No sample-size calculation was performed in this study.
Data exclusions	No data were excluded in this study.
Replication	All experiments in this article have been replicated at least three times (n=3) .
Randomization	The lampreys used in this article are completely randomly grouped.
Blinding	The data processor and the experiment designer are different people, but this article is not a medical experiment using human volunteers, so completely double-blind cannot be achieved.

Reporting for specific materials, systems and methods

We require information from authors about some types of materials, experimental systems and methods used in many studies. Here, indicate whether each material, system or method listed is relevant to your study. If you are not sure if a list item applies to your research, read the appropriate section before selecting a response.

Materials & experimental systems

n/a	Involved in the study	
☐	☒	Antibodies
☐	☒	Eukaryotic cell lines
☒	☐	Palaeontology and archaeology
☐	☒	Animals and other organisms
☒	☐	Human research participants
☒	☐	Clinical data
☒	☐	Dual use research of concern

Methods

n/a	Involved in the study	
☒	☐	ChIP-seq
☒	☐	Flow cytometry
☒	☐	MRI-based neuroimaging

Antibodies

Antibodies used	Anti-β-actin antibody was purchased from Abcam (USA) while anti-LIP antibody was produced in our laboratory.
Validation	All antibodies are validated using LC-MS after Western-Blotting.

Eukaryotic cell lines

Policy information about cell lines

Cell line source(s)	HEK293
Authentication	Purchased from ATCC
Mycoplasma contamination	All cell lines used in this study have been confirmed negative for mycoplasma contamination.
Commonly misidentified lines (See ICLAC register)	Only HEK293 cell line used in this study,so there is no commonly misidentified lines in this study.

Animals and other organisms

Policy information about studies involving animals; ARRIVE guidelines recommended for reporting animal research

Laboratory animals	*For laboratory animals, report species, strain, sex and age OR state that the study did not involve laboratory animals.*

Wild animals	*Provide details on animals observed in or captured in the field; report species, sex and age where possible. Describe how animals were caught and transported and what happened to captive animals after the study (if killed, explain why and describe method; if released, say where and when) OR state that the study did not involve wild animals.*
Field-collected samples	Adult healthy lampreys (Lampetra morii) (weight, 35 ± 5 g) were collected from the Yalu River and temporarily raised in a culture system composed of an aquarium (100 × 50 × 50 cm), black ceramsite sand filtration system (2–3 mm3), and ceramsite sand bottom layer (12 ± 2 cm high). Water height was about 40 cm and the water flow was about 2 L/min. The animal experiments were performed in accordance with the regulations of the Animal Welfare and Research Ethics Committee of the Institute of Dalian Medical University's Animal Care protocol (Permit Number: SCXK2008-0002).
Ethics oversight	*Identify the organization(s) that approved or provided guidance on the study protocol, OR state that no ethical approval or guidance was required and explain why not.*

Note that full information on the approval of the study protocol must also be provided in the manuscript.
